# Pluridisciplinary evidence for burial for the La Ferrassie 8 Neandertal child

**DOI:** 10.1038/s41598-020-77611-z

**Published:** 2020-12-09

**Authors:** Antoine Balzeau, Alain Turq, Sahra Talamo, Camille Daujeard, Guillaume Guérin, Frido Welker, Isabelle Crevecoeur, Helen Fewlass, Jean-Jacques Hublin, Christelle Lahaye, Bruno Maureille, Matthias Meyer, Catherine Schwab, Asier Gómez-Olivencia

**Affiliations:** 1 PaleoFED Team, UMR 7194, CNRS, Département Homme et Environnement, Muséum National d’Histoire Naturelle, Musée de l’Homme, 17, Place du Trocadéro, 75016 Paris, France; 2 Department of African Zoology, Royal Museum for Central Africa, Tervuren, Belgium; 3 Musée National de Préhistoire, 1 Rue du Musée, 24620 Les Eyzies-de-Tayac, France; 4 Department of Human Evolution, Max Planck Institute for Evolutionary Anthropology, Deutscher Platz 6, 04103 Leipzig, Germany; 5 Department of Chemistry “G. Ciamician”, University of Bologna, Via Selmi, 2, 40126 Bologna, Italy; 6 UMR 5060, CNRS-Université Bordeaux Montaigne, IRAMAT-CRP2A, Maison de l’archéologie, Esplanade des Antilles, 33607 Pessac Cedex, France; 7 UMR 6118, CNRS-Univ Rennes, Géosciences Rennes, 35000 Rennes, France; 8 Department of Human Evolution, Max Planck Institute for Evolutionary Anthropology, Leipzig, Germany; 9 Section for Evolutionary Genomics, The Globe Institute, University of Copenhagen, Copenhagen, Denmark; 10 Univ. Bordeaux, CNRS, MCC, PACEA, UMR 5199, 33600 Pessac, France; 11 Department of Evolutionary Genetics, Max Planck Institute for Evolutionary Anthropology, Leipzig, Germany; 12 Musée d’Archéologie Nationale, Saint-Germain-en-Laye, France; 13 Department Geología, Facultad de Ciencia y Tecnología, Universidad del País Vasco-Euskal Herriko Unibertsitatea (UPV/EHU), Barrio Sarriena s/n, 48940 Leioa, Spain; 14 Sociedad de Ciencias Aranzadi, Zorroagagaina 11, 20014 Donostia-San Sebastián, Spain; 15 Centro UCM-ISCIII de Investigación sobre Evolución y Comportamiento Humanos, Avda. Monforte de Lemos 5 (Pabellón 14), 28029 Madrid, Spain

**Keywords:** Palaeontology, Archaeology, Molecular evolution, Geochemistry

## Abstract

The origin of funerary practices has important implications for the emergence of so-called modern cognitive capacities and behaviour. We provide new multidisciplinary information on the archaeological context of the La Ferrassie 8 Neandertal skeleton (*grand abri* of La Ferrassie, Dordogne, France), including geochronological data -^14^C and OSL-, ZooMS and ancient DNA data, geological and stratigraphic information from the surrounding context, complete taphonomic study of the skeleton and associated remains, spatial information from the 1968–1973 excavations, and new (2014) fieldwork data. Our results show that a pit was dug in a sterile sediment layer and the corpse of a two-year-old child was laid there. A hominin bone from this context, identified through Zooarchaeology by Mass Spectrometry (ZooMS) and associated with Neandertal based on its mitochondrial DNA, yielded a direct ^14^C age of 41.7–40.8 ka cal BP (95%), younger than the ^14^C dates of the overlying archaeopaleontological layers and the OSL age of the surrounding sediment. This age makes the bone one of the most recent directly dated Neandertals. It is consistent with the age range for the Châtelperronian in the site and in this region and represents the third association of Neandertal taxa to Initial Upper Palaeolithic lithic technocomplex in Western Europe. A detailed multidisciplinary approach, as presented here, is essential to advance understanding of Neandertal behavior, including funerary practices.

## Introduction

Elaborate funerary activity is unique to the human lineage^1^ and the emergence of this behavior can be framed within the broader context of the increasing complexity of cognitive and symbolic capacities. While it has been suggested that the Middle Pleistocene human bone accumulation at Sima de los Huesos could have had an anthropic origin^2–4^, relatively complete articulated hominin skeletons are extremely rare in the paleontological record prior to MIS 5, then are known *Homo sapiens* remains at Skhul and Qafzeh^5–7^ and Neandertals from Shanidar^8^ and possibly Tabun^1,9,10^. High diversity in Neandertal mortuary practices has been proposed on the basis that chronologically and geographically close groups engaged in varying behaviors, ranging from cannibalism to intentional burial of some of their dead^11^. However, the question of whether or not Neandertals buried their dead has been continually debated since the discovery of the La Chapelle-aux-Saints 1 Neandertal^12–20^. Another question has been whether the proposed burials constitute evidence of symbolic behaviour per se^19^. These discussions parallel the historical debate concerning the physical appearance and abilities of Neandertals, from the brute represented originally by M. Boule^21^ to the opposite tendency^22^. The topic of Neandertal burial is highly controversial, raising questions about the similarity in funerary activity between the two highly encephalized human species, the question of cultural transmission between the two groups and the underlying intention behind the practice (symbolism vs utilitarism). Additionally, criteria for burial recognition have been discussed but remain disputable because some of the criteria proposed as a threshold to prove the presence of Middle Paleolithic burials would not allow some historical burials to be classified as such^12–20,23–26^. Thus, the Neandertal burial debate can sometimes reach beyond the scientific framework to an ideological level. This scientific literature, whether for or against the recognition of Neandertal burials, has limitations that render their conclusions debatable and debated^12–20^. Moreover, the application of the disputed criteria to individual archaeological cases is problematic. La Ferrassie is one of the key sites in this debate due to the extensive and well-preserved fossil record, including several Neandertal partial and complete skeletons.

The *grand abri* (large rock-shelter) de la Ferrassie site (Savignac de Miremont, Dordogne, France; Supplementary Fig. S1) and Shanidar (Iraqi Kurdistan) are the two Middle Paleolithic sites that have yielded the largest collections of partial to complete Neandertal skeletons, interpreted as intentional burials^8,27–33^. La Ferrassie has yielded seven partial or complete Neandertal skeletons: two adults (possibly a male and a female) and five partial skeletons of children of different ages-at-death (Supplementary Fig. S2)^34–37^. Most of these skeletons were found at the beginning of the twentieth century^38–43^. The last skeleton, La Ferrassie 8 (LF8), a partial Neandertal skeleton (cranium, neck and trunk bones, pelvis and four hand phalanges) of a child of around two-years-old, was found in 1970 and 1973 in layer M2, during the penultimate excavation of the site between 1968 and 1973^28,30,31,43^. Only a few non-human skeletal remains and lithics are associated with the skeleton. The discovery and context of this skeleton has generally been regarded as poorly documented^30,36,43,44^, but in fact this deficiency stems from a lack of the necessary processing of the information and materials from La Ferrassie related to the penultimate excavation phase (1968–1973). Indeed, a huge amount of data remained unassessed prior to our current study. The in-depth processing and examination of the material and the insights gained are crucial as other potential instances of Neandertal burial in Europe, including La Chapelle-aux-Saints 1 and the other La Ferrassie specimens, were discovered more than a century ago when data recording of stratigraphy and context was well below modern archaeological standards.

Here we present new data on the LF8 child context from three sources (Supplementary Texts S1-S2). First, the careful reading of the field diaries (housed at the Musée d'Archéologie nationale et Domaine national de Saint-Germain-en-Laye, noted MAN hereafter) and the processing of the spatial data recorded during the excavations at the site performed between 1968 and 1973. Second, we performed multidisciplinary studies on the LF8 skeleton and associated archaeo-paleontological findings. The results include new data on the excavation in 1970 and 1973 of the LF8 Neandertal child, the stratigraphic context and spatial information of the LF8 findings, the first geochronological analysis of the LF8 individual (using ^14^C and OSL dates), Zooarchaeology by Mass Spectrometry (ZooMS) data of the associated unidentified skeletal remains, ancient DNA analysis, and a complete taphonomic analysis of the collection of available human and faunal remains. Third, new excavations and analyses were performed in 2014 at the La Ferrassie site in the location where LF8 was found. In this framework, our objective here is to discuss whether the evidence from LF8 is compatible with an anthropogenic deposition of the corpse and to provide the most parsimonious scenario to explain the origin of the preservation and representation of the LF8 bones. The results from this individual can provide upstream information for the Neandertal burial debate.

## Results

### LF8 Neandertal child: spatial and stratigraphic context

**Figure 1:**
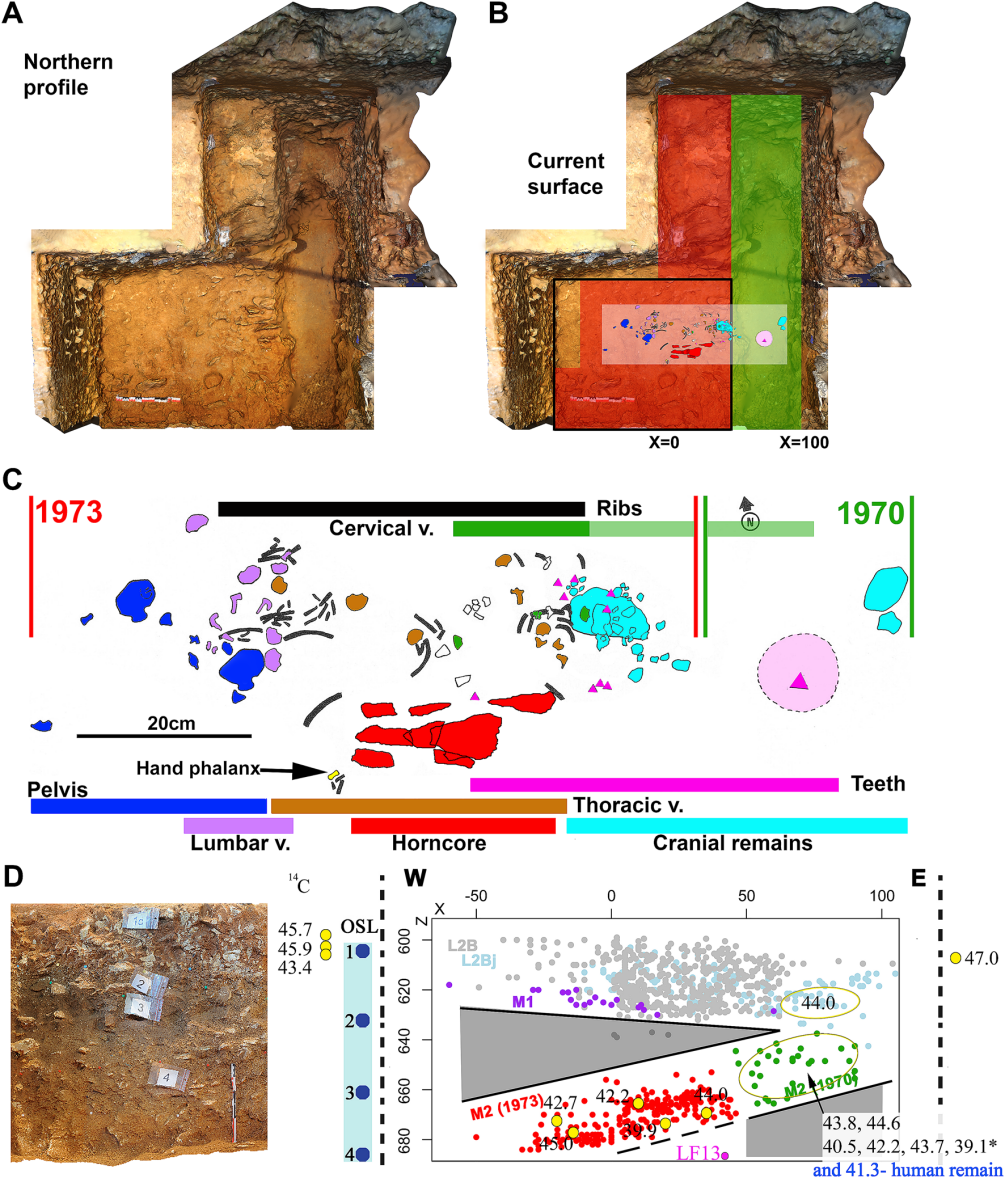
**(A)** Zenithal view of the 3D model of the LF8 excavation area after the 2014 field season, with **(B)** the different excavations performed in the area: the easternmost part (x = 50–100) was excavated in 1970 (green), the westernmost part in 1973 (red) and the area shown in the black square in 2014. This latter area includes a column of approximately 20 × 60 × 80 cm in the westernmost area and the surface of the southern part of the area excavated in 1973. The location of the schematic representation of the LF8 remains is shown. **(C)** Schematic representation of the LF8 remains (modified from^43^ and original documentation), including the position of the different anatomical parts, in which we have added the horncore remains (in red) based on the available spatial information. (**D**) Left: Northern profile of the LF8 area after the 2014 excavation (the numbers of the photos indicate a stratigraphic division in which the uppermost layer would be equivalent to the L2B-L2Bj complex from Delporte). Center*:* The depth where the OSL samples were taken in this profile. Right*:* W–E scatterplot (XZ) of the 1970–1973 findings. Note the west–east downwards inclination of the elements found in layers L2B, L2Bj and M1, the reverse east–west downward inclination of the elements found in layer M2, and the absence of archaeo-paleontological findings between the elements found in layer M2 and those of the overlying layers, as well as the sterile basal part of M2 in the eastwards direction (highlighted in grey). The archaeo-paleontological findings (found in the layer that contained the child in 1970—green dots—and 1973—red dots) extend for c. 120 cm in the E–W orientation, c. 50 cm in the N–S orientation and a regular vertical extension of c. 25 cm, taking into account that the finds, when plotted as a group, show an inclination of 15º to the west. Here we also show the location of the Neandertal tooth fragment (LF13) discovered in 2014 in the dirt layer of the previous excavations. The yellow dots correspond to the samples used for the radiocarbon chronology and the blue dots correspond to the OSL samples. We also summarize here the radiocarbon ages in cal BP (95.4%) and OSL dates (see Table 1 for the detail and Supplementary data, Fig. S10 for a more complete visualization of the position of the dated samples).

The MAN archives provided data to contextualize and precisely locate the area where LF8 was found within the La Ferrassie *Grand abri,* and its position relative to the other Neandertal skeletons (Fig. 1 and Supplementary Fig. S2). LF8 was found in square 1, at the western extent of the 1968–1973 excavations, in the layer named M2 in this area during the excavations. In 1970, a 50 cm wide trench was dug perpendicular to the wall of the rockshelter to get insights into the stratigraphy in this area. This area corresponds to the eastern half of square 1 (Supplementary Text S2). This trench revealed layers K and L (Aurignacian), a “lower Perigordian” layer (i.e., Châtelperronian, named L2bj) and a sedimentary layer (considered basically sterile at that time) that contained seven teeth and two parietal fragments belonging to LF8, which were recognized during the sieving of the sediment. The archaeological level within the thick sedimentary layer was later named M2 in 1973 when the rest of the human remains were found. In 1973, during the final excavation days (August 24th–30th), elements labelled from number 295 (the first human bone identified) up to 538 were collected. More recently, forty-seven human remains, including cranial remains, mandibular fragments, vertebral and costal remains and two hand phalanges were identified among the indeterminate paleontological remains, mainly from the 1970, but also 1973 field seasons^31^, which add significantly to the completeness of the skeleton. The attribution of these remains to LF8 was based on their consistency with the existing remains in terms of size and age-at-death, the lack of anatomical duplication, the direct refitting of bone elements, and their spatial location within the La Ferrassie stratigraphy^31^. The field notebooks provide detailed information on the spatial distribution of the 1968–1973 findings (Supplementary data 1), including the density of the findings from layer M2, and their general spatial organization and orientation, relative to the other layers. The LF8 remains were found associated with faunal remains and lithics. It should be noted that there is no clear difference in terms of technology, state of preservation, or type of lithics between the two limited samples of lithic elements coming from the layers M2 and L2Bj in square 1 but sample size is small for both assemblages, rendering comparisons difficult.

The information about the geological and stratigraphic context of LF8 deriving from the excavations in 1968–1973 is very limited. The place where LF8 was found was an area of low sediment deposition compared to the westernmost part of the site^45^. Moreover, a general north-east inclination of the archaeological layers is observed in this sector as illustrated by the stratigraphic sketches made during these excavations^46^. In addition, a similar pattern is visible when we plot the findings of Delporte’s excavations for all the layers, including the L2B-L2Bj complex, located just above the layer M2 (Supplementary Fig. S6 and S10). The level M2, where LF8 was found, is archaeo-paleontologically sterile except in the limited area where this individual was found. This was noted by Delporte in his diaries for squares 1 and 2 and we have been able to confirm it (Supplementary Text S2). The features of the loose sediment associated with the LF8 remains are consistent with that found at the site close to the location where LF8 was found (Supplementary Text S2).

Interestingly, the LF8 remains and associated archaeo-paleontological findings show a relatively strong inclination to the west, in contrast to the general dip of M2 or other strata in this sector (Fig. 1), which indicates that they do not follow the natural dip of the stratum that contains it. The LF8 head remains were found to the East while the pelvic elements were found to the West and at a greater depth (Fig. 1). The LF8 remains are scattered in the west–east axis over an area of 94 cm, while the dispersion of the LF8 elements in the north–south direction is low (~ 20 cm), and just one hand phalanx was found 10 cm to the south. Finally, the cranial parts are topographically higher than the pelvis (~ 30 cm).

### 2014 excavations

In August 2014, we went back to the field in order to gain additional insights in the LF8 sector. We were able to confirm a great density in archaeological findings at the same depth as Delporte’s L2B-L2Bj complex, in adjacent squares (Supplementary Fig. S10). Moreover, in the area where we excavated, i.e., 50 cm to the west of square 1, the geological layer at the same depth where LF8 was discovered was basically sterile. It was also possible to directly confirm that the area where LF8 was found had been completely excavated. As a result, the new field season did not provide additional information on the LF8 context but confirmed previous observations: the general inclination of the layers above LF8, and the discrepancy between the reconstructed inclination of the LF8 assemblage within M2 (based on the spatial information recorded by Delporte) relative to the sedimentary layer that contains it and to the contact with the L2Bj layer.

We found a new Neandertal fossil, a molar fragment of an individual older than 5 years, at the beginning of our excavation, within the dirt layer on the surface of Square 1, deeper than the archaeological assemblage associated with LF8 (Supplementary SI text 2, Fig. S13). This was the only archaeological finding on the entire excavation surface (100 cm × 50 cm) of the LF8 area in the approximatively 10 cm depth that we excavated. We hypothesize that this element likely fell from one of the surrounding profiles just before the excavation of the 1973 field season ended. The anatomical features observed on this fragment are consistent with a Neandertal lower left molar (Supplementary Text S2). Following our other recent human fossil discoveries in the La Ferrassie collections ^47^, we name this specimen LF13.

### Geochronological context

In order to obtain a chronological framework for the stratigraphic sequence preserved in the LF8 sector, four sediment samples were collected for luminescence dating in the stratigraphic section exposed to the north of square 1 during the 2014 excavations (Fig. 1). The first of these dating samples was taken from the rich archaeological level corresponding to Delporte’s levels L2B and L2Bj, yielding a result of 43 ± 6 ka using the minimum dose model (MDM^48^) or 54 ± 3 using the Internal External Uncertainty model (IEU^49^) (Supplementary Text S2 and Table S1). The MDM results are consistent with five ^14^C dates of bone samples from the same layer excavated in both 2014 (between 49.1–43.3 and 44.2–42.5 ka cal BP, 95.4%) and 1970 (44.6–43.3 ka cal BP 95%) (Table 1; Supplementary Table S1). The other three (deeper) OSL dates yielded older results. The sample at the same depth where LF8 was found yielded an age of 60 ± 7 ka (MDM) or 66 ± 4 ka (IEU) (Supplementary Table S6). Ten faunal remains from Delporte’s excavations associated with LF8 yielded ^14^C dates ranging between 45.5–39.5 ka cal BP (95.4%) (Table 1). In fact, the dates associated with the LF8 child are either of similar age or younger than those from the overlying level, the L2B-L2Bj complex (Fig. 1; Supplementary Fig. S10). Finally, in addition to the ten faunal remains associated with LF8, one element classified as a hominin through ZooMS yielded an age of 41.7–40.8 ka cal BP (95.4%). Mitochondrial DNA of this directly dated hominin groups with Neandertals (Supplementary Table S5).

**Table 1:** AMS radiocarbon dating of 17 samples from La Ferrassie.

MPI-code	Field season	Layer	% Coll	C:N	^14^C age (BP)	1σ err	Cal BP 95.4%	
							From	To
R-EVA-1386^a^	2014	L2B-L2Bj	2.9	3.2	44,380	980	49,090	44,950
R-EVA-1374	2014	L2B-L2Bj	4.1	3.2	43,010	830	47,390	44,390
R-EVA-1387	2014	L2B-L2Bj	3.2	3.2	42,870	800	47,150	44,320
R-EVA-1377	2014	L2B-L2Bj	2.5	3.3	39,880	570	44,230	42,550
R-EVA-1614	1970	L2Bj	3.4	3.2	41,070	320	44,580	43,280
R-EVA-1607	1970	AC	6.6	3.2	40,720	310	44,380	43,130
R-EVA-1608	1970	AC	6.5	3.2	41,820	260	45,060	44,240
R-EVA-1609	1973	AC	3.5	3.3	37,670	170	42,330	41,970
R-EVA-1610	1973	AC	6.6	3.3	41,260	350	44,740	43,340
R-EVA-1611	1973	AC	9.6	3.3	34,770	170	40,430	39,500
R-EVA-1612	1973	AC	1.6	3.3	39,230	190	42,930	42,510
R-EVA-1613	1973	AC	7.5	3.2	42,350	290	45,500	44,510
R-EVA-3336^b^	1970	AC	5.7	3.3	36,170	220	41,710	40,820
R-EVA-3337	1970	AC	8.8	3.2	35,400	200	41,020	40,020
R-EVA-3338	1970	AC	7.5	3.3	37,750	260	42,400	41,940
R-EVA-3339	1970	AC	7.7	3.3	40,580	360	44,340	43,020
R-EVA-3340^c^	1970	AC	3.6	3.4	34,030	180	39,650	38,590

The amount of collagen extracted (%Coll) and C:N ratios refer to the > 30 kDa fraction. The ages are rounded to the nearest 10 years.

Elements found in 2014 are attributed to the L2B-L2Bj complex because it was not possible to directly relate the excavated area (at the same elevation but located to the West or to the East) to the layers excavated in 1970.

Ages have been calibrated using OxCal 4.4 ^61^ using the international calibration curve IntCal 20 ^62^.

AC = associated to the child (LF8), those elements come from the bag filled in 1970 with the objects founds in the layer M2 in the eastern part of square 1 (in green on Fig. 1) and from the box filled in 1973 during the excavation of the hominin remains in the western part of square 1 (in red on Fig. 1).

^a^May extend out of range, but this bone is located to the east of the area where LF8 was found. We cannot ascertain its provenience from the L2Bj layer that was located above the archeo-paleontological layer that contained the child.

^b^Identified by ZooMS as a human remain, and as a Neandertal using mitochondrial DNA.

^c^This bone was collected in 1970 while the area was excavated more rapidly than in 1973. Its deamidation value is different from the other elements analysed by ZooMS (Supplementary Fig. S15), indicating a potentially different post-mortem history.

For a more comprehensive version of this table see Supplementary Table S1.

### Zooarchaeology by mass spectrometry (ZooMS)

Most (*n* = 13) of the 17 taxonomically unidentified bone specimens associated with the LF8 skeleton analysed by ZooMS were identified as *Bos/Bison*, which is consistent with the taxonomical assessment of the macro-faunal remains associated to LF8 (Supplementary Table S8). From the remaining four, two could not be identified taxonomically, one belonged to an ursid (*Ursus* sp.), and one was a hominin specimen. Additionally, 14 bone remains from the layer overlying the LF8 deposit (L2Bj) were analysed to provide a comparative context for the deamidation data of the specimens associated with LF8. The deamidation data of the bones associated to the LF8 remains are similar to those from L2Bj, except for the ursid specimen that shows a different signature (Supplementary Fig. S15). This ursid specimen also yielded a radiocarbon age of 39.0–38.6 ka cal BP (95.4%), the youngest of the radiocarbon ages on bone material associated to the LF8 child. Both the younger chronology and the different deamidation value suggest a different post-depositional history than the other ZooMS-analysed samples from this area. As a result, this bear specimen indicates that the bones spatially associated with LF8 contain material that is either from different chronological and/or diagenetic histories. However, this ursid specimen is the exception, as the rest of the analysed material shows similarity in deamidation data, as well as great homogeneity in taphonomic indicators and ^14^C dates. The presence of this specimen might therefore reasonably be explained by an excavation or sampling error in 1970 (Supplementary, Text, Figs. S5, S9, S12).

### Taphonomy of the LF8 skeleton and associated remains

A taxonomical and taphonomic study was performed on a total of 4609 paleontological remains, including the 191 elements belonging to the LF8 child. This exhaustive analysis includes all the recovered skeletal material associated with this important discovery. Most (NR = 3,492) were indeterminate, small (< 25 mm), burnt bone fragments. Only 22 of the 127 anatomically identifiable faunal remains were taxonomically identifiable and corresponded to cranial remains and diaphyseal remains of *Bos/Bison* (NR = 9), *Equus ferus* (NR = 7) and cervids (NR = 6), including some fetal remains (Supplementary Tables S8, S9, S10). The taphonomic and taxonomic spectrum of the faunal sample is entirely consistent with the results from the new excavations carried out at La Ferrassie. Among the faunal remains associated with LF8, the largest corresponds to a bison (*Bison* sp.) horncore that was found in situ in 1973 in more than 25 fragments that were found close to each other.

The taphonomic results (Supplementary Text S1) point towards the presence of two different groups: the human remains and the faunal remains. The human pieces are complete (or almost), were recovered in close spatial association (for some anatomical regions, possibly anatomically connected), and represent a single individual. Animal remains consist of scattered fragments belonging to various individuals that show carnivore marks, cut-marks, green-bone fractures and fire alterations. None of these alterations have been observed on the human remains. Conversely, taphonomic alterations specific to the burial conditions are similar for both samples. Thus, these data highlight two different biostratinomic (i.e., prior-to-burial) scenarios, but similar burial conditions (same sedimentary context).

## Discussion

The La Ferrassie 8 Neandertal child constitutes the last of the Neandertal skeletons found at the La Ferrassie rock-shelter (in 1970 and 1973). The published contextual data for this individual has been limited to a drawing of the scattering of the bones published in monographs^28,36,43^. The analysis of the contextual data together with the finding of new remains^31^, the taxonomic and taphonomic analysis of the whole collection, and a robust chronological context provide the tools for the interpretation of the archaeo-paleontological context of this child for the first time. After the evaluation of the data, we propose what we consider the most parsimonious scenario to explain this specific case.

The LF8 child shows a W-E orientation, with the head (to the E) located at a higher elevation than the pelvic bones (to the W). This inclination does not follow the natural dip of the level where this child was found (M2) or of the overlying archaeological layers (the L2B-L2Bj complex). The faunal remains associated with LF8 directly dated with ^14^C are either of the same age or younger than the ^14^C results for the bones found in the overlying layer. The ^14^C dates obtained in samples from the L2B-L2Bj complex are similar to the OSL date obtained at the same elevation. In contrast, the ^14^C dates of the remains associated with LF8 are younger than the OSL date of the geological layer at the same depth where LF8 was found. Finally, the ^14^C age of one hominin bone fragment identified by ZooMS and genetically linked to Neandertal using mitochondrial DNA is among the youngest ages of all the analysed elements. The most parsimonious explanation is that this hominin bone also belongs to LF8. The obtained age (ETH-99102: 36,170 ± 220 ^14^C BP) is consistent with the most recent chronology for the Châtelperronian at La Ferrassie ^50^ and is the same age as the directly dated Neandertal remains from Saint-Césaire (OxA-18099: 36,200 ± 750 ^14^C BP) and Arcy-sur-Cure (MAMS-25149: 36,840 ± 660 ^14^C BP), which have been associated with the same techno-complex^51,52^ (but see^53^). Moreover, these three dates, together with those from Goyet^11^, constitute the most recent directly dated Neandertal remains. This observation does not engage us in the current debate concerning the Châtelperronian artisan. We need additional data and new sites to definitively assess the role of Neandertals in the development of this techno-complex and the possible influence of the first *Homo sapiens*. In fact, the recent finding of the oldest *Homo sapiens* with jewellery in central Europe^54^ has fueled the debate regarding the possible acculturation of Neandertals, which is beyond the scope of the current study.

Regarding the human remains, the absence of carnivore marks, the low degree of spatial disturbance, fragmentation, and weathering suggest that they were rapidly covered by sediment. The LF8 remains are very well preserved, despite belonging to a child which are generally more delicate. Additionally, there are clear differences in the preservation of the human remains, on the one hand, and the associated faunal remains on the other. These two groups have different post-mortem and taphonomic histories. It also should be noted that based on both Delporte’s reports and our own excavations the M2 layer, where LF8 was found, is sterile except in the zone where LF8 was found (Fig. 1).

Should the corpse have been laid on the floor (whether intentionally or otherwise), in the absence of erosive processes a similar inclination of the human remains and the rest of the strata would be expected. Moreover, we could expect a wider spread of the archaeological remains. Currently, we cannot find any natural (i.e. non-anthropic) process that could explain the presence of the child and associated elements within a sterile layer with an inclination that does not follow the geological inclination of the stratum. In this case, we propose that the body of the LF8 child was laid in a pit dug into the sterile sediment. The similarity of the sediment filling this hypothetical pit to the surrounding sediments, together with post-depositional processes present at the site^28,55^, made it impossible to ascertain the limits of the pit during the excavation process^56^. However, the spatial distribution of the objects associated with LF8 inside the sterile sediment offers an archaeological clue to the presence and extent of the pit (Fig. 1).

The proposed scenario is consistent with the limited surface in which archaeo-paleontological remains were found within the M2 layer (essentially, only LF8 and the associated remains). This scenario would also explain the apparent stratigraphic problems with the chronology, i.e., the ^14^C dates of the elements associated with the LF8 individual are either similar in age or younger than the dates from the layers overlying the LF8 individual. In addition, the directly dated hominin fragment has yielded one of the youngest ^14^C dates of the dated assemblage. Moreover, it would also explain the apparent discrepancy between the OSL and ^14^C dates in the M2 level, which indicates that the paleontological elements are younger than the time of deposition of the sediment that surrounds them. This discrepancy is not seen in the L2B-L2Bj layer complex overlying the LF8 skeleton, where the OSL ages (minimum dose mode) agree with the ^14^C dates. Agreement between the OSL ages and ^14^C dates was also observed in other sectors of the La Ferrassie site, demonstrating consistency between the two methods for determining the chronology of the site (level 6, Châtelperronian^50^). The 

deamidation data of the bones from L2Bj are very similar to those of the hominin specimen and the fauna remains associated with LF8, indicating similar diagenetic conditions, with the exception of just one specimen (Supplementary Text S2; Fig. S15). Moreover, the bones from L2Bj have similar ^14^C dates to the Neandertal specimen and the fauna remains associated with LF8. There is no clear difference in terms of technology, state of preservation and constitution between the two limited collections of lithics coming from layers M2 and L2B-L2Bj in square 1.

However, the LF8 remains extend for c. 120 cm, which is longer than would be expected for the head and trunk of a two-year old Neandertal child. Based on the spatial information, there is a high degree of anatomical consistency in the distribution of the LF8 remains. However, the poor photographic documentation of the 1973 excavation and the rapid excavation progression mean that we cannot ascertain whether some anatomical elements of the LF8 child were found in similar anatomical positions observed for other Neandertal children, such as Amud 7 or Dederiyeh 1^14,56,57^. It is interesting to note that the Neandertal record comprises several well preserved immature individuals whose state of preservation, taphonomic condition and geochronological situation within their respective sites is unique compared to other hominin species.

There are some difficulties in the interpretation of LF8 that need to be discussed. The anatomical representation of LF8 is restricted to the cranium, thorax, pelvis, and four hand phalanges^31^. The reason for the lack of upper and lower limbs (excepting hand phalanges) remains unsolved. It should be noted that only the adult Neandertal skeletons from La Ferrassie are complete. All five immature skeletons are partial^34–36^, which could result from different reasons (differential burial, taphonomic processes, differential care in the excavation process). The recent excavations of the LF8 sector did not reveal the missing anatomical parts of LF8. Neither did the analysis of the museum collections^31^, although these did provide a better representation of the anatomical regions already known. We propose several possibilities to explain this paradox. Firstly, the child was complete at burial, and the subsequent disappearance was the result of geological post-depositional processes and/or the excavation conditions and/or museum curation conditions. Indeed, the southern and western borders of the area where LF8 was found were possibly exposed during the Capitan and Peyrony excavations. As a result, the missing bones could have been lost at any moment between the 1920’s and 1968. Alternatively, the anatomical distribution of LF8 could be the result of secondary purposeful burial, or a partial primary burial^27^. In any case, geological processes are likely (at least partially) responsible for the more scattered distribution of the human remains in the W–E direction. Based on the available evidence, these should be regarded as alternative explanatory hypotheses, as we cannot firmly conclude on this topic. However, the absence of upper and lower limbs, which are more robust, if not fragmented, than postcranial axial skeletal elements (e.g., vertebrae) which in this case are excellently preserved given their fragility, does not affect the other scientific evidence described in this study.

The new information about the LF8 individual can be compared to the other individuals from La Ferrassie in order to see whether they follow the same pattern. First, the E–W orientation of the LF8 skeleton is also observed for both adult individuals. In both adult cases, the head is also topographically higher than the pelvis. These two skeletons were located with a separation of around 50 cm between their heads, with LF2 being located more westward than LF1. Additionally, D. Peyrony, H. Breuil and M. Boule observed small packets of yellow sand mixed with the Mousterian sediments associated with both adult individuals^58^. These packets were not observed elsewhere in the Mousterian layers, but correspond to sediment from the underlying layers. This observation has been interpreted as the effect of intentional funerary pits that removed sediment from the underlying level and mixed it with the sediment filling the pit^58^. The presence of a triangular-shaped pit covered with a triangular-shaped stone was also proposed for the partial LF6 infant^59^. A recent taphonomic analysis of the LF1 adult individual indicates that the fracture pattern and the absence of surface modification on this skeleton, together with its overall completeness is also consistent with an intentional burial, as proposed by previous studies^32^. Future complementary approaches should compare our multidisciplinary information with similar data from other sites that yielded Neandertal immature individuals, such as Dederiyeh or Amud.

Additional studies of the other specimens from La Ferrassie should aim to provide the same level of detail now available for LF8 and LF1^31,32^. This multidisciplinary approach should also be extended to other Neandertal skeletons and sites, to provide robust data that will allow us to contextualize information at both the site and regional scale. This is necessary to seriously address historically debated issues such as the relationship of Neandertals with the Châtelperronian industry and the chronological and geographical extent of funerary behaviours^60^, as well as providing a more precise chronology for the last Neandertals.

## Conclusions

The combined anthropological, spatial, geochronological, taphonomic and biomolecular data analysed here suggest that a burial is the most parsimonious explanation for LF8. Our results show that LF8 is intrusive within an older (and archaeologically sterile) sedimentary layer. We propose that Neandertals intentionally dug a pit in sterile sediments in which the LF8 child was laid. The skeleton was laid in an E (head)–W (pelvis) orientation (as are all the Neandertal skeletons found in the site for which we have information), with the head higher than the rest of the skeleton (as can be deduced for LF1 and LF2 from the information in Boule’s archive at the IPH). Some lithics and faunal remains that were on the ground at the time of burial could have fallen into the pit while it was being prepared or filled. The sterile sediment removed previously would have been used to fill the pit and cover the child. This would explain why the faunal remains associated with LF8 have a similar (or younger, in the case of the directly dated hominin fragment) ^14^C age compared to the faunal remains found in the overlying layer. These dates are also in agreement with the OSL age of the overlying layer, whereas the OSL age of level M2 at the elevation of LF8 is much older. The direct date obtained on a Neandertal bone fragment, identified by ZooMS and confirmed through ancient DNA, is the first direct date from a human from La Ferrassie, yielding an age contemporaneous to the directly dated Neandertals from Saint-Césaire in Poitou–Charentes and Arcy-sur-Cure Grotte du Renne in Burgundy France. These new results provide important insights for the discussion about the chronology of the disappearance of the Neandertals, and the behavioural capacity, including cultural and symbolic expression, of these humans.

## Material and methods

A complete re-inventory of the anthropological collections of the Muséum national d’Histoire naturelle has been facilitated by their transfer to the Jardin des Plantes from the Musée de l’Homme (Paris, France) due to renovations. Several boxes contained elements from the site of La Ferrassie (Dordogne, France) where new human fragments were identified. Some elements fit with the LF1 and LF2 skeletons. Another box contained elements from the excavations of Delporte in 1973 that were related to the discovery of LF8. To complement the information available in this box, including the new human remains, we visited the collections and archives of the excavations led by Delporte in La Ferrassie from 1968 to 1973 at the Musée d'Archéologie nationale et Domaine national de Saint-Germain-en-Laye (noted MAN). This provided a better understanding of the context of the 1968–1973 excavations and their results (Supplementary Text S1). Graphic documentation, including photographs of square 1 from the final days of the 1973 field season were useful to visualize and understand the process of the excavations. Notebooks with information about the labelling and 3D coordinates of each element were crucial to reconstruct the spatial distribution of the Mousterian layer and the LF8 elements (Supplementary Text S2, supplementary data 1 for the raw data used, and Figs. S6 and S10). The notebooks for square 1 (field seasons 1970, 1972 and 1973) are entirely reproduced (SI Supplementary data 1). Among the boxes in the MNHN and in the MAN, several fragments of a partial bison horncore that perfectly refit together were discovered (Supplementary Text S2) as well as numerous new human remains of LF8 (Supplementary Text S2). The attribution of the new human remains to LF8 is based on their consistency with the existing remains in terms of size and age-at-death, the lack of anatomical duplication, the direct refitting of bone elements, their location in the deposit, and the taphonomic analyses (following the methodology of Rendu and colleagues^16^) of the entire fossil bone collection, including the horn and other faunal elements and the complete hominin collections. The archives of Marcelin Boule (housed at the MNHN and at the Institut de Paléontologie Humaine, Paris, France) were visited in order to search for information regarding the La Ferrassie site. In order to provide a more thorough evaluation of the original LF8 context, we present new data on the archaeo-stratigraphic context of the LF8 Neandertal child including: spatial data of the LF8 fossils and associated finds, taphonomic analysis of LF8 and the associated faunal remains, stratigraphic information regarding the findings from the LF8 sector (both from Delporte’s excavation and our own excavation in 2014), new luminescence and ^14^C ages and ZooMS data of some indeterminate fossil remains associated to LF8. The methodologies follow classic protocols and are detailed elsewhere (Supplementary Text S1). The methods used for the retrieval and analysis of ancient mitochondrial DNA are described in detail in Supplementary Text S1.

## Supplementary information

Supplementary Information 1.

Supplementary Information 2.
