# Supplementary material for: Pluridisciplinary evidence for burial for the La Ferrassie 8 Neandertal child
Source: Sci Rep. 2020 Dec 9;10:21230. doi: 10.1038/s41598-020-77611-z (PMC7725784; doi:10.1038/s41598-020-77611-z)
Supplement: Supplementary file 1 — Supplementary Information 1. [file 41598_2020_77611_MOESM1_ESM.pdf]

SUPPLEMENTARY INFORMATION FOR

Pluridisciplinary evidence for burial for the La Ferrassie 8 Neandertal child

Antoine Balzeau, Alain Turq, Sahra Talamo, Camille Daujeard, Guillaume Guérin, Frido Welker, Isabelle Crevecoeur, Helen Fewlass, Jean-Jacques Hublin, Christelle Lahaye, Bruno Maureille, Matthias Meyer, Catherine Schwab, Asier Gómez-Olivencia

Antoine Balzeau
antoine.balzeau@mnhn.fr

Asier Gómez-Olivencia
asier.gomezo@ehu.eus

This PDF file includes:

SUPPORTING TEXTS
- Text S1. Material and Methods
- Text S2. Results

SUPPORTING FIGURES
- Figs. S1 to S18

SUPPORTING TABLES
- Tables S1 to S14

Supplementary references (1-77)

Other supplementary materials for this manuscript include the following:

Supplementary data 1 (notebooks for square 1)

SI Appendix Text S1. Material and Methods

General context

This research is part of an ongoing effort to restudy the La Ferrassie (hereafter LF; *SI Appendix*, Fig. S1) hominin collections that are housed in the Muséum national d'Histoire naturelle in Paris (hereafter MNHN) and their context. In this context, both work at the collections in different institutions and work on the original site was performed.

This particular research on the La Ferrassie 8 individual started on 20th March 2013 with the revision of a box with miscellaneous material from the collections of MNHN. Henri Delporte gave this box (nicknamed "the brown box") to Jean-Louis Heim and represents part of the materials mainly (but not exclusively) related to the discovery of La Ferrassie 8 (hereafter LF8) Neandertal child (1, 2). The LF8 elements recognized and studied by Jean-Louis Heim were removed from this box and are now stored in their own box (number 48) in the anthropological collection of the MNHN at Musée de l'Homme. The "brown box" contained: sediment samples; paleontological remains, among which there were previously unrecognized human remains; lithic finds and two maps plotting the LF8 remains that were likely used as a basis for the maps published and used subsequently (1, 3; see below). All these elements were collected in 1973 in square 1 within a few days and on a small surface (see below). One additional object in the box is one isolated adult tooth in its own individual tube.

The human remains identified in 2013 from this box can be divided into two groups. The first comprises an adult human tooth, found in 1970, and recognized as such in the excavation (as we have learnt from the excavation report), which was not included in La Ferrassie monographs by Heim (1, 4, 5) and was forgotten in the box. This tooth represents an additional adult individual in La Ferrassie and is described thoroughly elsewhere (6). The excavation reports indicate that it was found in the layer labelled L2Bj, located 30 cm above all the archaeo-paleontological material associated to LF8 (Supplementary data 1). The second group includes several previously unrecognized human remains from the 1973 excavation that were mixed with the faunal remains and that can be related to the LF8 individual (7). This material is clearly identified as originating from the 1973 excavations and the layer M2.

Part of the interest of these new LF8 fossils was that they opened the possibility that additional fossil remains could not have been recognized during the LF excavations. Thus two visits to the Musée d'Archéologie nationale et Domaine national (previously known as the Musée des Antiquités Nationales, in France) de Saint-Germain-en-Laye (MAN) were done. The MAN curates the archaeo-paleontological material found during Delporte's excavations except the content of the "brown box" and the LF8 human remains. The objectives of these two visits were: 1) to revise the collections from Delporte´s excavations of LF; and 2) to find documentation of these excavations.

Regarding the first objective, particular emphasis was placed on those bags and boxes related to or adjacent to square 1 (i.e., squares 1 and 2) and the Mousterian levels from the excavations from 1968 to 1973. The visits to the MAN resulted in the identification of additional human fossils belonging to LF8 from both the 1970 and 1973 field seasons. The human remains were mostly found in a unique bag on 21st March 2013. This bag was directly filled during the 1970 fieldwork (as it was clearly stated on the bag), came from the Mousterian layer dug in square 1. Regarding the second objective, we studied the archives of the excavation (noted Delporte archives hereafter) that included: notebooks and/or pages detailing the list of the archaeo-paleontological findings discovered organized by square and field season; some administrative information including letters and excavations reports; some notes dating from the excavations; stratigraphic drawings, maps and photographs including other versions of the map of LF 8 which certainly pre-date the versions found at the MNHN. Copies and/or photographs of all the information pertaining to the Mousterian layers of square 1 and adjacent areas were taken and are presented here.

The Delporte archives provide the exact location and contextualization of the area where LF8 was found. This allows to locate LF8 relative to the other Neandertal skeletons (*SI Appendix*, Fig. S2) and relative to the current state of preservation of the site (*SI Appendix*, Fig. S3). LF8 was found in square 1, at the western extent of the 1968-1973 excavations (*SI Appendix*, Fig. S4), within the Mousterian layer named M2 in this area. In 1970, a small trench, 50 cm in width, was dug perpendicular to the wall of the *abri*. This area corresponds to the eastern half of square 1. Human remains belonging to LF8, comprising seven teeth and two parietal fragments, were found in this trench but were only recognized later, after sieving. In 1973, the western half of square 1 was dug (*SI Appendix*, Fig. S5) during the last week of the field season, between the 24th and the 30th of August, and elements labelled from number 295 (the first human bone identified) up to 538 were collected.

Even the people who were directly involved in the research of the archaeo-paleontological materials found in the 1968-1973 excavations were not aware of a large part of the information recorded during the excavation. This is reflected in comments about the lack of data on the context of the specimen (1, 8, 9). This explains the accepted view of the discovery of LF8 as having been poorly documented ("*En conclusion, on peut regretter le manque de détails précis – surtout pour une fouille aussi récente – et d'informations sur le dégagement des restes humains. Nous ne connaissons pas de photographie de ces derniers en place sur le terrain, ni même leur emplacement réel par rapport au carroyage du gisement*" (In conclusion, we regret the lack of specific details - especially for such a recent excavation - and information on the release discovery of the human remains. We do not know of any photographs of these [the human fossils] in the field, or even their actual location relative to the grid of the field set up for the excavation) (9). Moreover, Laville (3) commented that the precise location of LF8 within the site was unknown. In contrast, the MAN houses a rich documentation concerning these excavations. This information permits us to document the timing of the excavations and of the findings as well as the spatial distribution of the Mousterian layers and the objects it contains, including the remains of LF8 (*SI Appendix*, Figs. S6, S7, S8 and S9).

In parallel, the revision of the faunal remains associated with LF8 in the box housed at the MNHN lead to the identification of several fragments that belonged to a bison horn-core on the 14th of June 2013. The subsequent reconstruction and information from the archives of the Delporte excavations allowed us to determine that the fragments that compose this horn were found parallel to the LF8 remains and in an E-W orientation. Finally, in order to complete the study of the new LF8 remains, a revision of the original collection of LF8 was carried out which resulted in some anatomical and taxonomic re-identifications (7).

In order to provide a more thorough evaluation of the original LF8 context, we present new data on the archaeostratigraphical context of LF8 Neandertal child including:

- spatial data of the LF8 fossils and associated finds

-taphonomic study of the LF8 and associated faunal remains

-stratigraphic information regarding the findings in the LF8 sector, both from Delporte's excavation as well as from our own excavation in 2014

-new luminescence and ^{14}C datings

-ZooMS and ancient DNA data of some indeterminate fossil remains associated to LF8 (*SI Appendix*, Fig. S10)

<u>Revision of the collections</u>

The Delporte archives, housed at the MAN, allowed a better understanding of the context of the 1968-1973 excavations and their results. Notebooks with information about the label and 3D coordinates of each element were crucial to redraw the spatial distribution of the Mousterian layer

and the LF8 elements. The notebooks for square 1 (1970, 1972 and 1973 field seasons) have been scanned and are available in Supplementary Data 1. These notebooks provide 3D coordinates of each finding and these coordinates were transferred to a spreadsheet in order to facilitate 2D projections of this data.

Graphic documentation, including photographs of square 1 in which LF8 individual was found from the final days of the 1973 field season were useful to visualize and understand the excavation process.

Laser scanning of the horn-core was performed with a NextEngine portable scanner in order to virtually reconstruct this important object and preserve a digital copy of it.

<u>2014 excavations at La Ferrassie 8 sector</u>

Starting in 2010, new excavations were performed at the LF site, under the direction of A. Turq in the context of a larger scientific project. These excavations aimed to obtain better contextual information for the LF1 and LF2 adult Neandertal skeletons including to date of the archaeological layers (10, 11). Due to the information gathered from the Delporte archives in 2013, an additional excavation of the LF sector where LF8 was found was planned for 2014 by some of us (AB, IC, AGO). It was hypothesized that due to the abrupt end of the 1973 field season, part of the square west to square 1 was not completely excavated and thus, some bones could remain *in situ*. In this context, the sector where LF8 was found was reopened in July 2014 and excavated in August 2014 with three objectives: 1) to better understand the stratigraphy of the site, especially the LF8 sector compared to the westernmost part of the site; 2) to test our hypothesis regarding the exact location of the LF8 individual; and 3) gather additional contextual information of the LF8 individual, as well as to find the sediment from which the child was originating and to identify new associated findings, including lithic and faunal remains as well as the missing anatomical elements of the child.

After the Delporte excavations, part of the remaining sediments was protected with cloth, and the area was bordered with a low wall and the space in-between covered with small stones. In order to access the *in situ* sediments of the LF8 sector it was first necessary to remove this recent small stone infilling. The removal of this sediment revealed the geometry of the site after Delporte's excavations. In fact, thanks to the photographs from the Delporte archive it was possible to locate the reference system used by Delporte in his excavation.

The 2014 excavation in the LF8 sector (*SI Appendix*, Figs. S11, S12) took place sequentially: first, an area measuring 50 x 100 cm located west and south to the area dug during the Delporte's campaign was excavated; second, a small column of sediment preserved in the north-western corner of square 1 was excavated; finally, the surface of the square 1 where LF8 was found was cleaned and further excavated. All the findings were recorded using the standard recording system used in the western part of the site. Additionally, the XYZ coordinates of the findings of the LF8 area from Delporte's excavation were transformed into our coordinate system. Photographs of the excavation process were taken and, using photogrammetry, 3D models were derived.

<u>Sampling strategy for luminescence dating, radiocarbon dating and ZooMS analysis</u>

LF8 was excavated in the 70's, we therefore cannot do any sampling within its original archeological context. For conservation reasons, we chose not to use any identified human bone for direct dating. Indeed, the preserved bones are very small and fragile. Moreover, the paleoanthropological record for such delicate bones of Neandertal children is so scarce that we decided not to sample any identified human remain. As a result, we had to elaborate a strategy with different methodologies in order to collect as many information as possible and to chronologically contextualize this specimen.

Four samples for luminescence dating were collected along the stratigraphy exposed along the northern profile of square 1 during the 2014 excavations (*SI Appendix*, Fig. S10). The purpose was to obtain a chronology for the sedimentary sequence preserved in the LF8 sector in order to allow comparisons with other sectors of the sites but also with results obtained with other methodologies on samples more closely related to LF8. Indeed, the samples for luminescence dating cannot be directly related to the sedimentary infilling where LF8 was found as it was completely excavated in the 1970s.

Samples for radiocarbon dating and ZooMS analysis were selected among the material collected in 1970 and 1973 field seasons and for which we have information on their spatial context. Two specimens from the 1970 excavations were selected, recovered from a bag filled with objects collected in the eastern half of square 1 of the Mousterian layer (1607 and 1608; *SI Appendix*, Table S1 and Fig. S10). Those samples come from a unique small bag, stored at the MAN. This bag was filled only on August 20th-21st. On those days, the human remains had not been recognised yet. Only a small part of the archaeological objects, mostly lithics, were at that time labelled and their coordinates registered. As a result, we do not have the precise localisation of those two samples but we know that they come from a very limited surface (30cm × 30cm) and that they were spatially and archaeologically associated to LF8. Five faunal remains (labelled 1609, 1610, 1611, 1612, 1613, *SI Appendix*, Table S1 and Fig. S10), found in 1973 close to the LF8 skeleton were also selected to provide the most precise information about this Neandertal specimen. These faunal remains were in the brown box stored at the MNHN. We have detailed information for these specimens in the notebook found in the MAN. In 1973, the excavations were done with much more scrutiny, and the majority of the objects were coordinated and labelled.

We have also selected one sample collected in 1970, also in the eastern half of square 1, but in the overlying layer L2Bj (1614). This specimen was in a bag in the MAN together with findings from the same layer. Finally, four samples from the 2014 excavations (*SI Appendix*, Fig. S12) were selected for ^{14}C dating. Three of these samples come from the westernmost column that was excavated, and the level where they were found corresponds in terms of elevation to Delporte's L2B-L2Bj level complex, *i.e.* the layer overlying the layer where LF8 was found. Similarly, the fourth sample was found on the easternmost border of the area excavated in the 70s. These samples were found laterally and above the location of LF8 and were selected to allow for a chronological contextualization of the borders of the excavated area.

In parallel, 17 fragments, non-identifiable at the anatomical and taxonomical levels but which, *a priori*, based on their size, preservation and shape could not be rejected as potential hominin fragments, were selected for the ZooMS analyses. All these fragments come from the bag collected in the eastern half of square 1 in 1970 in the Mousterian layer, which provided additional LF8 remains (7), but for which more exact 3D information is not available. This bag was identified in the collections of the MAN and contains objects coming exclusively from the same archaeological assemblage that contained LF8. Among those specimens, 5 were selected for radiocarbon dating. In addition, 14 bone remains from layer L2Bj (the layer overlying where LF8 was found) were analyzed to compare deamidation data with the specimens associated with the LF8 child. These last 14 specimens were sampled from another bag, clearly identified as containing samples from this layer.

Luminescence dating

The four samples for luminescence dating were collected in the northern profile, west to where LF8 was found (Figure 1; *SI Appendix*, Fig. S10). We cannot directly correlate those samples with the area where was found LF8 several years before, as the complete sediment volume enclosing the individual was fully excavated at that time. These samples were collected in December 2014, *i.e.* after the excavations in the LF8 sector. Sample 1 is at the same elevation as layer L2bj, samples 2 to 4 come from a deeper sedimentary context, roughly at the same depth as

the excavated Mousterian assemblage where LF8 was found. Due to the inclination of this archaeological assemblage, sample 4 is presumed to be the closest sample to LF8's original position.

The samples were collected under the opaque covering to avoid exposure of the samples to sunlight and after removal of the first 2 cm of exposed surface. The methodology followed the standard luminescence dating protocols, already conducted at the site in its western area (11): wet sieving, followed by a standard series of chemical preparation steps (treatment with HCl, H_2O_2 and HF), was conducted to extract 180-250 µm K-feldspar- and quartz-rich fractions from the samples; these two mineral fractions were isolated by heavy liquid density separation. Luminescence signals emitted by these fractions were then measured: post-IR IRSL (pIRIR) signals from multi-grain aliquots at elevated temperatures (160°C and 290°C, respectively; see (11), following the $pIRIR_{290}$ protocol (12)) from the K-feldspar extracts, as well as quartz single grain OSL (13) using the SAR protocol (14) were measured on Risø TL/OSL DA-20 readers at IRAMAT-CRP2A, using standard filter combinations. Curve fitting (using single saturating exponential curves) and grain selection (based on the uncertainty on the first test dose response signal, and the D_0 curvature parameter proposed by Thomsen et al., (15)) are the same as those used by Guérin et al. (11).

Gamma dose rates were measured using *in situ* Al_2O_3:C dosimeters, inserted in the sediment at the location of each sample and left at the site for 6 months. Beta dose rates were determined at IRAMAT-CRP2A by high-resolution gamma spectrometry. Dose rate conversion and correction factors were taken from Guérin et al. (16, 17, 18). *SI Appendix*, Table S2 summarizes all dose rate information.

Radiocarbon dating

A total of 17 fossil samples were radiocarbon dated (*SI Appendix*, Table S1), including 5 that were analyzed by ZooMS (R-EVA 3336-3340). We had a sampling strategy intended to collect as much information as possible to properly chronologically contextualize the child skeleton. Twelve small bone fragments found together with the LF8 individual, from the 1970-1973 excavations, and for which we have good contextual information were selected for radiocarbon dating (*SI Appendix*, Table S1 and Fig. S10). We selected specimens that could not be anatomically and taxonomically recognized for conservation reasons but also to maximize the chance that one of them could be a hominin remain. One additional fragment excavated in 1970 from the complex L2B-LBj overlying the Mousterian one (where LF8 was found) was also dated. Four bone remains recovered during the 2014 excavations to the west or east of square 1 at the elevation of the L2B and L2Bj layers were also dated. This allowed obtaining ages at the border of the originally excavated area.

Bones were pretreated at the Max Planck Institute for Evolutionary Anthropology (MPI-EVA). Roughly 600 mg of the bone is decalcified in 0.5M HCl at room temperature until no CO_2 effervescence is observed. A 0.1M NaOH is added for 30 min to remove humics. The NaOH step is followed by a final 0.5M HCl step for 15 min. The resulting solid is gelatinized following Longin (19) at pH 3 in a heater block at 75ºC for 20 h. The gelatine is then filtered in an Eeze-FilterTM (Elkay Laboratory Products (UK) Ltd.) to remove small (>80 µm) particles. The gelatine is then ultrafiltered with Sartorius "Vivaspin Turbo" 30 kDa ultrafilters (20). Prior to use, the filter is cleaned to remove carbon containing humectants (21). The sample is lyophilized for 48 h. To monitor contamination introduced during the pre-treatment stage, a sample from a cave bear bone, kindly provided by D. Döppes (MAMS, Germany) (22), was extracted along with the batch from the human specimen. Elemental and stable isotopic data (%δC and %δN content, C:N, $\delta^{13}C$ and $\delta^{15}N$) of extracted collagen was measured in-house at the MPI-EVA, and about 5–6 mg was weighed into precleaned tin cups at the MPI-EVA and sent to the Klaus-Tschira-AMS facility (lab code: MAMS). The sample was combusted in an EA, and CO_2 was converted catalytically to graphite and dated using the MICADAS-AMS (23).

Bones R-EVA 3336-3340 were selected for dating following analysis by ZooMS. Due to their very small size, 30-104 mg were pretreated for ^{14}C dating using a modified version of the protocol described above specifically for <100 mg bone (24), alongside three aliquots of background bone weighing 38-87 mg. Roughly 2-3 mg collagen was weighed into precleaned tin cups and sent to ETH-Zurich (lab code: ETH) where they were graphitised using the Automated Graphitisation Equipment (AGE) 3 (25) and measured using a MICADAS AMS (26).

ZooMS analysis

Seventeen bone specimens coming from the layer where was found LF8 were analyzed by ZooMS (Zooarchaeology by Mass Spectrometry) in the hope to identify any hominin bone specimens amenable to direct radiocarbon dating. One of these samples concerned a directly dated bone (R-EVA 1607), while the others remain undated. ZooMS extraction followed a workflow established previously (27) by initial ammonium-bicarbonate extraction and MALDI-TOF-MS spectrum acquisition (m/z 900-4,000). Bone samples were only demineralized if initial ammonium-bicarbonate buffer screening failed to provide sufficient peptide markers for taxonomic assignment. An extraction blank was processed alongside the Pleistocene fauna to ensure no collagen contamination in the laboratory occurred.

The 17 studied bone specimens derive from the area were LF8 was found, although their exact coordinates are unknown. They all came from a bag identified in the MAN (see above). Because of the fragmentary material studied by ZooMS in this bag, these objects were not labeled at the time of their discovery in the field in 1970.

MALDI-TOF-MS peptide mass fingerprints of collagen were compared to a list of diagnostic peptide mass markers for mammals in existence in Europe from MIS5e onwards (27, 28). Glutamine deamidation was calculated quantitatively for peptides P1105 and P1706 (following Van Doorn et al. (29)).

In addition, 14 bone remains from the overlying layer, L2Bj, sampled in the collection of the MAN, were analyzed to compare deamidation data with the specimens associated to the LF8 child.

Analysis of ancient mitochondrial DNA

Laboratory work for ancient DNA analysis was carried out in a clean-room at the Max Planck Institute for Evolutionary Anthropology (Leipzig, Germany). In a first experiment, 9.5 mg of bone powder was removed the LF8-associated hominin specimen identified by ZooMS. Following a previously described protocol for the extraction of highly degraded DNA (30), the sample material was digested with 500 µl lysis buffer and the DNA purified from 150 µl of the lysate using silica spin-columns and buffer 'D' for DNA binding. To replicate the results of the first experiment, a second DNA extraction was performed at a later time using another 150 µl of the same lysate. In addition, DNA was extracted from a 150 µl aliquot of lysate prepared from a second sample (~11 mg) of bone powder. was All three DNA extracts were converted into DNA libraries using an automated protocol for single-stranded library preparation (31). Negative controls were included both during DNA extraction and library preparation to monitor DNA contamination in the laboratory (*SI Appendix*, Table S3).

As detailed in the aforementioned protocol, quantitative PCR assays were performed to determine the number of unique molecules in each library and to evaluate the efficiency of library preparation based on the conversion of a control-oligonucleotide that was spiked into each reaction (*SI Appendix*, Table S5). All libraries were then amplified using a pair of primers containing sample-specific index sequences, purified, and their concentration determined on a spectrophotometer. Enrichment for hominin mitochondrial DNA (mtDNA) was performed through two successive rounds of on-bead hybridization capture (32) following a protocol described

elsewhere (33), using probes designed based on the revised Cambridge Reference Sequence (rCRS) (34) and printed on a microarray (35).

The enriched sample and control libraries were pooled and sequenced together with libraries from other experiments on partial lanes of a MiSeq sequencing machine (Illumina) in 2x 76 cycles paired-end mode (plus 2x 7 cycles index reads). After assigning sequences to individual libraries based on perfect matches to the expected index pairs, overlapping paired-end reads were merged into full-length molecule sequences using leeHom (36). Merged sequences were aligned to the rCRS using BWA (37) with parameters adjusted for ancient DNA (38). Sequences shorter than 35 base pairs and with a map quality score smaller than 25 were discarded. Duplicate sequences were removed using bam-rmdup (https://github.com/mpieva/biohazard-tools/) and all unique sequences investigated for the presence of C-to-T substitutions at their ends (*SI Appendix*, Table S4), which result from deamination of cytosine to uracil in ancient DNA (39).

The affinity of the sequenced mtDNA fragments to present-day human and Neandertal mitochondrial DNA was assessed by determining their state at diagnostic positions in the mitochondrial genome where at least 99% of a set of 311 present-day human sequences (40) differ from 10 Neandertals (Genbank accessions FM865411, FM865407, FM865408, FM865410, AM948965, KJ533544, KJ533545, FM865409, KC879692 and KF982693). Thymines occurring in the first or last 3 positions of each sequence were disregarded to reduce the impact of deamination. The same analysis was also repeated using putatively deaminated fragments only, which were identified by filtering for sequences with a terminal C-to-T substitution (*SI Appendix*, Table S5).

Taphonomic study

Taphonomic analyses concerned all the fossil remains associated to LF8, including the faunal elements and the LF8 Neandertal child, including the faunal elements and the complete LF8 (1, 7). For each series, we report the total number of skeletal remains (NRT), the number of identified specimens (NISP), the minimum number of elements occurring (MNE) and the minimum number of individuals present (MNI) (41, 42). We recorded the dimensions (length, breadth and thickness) and anatomical, taxonomic, and modification data for all recorded and identified specimens and for unrecorded fragments more than 25 mm long. Indeterminate fragments less than 25 mm were only used for fragmentation studies (type and size classes) and carbonization analyses. The identification of the type of breakage (ancient green or dry bone fracture or recent fracture) was made based on the fracture color, shape, feature and angle and associated marks (43-45). The shaft fragments were differentiated by size and circumference classes (43): $L1 \leq 1/4$; $L2 \leq 1/2$; $L3 \leq 3/4$ and $L4 \leq 1$; $C1 < 1/2$; $C2 \geq 1/2$ and $C3 = 1$. For indeterminate fragments, we established two main size categories adapted to the ungulates present in our sample: 1) middle-sized ungulates with a body mass between 100 and 300 kg (red deer and reindeer) and 2) large-sized ungulates with a body mass between 300 and 1,000 kg (large bovids and horse).

Ontogenic age-at-death of prey specimens was based on dental eruption/replacement patterns and wear. We established four age groups: juveniles (with deciduous teeth), young adult (with erupted P4 and M3), prime adults (with moderately worn P4 and M3) and old (with heavily worn teeth) (46-47).

We observed the bone surfaces with the naked eye, and we examined and photographed some elements to distinguish the various surface alterations using a Dino-Lite Digital Microscopes (AD7013MZT, magnification 10-240x). Images were recorded with a DinoEye USB camera (AM423XC) and DinoCapture 2.0 software. We recorded types and locations of relevant modifications on the outer surface, including those made by rodents, carnivores or hominins as well as climatic and edaphic modifications. The latter include cracking, desquamation, polish, concretion, root marking, chemical corrosion and oxides coloration. The identification of the main

taphonomic modifications followed standard procedures (48-51). The illegible remains were not included in the percentages.

We distinguished specifically trampling marks from butchering marks using previous works (49, 52-57). The location and morphology of the latter may indicate the butchering activity related to them including evisceration; skinning; dismemberment; disarticulation; periosteum removal; cutting tendons and defleshing (49, 58-59).

We classified carnivore marks as follow: pits, punctures, scores, notches or corrosion by gastric acids (49, 60-61). We took measurements of pits, punctures, scores and notches (maximal length – L – and breadth – W –) into account, as well as tissue location (cancellous bone or articular portions; cortical or median diaphysis; thin cortical bone or diaphysis extremity) (62).

Lithics

The lithic material originally found during the 1970-1973 excavations and that was associated with LF8 or coming from the L2B-L2Bj complex was revised. This material was housed at both the MNHN and the MAN.

Geology

New data from the 70's excavation: On a visit to the MNHN on 12 February 2015, a geological sample was collected, from a bag of loose, previously sieved sediment. This sediment was taken from the area of the LF8 specimen in what is now called the northern sector and was collected in 1973, during the Delporte excavations. This sediment comes from just beneath the level where the occipital bone was found. The sample studied here in hand lens is composed of loose, yellow sand with small mm-sized pieces of black, burned bone. These bone fragments are similar to those found in the so-called "Yellow" layer observed during the 2014 season, which was exposed roughly 1-2 m WSW of the general location of LF8. This sample of loose sediment was brought to the Geoarchaeology Laboratory at the University of Tübingen, where it was consolidated with polyester resin, trimmed, and processed into a 60 x 90 mm thin section by Mr. Panagiotis Krititkakis. The thin section was examined in plane-polarized (PPL) and cross-polarized (XPL) light, at magnifications varying from 25x to 200x.

New data from the field: Observations on the stratigraphy and geology of the LF8 (northern) sector were conducted directly in the field during the 2014 excavations. It permits a direct comparison between the stratigraphy reported at the time of the Delporte's excavations and our own observations after the exploitation of all the information recorded during these original excavations. This allowed for a precise contextualisation of LF8 within the site. However, we could not study directly the sediments that were closely associated with the discovery as our recent excavations revealed that the sedimentary context of LF8 was completely excavated in 1973.

Supplementary Text S2. Supplementary Results

Detailed information from the archives of the 1968-1973 excavations directed by Henri Delporte about the context of the discovery of La Ferrassie 8

Part of the reason to re-open La Ferrassie was related to the INQUA (International Union for Quaternary Research)VIIIth congress. Once it was known that it was going to be held in Paris in 1969 and that a good number of their members were asking to visit the important sites of the Périgord, it was necessary to undertake new work at the *grand abri de la Ferrassie* site in order to show the site in a good state 40 years after the previous excavations (63). Thus, from 1968 to 1973 six field seasons were carried out, each of one month of duration, with the following objectives:

1-Show a correct (stratigraphic) profile of the site to the participants of the congress;

2-Define the stratigraphy;

3-Take samples and record the necessary observations in order to perform sedimentological and palynological analyses (among others).

Delporte (63) provides additional information on the squares excavated during each of the six field seasons. Two orthogonal profiles were excavated (*SI Appendix*, Fig. S3):

1) the first, "*coupe frontale*" or frontal profile, parallel to the wall of the rock-shelter (approximately E-W direction) included squares 1 to 9 and 13 to 16, advancing around a meter from where Peyrony's excavations ended, particularly for the damaged bank of sediment in the basal part of the frontal profile (*SI Appendix*, Fig. S4).

2) the second, "*coupe sagittale*" or sagittal profile, perpendicular to the previous, included squares 10-12 and 50-60 advancing around 50 cm from the profile left by Peyrony.

The principal information from the Delporte archives related to the LF8 individual may be summarized as follows:

1968-1969: in 1968 and 1969, only a limited amount of information was obtained on the basal layers of the stratigraphy, as excavations did not reach layer M (in which LF8 was found). A roof was installed before the summer of 1970 to protect the ongoing work and the cleaned stratigraphic sections.

1970: in 1970, the excavations, involving 22 people, took place between 1st and 31st August. One of the important events of this season concerned square 1. A small trench with a width of 50 cm was excavated perpendicular to the wall of the *abri* (the green area on *SI Appendix*, Fig. S5). This area corresponds approximately to the eastern half of square 1. The profile resulting from the trench yielded more information on the structure of the red complex that corresponds to the so-called "lower Perigordian" (i.e., Châtelperronian). According to the excavation report from 1970, this trench yielded several human remains that were identified during the excavation:

A) One isolated adult tooth from the layer L2bj, which is above the Mousterian layers. This tooth, labelled 168, was found on 19th August 1970 by Ernest Collard at the coordinates X=62, Y=151 and ΔZ=626.5.

It should be noted that: 1) numerical labelling is continuous, starting from 1, for each square and each field season; 2) based on the maps found at MNHN and MAN, while the X increases eastwards, the Y increases southwards, and thus the (X=0, Y=0) point, instead of being at the south-western corner, as it is usual in many modern excavations, it was located at the north-western corner in Delporte's excavation; 3) The ΔZ results from the sum of the relative Z plus a fixed value (516.5 in 1970). Thus, the tooth labelled 168 had a Z=110 and thus ΔZ=Z+516.5=626.5.

B) Seven teeth, belonging to the same individual, a young child. These teeth were found after the screening the sediment from an area around X=70, Y=155 and ΔZ=660. No more information concerning this finding is present in the archives.

C) One cranial fragment. This cranial fragment (actually two fragments, but only one is mentioned in the notebook at the time of the excavations and in the report for 1970) was found on 21st August 1970, by Ernest Collard at coordinates X=64, Y=139 and ΔZ=657.5 The level that was designated Xb at the time of the excavations. The specimen was labelled 219.

The report of the 1970 excavations mentions that the child teeth and the cranial fragment(s) could have come from a burial that would have been disrupted by solifluxion and that the upcoming excavation would provide more information on this topic. This speculation was confirmed in 1973 when additional LF8 remains were found. This shows that LF8 was found in 1970 rather than in 1973, which is the most common date used in publications.

The fact that both the adult tooth and the cranial fragments were found on the same day with a 31 cm difference in depth (see above) indicates the speed of the excavation. In 1970, the layers underlying L2bj were temporarily named Xa and Xb, and later labelled M2. The uppermost extension of this layer was said to be poor in stone tools, containing a lot of silex fragments, numerous charcoal fragments and burned bones. However, from ΔZ 636.5 to 642.5 (at around X=41 and Y=130) nearly nothing was found. At ΔZ 646.5 (X=41; Y=130) and ΔZ 643.5 (X=41; Y=160) the sediment shows a yellow color and has the same structure. In the whole area, several small slabs (*dallettes*) were found in a horizontal or slightly inclined position. Most have an elongated shape and their longer dimension followed an east-west axis. The *dallettes* were found above all the elements taken from the Mousterian layer, including the new human remains that have been recognized in the collections. We have not found other reference to nor illustration of these *dallettes* and could not identify them in the collections of the MAN. Below, the sediment was described as less argillaceous, drier and more crumbly, *dallettes* become rarer, and flint and charcoals reappear. There is a darker area between the points X=47, Y=122 (47,122), (47,138), (96,125) and (85,140), with a Z ranging from 141 to 147 (ΔZ= 657 to 663), which contains numerous charcoal fragments but it does not resemble a fireplace accordingly to the notes left by the person in charge of the excavation of the square. The lack of illustrations and of more detailed description of this area with numerous charcoals fragments does not permit further interpretation of this observation. This dark area is just below the level of all the objects that had been found in this area, so potentially just below the area where a part of the head of La Ferrassie 8 was located. The layer Xb (which corresponds to M2) at coordinate 100 N-S (the Y axis) becomes nearly sterile in tools and bones accordingly to the excavation notes, so only a very few archaeological finds were found close to the wall of the *abri* in the east half of square 1. Based on the available information in the archives, it seems that in 1970 the Mousterian layers were excavated in square 1 only on August 20th-21st and no further records of this square were made. The reader should recall that the main objective of creating the profile that resulted from this trench was to obtain more information about the structure of the overlying layers, not to study the Mousterian layers.

<u>1971:</u> The excavations in 1971 took place between 2nd and 30th August and involved 16 people. The Mousterian layers were not excavated during this period, and no excavation was carried out in square 1.

<u>1972:</u> The excavations in 1972 took place between 31st July and 31st August and involved 23 people. Most of the work concerned the sagittal stratigraphic profile (i.e. perpendicular to the wall of the *abri*, N-S orientation). The excavations of the Aurignacian (squares 1-10) and Mousterian (squares 2-3) levels continued in the frontal stratigraphic section and yielded abundant archaeological material. The notebook for square 1 mentions that the excavator in charge of this area during this season only excavated the layer just above the Mousterian horizon. An isolated page dated from 10th August contains some general notes, including a comment about squares 2 and 3. The L layers of these squares were excavated by Jean-Paul Gaborit and J.C. Lecq and they mentioned that it was

not useful to excavate layer M (with no distinction of sublayers M1 and M2 as in square 1) as it appeared to be sterile in squares 2 and 3. In square 2, only 10 objects were plotted from layer M in a volume of 75 cm (X) by 35 cm (Y) by 35 cm (Z) in layer M. This illustrates the very low density of archaeo-paleontological finds despite the volume of the M layer that was excavated here.

<u>1973:</u> The excavations in 1973 took place from 1st-31st August. The person in charge of square 1 summarized some information on the observed stratigraphy:

L2bj: red layer rich in silex and with small rounded elements;

M1: brown layer nearly sterile with imbricated small slabs (*dallettes imbriquées*);

M2: yellow layer, crumbly and dry.

The first elements attributed to LF8 collected in 1973, and recognized as probably being human remains, were found on August 24th by Ernest Collard and Jean-Paul Gaborit when they started to excavate the Mousterian layer. The first human element is labelled 295. In the week from August 24th-to 30th, elements up to label 538 were collected including all the LF8 remains which were recognized at that time, and all the fragments of the bison horn-core that we have reconstructed, even though this object was not recognized at the time of the excavations.

From August 29th, the excavations extended laterally affecting the western limit of square 1. Instead of naming a new square, they were included within Square 1 using negative values for the X axis. Human remains were identified as far as coordinate X=-27. Apparently, the excavation of layer M2 did not continue further west.

All human elements recognized as such in 1970 and 1973, and several faunal remains associated to layer M2 from square 1 (excavated in 1973) were sent for study to Jean-Louis Heim (the "brown box"). The MAN curated bags with bone and lithic debris from the Mousterian layers excavated in 1970 and 1973 from the area where LF8 was found, which were not revised by Heim (Heim, pers. comm.). The fact that numerous new human remains have been recently identified in these bags indicates that this part of the collection had not previously been revised by anthropologists.

From the available photographic material (8, 63) it is clear that Delporte's excavations had a limited impact on the frontal profile. For example, the red line on *SI Appendix*, Fig. S4 shows a very similar course of the upper extension of the preserved sediment on this profile between 1968 and 1973. The area that was more intensively excavated was a bank of sediment preserved up to two meters from the profile, as shown by the green line on this figure. This allowed the precise identification of the location of square 1, and thus of the place where LF8 was found.

Additional and unpublished photographies and diagrams were also found in the archives. The main element concerning LF8 is a map on millimetric paper showing the position of the fossil elements found in layer M2. This drawing was started on August 24th 1973 with the specimen labelled 295. Another version of this map was in the "brown box" and provided the original information for the map of LF8 published by Heim (1) (*SI Appendix*, Figs. S7 and S8).

A letter dated October 1st 1973 was also found. It was addressed to several excavators that were in charge of square 1 and explained that the video and the photographs of the human remains had been lost and asked for any other photographs that they might have. Fortunately, a letter dated October 12th and written by Christophe Toupet is associated with six photographs of square 1, and these are the only photographs of the excavation of LF8 (*SI Appendix*, Fig. S8). Based on the visible elements and the information deduced from the archives it is highly probable that these photographs were all taken on 27th August 1973. The occipital bone of LF8 is visible in all the photographs except one, where it is obscured by an excavator. This was the last element to be unearthed on August 30th and is labelled 538. Label 537 corresponds to the adjacent area to the south where 10 x10 cm of sediment was sampled for screening. Excavations were, therefore, stopped at a depth

corresponding to the inferior vertical extension of the occipital bone as visible on the photographs (*SI Appendix*, Fig. S10).

At the end of the 1973 field season, the unexcavated surface of the Mousterian layer in square 1 and adjacent areas was protected by a canvas cover. Moreover, a wall was built in this area along the frontal profile and the canvas was covered with small stones. The objective of this construction was to protect the excavated areas and the profile against possible damage by visitors to the site.

SI Appendix, Fig. S5 shows a general view of the excavated area of square 1. This photograph is the largest view available for this sector and was taken on August 27th 1973. In this figure, the excavated area corresponding to the elements of LF8 found in 1973 is located in front of the excavator. The extension and location of the human remains are represented by the area colored in red. The blue lines border the original extension of square 1 as defined for these excavations. The area in green corresponds to the trench excavated in 1970 where a parietal fragment and several teeth were identified.

Spatial distribution of the M2 layer and of the La Ferrassie 8 elements

The notebooks provide detailed information on the spatial distribution (XYZ coordinates, the orientation of the elongated elements) of the 1968-1973 findings, including lithic, human and faunal remains. This helps us to understand the characteristics of the M2 Mousterian layer, where LF8 was found, such as the density of the findings, their general spatial organisation and inclination, relative to the other layers.

First, the findings in layer M2 are restricted to the area where LF8 was found (i.e., square 1, excavated in 1970 and 1973) and the density of this findings is lower than the overlying layers (L2B and L2Bj in particular). Layer M2 is archaeo-paleontologically “sterile” in the area east of coordinate 50 on the X axis and toward the north from coordinate 100 on the Y axis. Square 2, to the east of square 1, also produced very few finds in layer M2. Moreover, the M2 layer was not excavated west of coordinate 50 on the X axis and north of approximately coordinate 110 on the Y axis. Similarly, no excavation took place in the full north-south extension of square 1 to the west of coordinate 35. Laville and Tuffreau (64: 45) note that in the frontal (longitudinal) section layer M was affected by cryoturbation and thus they defined this deposit from a geological point of view in zones 54-60 on the sagittal section (*SI Appendix*, Fig. S3).

Moreover, despite the differences in the density of the recorded finds from these years there is a noticeable absence of archaeo-paleontological findings between the recorded archaeological artefacts in layer M2 and the overlying layer in the western part of square 1 (*SI Appendix*, Fig. S6). Indeed there is a sterile area which reaches a vertical extension of >10cm at X=0 and >30cm at X=-25 above the human remains and associated finds (*SI Appendix*, Fig. S6). This lacuna is not the result of a lack of information as we know that the area was very carefully excavated in 1973. In this case, the absence of finds is therefore not due to an absence of information but to the complete absence of archaeo-paleontological objects in this part of layer M2. Finally, the archives also mention that the underlying layers below M2 were sterile.

In square 1 and layer M2, the difference in the density of the recorded finds between 1970 and 1973 is the result of the faster excavation in 1970 and the more careful excavation in 1973. Despite the faster excavation process in 1970, the archaeo-paleontological findings from the different layers (including square 1-M2 layer) were kept in bags with the information of the layer and square, despite the absence of XYZ coordinates. In fact, one of these bags yielded most of the new LF8 remains described by Gómez-Olivencia et al. (7). In any case, the 1973 excavation yielded many human remains which were identified as such and the field, which resulted in a more careful recording of most of the archaeo-paleontological findings.

Regarding the inclination of the archaeo-paleontological layers, as assessed by the spatial distribution of the individual plotting of the findings, M2 is not parallel to the overlying levels.

Layers L2, L2bj and M1 from square 1 slope downward the east, and to a lesser extent north (61) as can be seen in the frontal (east-west, i.e., parallel to the wall of the *abri*) and transverse (north-south, i.e., perpendicular to the wall of the *abri*) profiles (*SI Appendix*, Fig. S6). However, while the general geological inclination of the M2 layer follows this general slope downward east, the general tendency of the archaeo-paleontological assemblage within M2 on its limited extension is to slope downwards to the west (*SI Appendix*, Fig. S6).

While there is no reference to anatomical articulation in the excavation notes, the XY coordinates of the LF8 remains shows that there is a general anatomical consistency in the arrangement of the elements, also noted by Heim (1). The cranial remains, uppermost ribs and cervical vertebrae appear to the east and pelvic elements and lumbar vertebrae to the west. The fact that the new LF8 remains found in the 1970 excavation (which involved the eastern sector of square 1) are cranial and cervical is consistent with this observation. The LF8 remains are scattered in the west-east axis over an area of 94 cm. This extension is calculated from the westernmost element found in 1973 (X=-27, label 533) to the easternmost cranial elements found in 1970 (X=67, label 219). Some human remains were not coordinated in 1970 because they had not been recognised as such during the field work. However, we estimate that this distance is a good approximation of the distribution of the human remains based on the information we gathered. The dispersion of the LF8 elements in the north-south direction (Y axis) is low (~20 cm), and just one hand phalanx was found 10 cm further to the south. We cannot exclude that some other elements were initially present but not collected or lost particularly if the area was exposed after the end of the initial excavations in 1922. When summed with the depth (Z axis) value of each of the LF8, elements, we see that apart from the E (head)-W (pelvis) orientation, the cranial parts are higher topographically than the pelvis (~30 cm).

Results of the 2014 excavations

The 2014 excavation in the LF8 sector took place sequentially: first, an area measuring 50 x 100 cm located west and south to the area dug during the Delporte's campaign was excavated; second, a small column of sediment was preserved in the north-western corner of square 1 (*SI Appendix*, Fig. S11 and S12); finally, the surface of the square 1 where LF8 was found was cleaned and further excavated.

In the area west to the Delporte's excavation, it was possible to identify Delporte's L complex, containing three sublayers with abundant lithic and faunal remains, including Châtelperronian tools. Below, three layers, one brown, one yellow and one red were identified. These three layers were sterile in archaeological finds. This first phase of the excavation finished once the bottom of the excavated area in square 1 in 1970-1973 was attained (*SI Appendix*, Fig. S11).

Regarding the column located in the north western corner of square 1, it was completely excavated before the cleaning of the basal surface of square 1. As visible in Fig. S11 this sedimentary filling produced a lot of archaeological objects in its upper most extension, but none in the underlying layers. In this context, the few objects that were recorded along the western extension of square 1 were, in fact, all found in the dirt layer resulting from the 1973 excavations and subsequent procedures to protect the site.

The area where LF8 had been found in 1970-1973 was completely cleaned and subsequently excavated. After a few cms of sediment were excavated a level containing numerous blocks of limestone was reached. These blocks are elongated, with a maximal extension of around ten to twenty centimetres. This layer was sterile from an archaeological point of view. The sediment that was excavated above probably corresponds to a layer of dirt resulting from the 1970-1973 excavations, the closing and covering of the sector and its recent cleaning. Indeed, the colour of the

sediment was different from the one that was preserved associated to the bones of LF8 in the collections of the MAN.

This sediment yielded a few archaeological findings, including a hominin tooth fragment (described below; pink point in Fig. S9 and S10). However, as this excavation area was surrounded by profiles with archaeo-paleontological material, the fact that 40 years have passed since the last excavation by Delporte to our excavation, and the fact that this area was covered for preservation purposes, we cannot rule out that these few findings fell from any of the surrounding profiles.

The human tooth (*SI Appendix*, Fig. S12) found in 2014 is a lower molar crown fragment, which is not preserved over its entire crown height, representing a bit more of the bucco-mesial quadrant of the tooth (enamel and dentin are preserved). The occlusal surface of the cusps shows a facet of occlusal wear. It is therefore certain that this tooth was functional. The enamel does not look very thick and because of the state of conservation, we cannot observe the top of the pulp chamber. The presence of a very deep buccal groove on the best-preserved vertical face of the dental crown, as well as its position relative to the occlusal morphology of the cusps, assures us that the tooth is a lower molar. Indeed, it is the groove present on the buccal surface of any lower molar and separating the protoconid (vestibulo-mesial cusp) from the hypoconid (central vestibular cusp). Thus, this molar fragment preserves the entire protoconid and virtually the mesial part of the hypoconid. A small part of the base of the metaconid (mesio-lingual cusp) and one square millimeter of that of the endoconid are preserved. 2.5 mm in front of this vertical groove, another 2 mm high groove is observed which affects the vestibular aspect of the protoconid much more discreetly. It is a discrete character that is not well documented. A furrow in an equivalent position but that is more pronounced was noted on the permanent lower first molar germ of Genay (Côte-d'Or, (65)). Given the state of preservation, a small part (buccal) of the mesial dimple is also present. It is bordered distally by a crest of enamel which - according to the preservation state of the tooth - is continuous. This feature on permanent teeth is considered to be derived in Neandertals (66)). Despite the fact that no standard measurement can be taken on this fragment, it seems large when compared to a recent human molar. All these features are consistent with this being a Neandertal lower left molar, without being able to further determine its anatomical position. Finally, these characteristics make it impossible to relate this tooth fragment to La Ferrassie 8, whose germ of the first lower right molar is preserved (1).

According to the recent description of new hominin fossils (6), this additional tooth is named La Ferrassie 13 (LF13, *SI Appendix*, Fig. S13). Due to the uncertainties regarding the original stratigraphic provenance, and the fact that another adult tooth was found in square 1 (LF7), it is not possible to propose this tooth as representing an additional Neandertal individual. As a result, there are currently a minimum of four adult, two represented by nearly complete skeletons - LF1 and LF2- and two represented by teeth -LF7 and LF12-, and five immature -LF3, 4, 5, 6, 8- Neandertal individuals represented in the LF collection (6).

Finally, the 2014 fieldwork certificated that the layer containing LF8 and the associated findings was completely excavated during the 1970-1973 excavations.

La Ferrassie 8: new human remains

Forty-seven human remains, including cranial remains, mandibular fragments, vertebral and costal remains and two hand phalanges, significantly complete the LF8 individual (7). However, despite completing this individual, this individual's anatomical representation is still limited to the skull, trunk, pelvis and only four hand phalanges.

The attribution of the new human remains to LF8 is based on their consistency with the existing remains in terms of size and age-at-death, the lack of anatomical duplication, the direct refitting of bone elements, and their location in the deposit (they are from the same square, same layer and similar coordinates within the referential used during the excavations in the 70th). i.e.,

From an anatomical point of view, the recently discovered fossils do not overlap with the LF8 original collection. Moreover, some of the new elements (e.g., a wormian bone and a lumbar fragment) refit anatomically with elements of the original collection. The new remains show developmental stages compatible with those of the original elements as illustrated by the state of development of the tooth identified inside the mandible that is is compatible with the LF8 original teeth collection. The length of the pars basilaris is also compatible with this association as are the general dimensions of the phalanx.

All the fragments from the original LF8 collection were also revised, resulting in the identification of an additional hand phalanx and the improved identification of several anatomical elements. The human remains show a very good state of preservation (see below): very small and fragile elements are preserved, they have an exceptional preservation of their surface without smoothing or exfoliation, many represent complete anatomical units, and the structural and internal features of the cranial bones are very well preserved. Finally, the revision of the LF8 remains curated at the MNHN revealed that one rib fragment (labelled 397) and represented on Heim's map, actually represent a faunal remain (7). An additional hominin fragment has been identified by ZooMS. Due to the limited remaining material after ZooMS and radiocarbon dating, it was not possible to detect ancient nuclear DNA. However, mitochondrial DNA firmly attributes the specimen to Neandertals. In addition, the clear association of this fragment with the archaeological assemblage that contained LF8 and the fact that all the identified human bones in this area belong to LF8 make that the most parsimonious attribution of the fragment is it was a piece of LF8.

The bison horn-core

We also identified a partial bison horn-core from the faunal remains that were found at the same time and in the same level as LF8. On June 14th 2013, fragments of a large bovid horn-core were identified among the faunal elements kept in the "brown box". All the fragments that could belong to the horn (around 20) were separated from the other faunal remains. They were in individual bags with small paper labels identifying them as part of the 1973 findings, but only a few specimens were directly physically labelled. After realizing that some fragments could be refitted together, permission was asked to Alain Froment (responsible at that time for the Anthropology collections of the MNHN in which the horn-core was found) to clean the fragments and physically label those that were not labelled. In the following days, the reconstruction was carried out and led to the refitting of 20 fragments into three larger pieces that also fit together and constitute a large fragment of a bison (*Bison sp.*) horn-core. Five isolated additional fragments were recognized. A fragment of the horn-core (labelled "X") was sent for direct dating to Paula Reimer of the Centre for Climate, the Environment & Chronology (14CHRONO), School of Geography, Archaeology and Palaeoecology, Queen's University Belfast. Unfortunately, no date was obtained due to the specimen's preservation.

OSL dating

Both pIRIR and OSL signals characteristics emitted by K-feldspar and quartz grains from the sediments of La Ferrassie already showed that the samples were amenable for dating. In particular, the previously studied samples from this site passed several acceptance tests (preheat plateau, dose recovery, etc.) commonly conducted in luminescence dating (67). Moreover, the comparison between OSL and radiocarbon showed a good agreement between these independent ages (11).

However, one sample (FER 2) from the main recent excavations sector (Layer 6, in the western area of the site) appeared to have been 'poorly-bleached', *i.e.* some of the studied grains – both quartz and K-feldspar – had not been sufficiently exposed to sunlight before burial by overlying sediment to completely reset the OSL signal. The geomorphological study of the sediment

from this layer indicated that bedrock decalcification could be a source of poorly-bleached grains in the sediment, and thus could explain the overestimated luminescence ages (both $pIRIR_{160}$ and OSL) for this sample (11).

Given this prior knowledge of the site, we paid special attention to bleaching problems for the LF8 sector samples, all the more since this area is close to the bedrock wall to the North of the site – so the samples were quite far back in the cave system and could have been affected by bedrock alteration. First, we calculated Single Grain OSL (SG-OSL) ages (*SI Appendix*, Table S6), assuming that all samples were well-bleached – we applied the Central Dose Model (CDM: Central age model in (68)) to the equivalent dose (D_e) distributions. The obtained ages increase with depth and do not show any stratigraphic inversion, which is an argument against poor-bleaching (conversely, stratigraphic inversions would have been a strong argument to suspect bleaching and/or post-depositional processes affecting the OSL ages).

However, the next step led us to compare the multi-grain pIRIR ages with the SG-OSL ages, still assuming that quartz OSL signals were well reset before sediment burial (*SI Appendix*, Table S7). It appears that pIRIR ages strongly overestimate the quartz SG- OSL ages, by an amount ranging from 92 to 370 % for $pIRIR_{160}$ and from 350 to 460 % for $pIRIR_{290}$ signals. Thus, it is clear that K-feldspar grains were not sufficiently exposed to sunlight prior to sediment deposition to be used for dating. At this stage, we compared the pIRIR to SG-OSL age ratios obtained for the LF8-area samples to those obtained for the samples taken in the western sector of the site (11). The sample for which these ratios are the lowest, LF8-2, gives a pIR_{160} (resp., pIR_{290}) to quartz age ratio equal to 1.9 (resp. 3.6) and is thus comparable with the poorly-bleached sample FER 2 (11). As a result, this comparison of K-feldspar and quartz ages indicates that all four samples from the LF8 sector are likely to have been affected by bleaching problems.

A third way to detect bleaching problems, in conjunction with the quartz-feldspar comparison, is to look at two characteristics of the SG-OSL D_e distributions: the fraction of grains in saturation (saturation being defined as a L_N/T_N ratio – L/T ratio for the natural dose cycle – greater than or indistinguishable from the saturation level of the laboratory dose response curve) and the overdispersion values calculated using the CDM (*SI Appendix*, Table S6) in comparison with overdispersion values obtained for samples from the same site and of comparable age. The average overdispersion for well-bleached samples in Guérin and collaborators (11) is 35 ± 4 %; thus, the overdispersion values obtained for samples LF8-3 (56 ± 3 %) and LF8-4 (63 ± 5 %) indicate potential bleaching problems for these samples; conversely, for samples LF8-1 and -2 (with OD values of 41 ± 3 and 42 ± 3 %, respectively) this parameter does not allow reaching a firm conclusion. Nevertheless, for all samples from the LF8 sector, even after applying the D_0>100 Gy selection criterion – which allow removing artificially saturated grains – a non-negligible number of grains in saturation are present in the D_e distributions. This observation clearly indicates bleaching problems and precludes the use of any central dose model, even although the degree of poor bleaching might be quite low for sample LF8-2 (the fraction of grains in saturation is 6 % for this sample).

Consequently, we calculated minimum ages for all samples, using two different models: the Minimum Dose Model (MDM: Minimum Age Model, (68)) and the Internal External Uncertainty model (IEU: (69)). These two models (see *SI Appendix*, Table S6) give statistically indistinguishable dose estimates, despite a somewhat important difference for sample LF8-1 (resulting in ages of 43 ± 6 using the MDM, and 54 ± 3 ka using the IEU). From a purely OSL perspective, it is difficult to choose between these two models.

Radiocarbon chronology

The results of the radiocarbon dating are presented in Table 1 and SI Appendix, Table S1. The results are discussed in the main text.

We have several dates obtained on faunal remains found in close association with LF8 in the same archaeological context and also from the archaeological layer located above (*SI Appendix*, Fig. S10). These last include one faunal fragment from the L2B-L2Bj complex that was found in 1970 and four fragments found in 2014 to the west and to the east of the originally excavated area. No other ^{14}C dating was possible below, laterally and just above the archaeological assemblage that contained LF8 as these sediments do not contain any faunal remain or lithic artefact.

OSL dating for the layer above the level of LF8 gives an age of 43 ± 6 ka (sample 1 on fig. 1) consistent with the ^{14}C of the faunal element from this layer (44.6-43.3 ka cal BP, 95.4%) excavated in 1970. Four contemporary dates were obtained on specimens sampled during the 2014 excavations on the west and on the east of square 1 at the elevation of the L2B and L2Bj layers (between 49.1-45.0 and 44.2-42.6 ka cal BP, 95.4%). While the sediment found at the same level as LF8 is dated at 66 ± 4 ka (sample 4 on fig. 1), ^{14}C dates (pretreated at MPI-EVA) obtained on ten faunal remains found together with LF8 in the M2 layer range from 45.5-45.5 to 40.4-39.5 ka cal BP (95.4%), a range whose extension partly covers but is also inferior to the dates found in the overlying level (Table 1). Finally, in addition to the ten faunal remains associated to LF8, one element (R-EVA 3336) recognized as a hominin remain by ZooMS, and as a Neandertal using ancient DNA analysis, has yielded an age of 41.7-40.8 ka cal BP (95.4%).

ZooMS results

A total of 17 bone specimens associated to the LF8 child were analyzed with ZooMS, one of which (R-EVA 1607, *SI Appendix*, Table S1) was directly radiocarbon dated prior to ZooMS analysis and five (R-EVA 3336, 3337, 3338, 3339, 3340) after the ZooMS analysis. The chosen specimens could not be identified taxonomically based on their external morphology and it was not possible to assign them to LF8 but it was neither possible to reject that they could belong to LF8 in relation with their size and shape.

The extraction blank remained empty of typical collagen peptides, indicating that lab contamination did not influence any taxonomic identification for these 17 bone specimens. Two bone specimens could not be identified taxonomically. One bone specimen was identified as a hominin, one bone specimen as a bear (family Ursidae; *Ursus* sp.), and the remaining 13 bone specimens were all identified as *Bos*/*Bison*, consistent with *Bos*/*Bison* fragments being, in general, the most abundant determinable faunal specimens from the LF8 context (*SI Appendix*, Table S8). The identification of a bear constitutes the first *Ursus* specimen identified from the Mousterian units, squares 1 and 2. Fortunately, the hominin specimen (R-EVA 3336) was large enough to be directly AMS radiocarbon dated.

These datasets also allowed for a comparison with the specimens from layer L2Bj that were analyzed with ZooMS. Deamidation values for samples originating from L2bj (in green) and Bos/Bison samples spatially associated with LF8, including the hominin identified through ZooMS (both in red), are indistinguishable from each other (*SI Appendix*, Fig. S15). The only exception concerns the bear specimen which has deamidation parameters remarkably different, in that it is significantly less deamidated (in this case, values are closer to 1 for both axes, *SI Appendix*, Fig. S15). In other words, the bear specimen likely had a different diagenetic history. This ursid remain has yielded an age of 39.7-38.6 ka cal BP (95.4%) (R-EVA 3340, *SI Appendix*, Table S1).

Analysis of ancient mitochondrial DNA

Despite successful DNA extraction and library preparation (*SI Appendix*, Table S3) and repeated sampling, less than 6,000 unique hominin mtDNA fragments were recovered from the specimen (*SI Appendix*, Table S4), indicating relatively poor DNA preservation that prevents reconstruction of a complete mitochondrial genome sequence. However, between 15 and 30% of the

fragments matched the Neandertal state at positions that distinguish Neandertal from present-day human mtDNA genomes (*SI Appendix*, Table S5). This number increases to 97% or more if only deaminated fragments are considered, showing that the specimen contains highly deaminated Neandertal mtDNA in addition to present-day human contamination. We thus firmly attribute the specimen, at least mitochondrially, to Neandertals. All the identified human bones in the excavated area belong to LF8, and we consider that the most parsimonious attribution of the fragment identified as hominin by ZooMS and further identified as a Neandertal using mitochondrial DNA belonged to LF8.

Taphonomic analysis

Taxonomic spectrum

The studied material includes all the faunal and human elements coming from the 1970 and 1973 excavations in the Mousterian layers named M2 and M3 (squares 1 and 2). That includes the LF8 Neandertal child and the associated fauna (small and large mammals). A total of 4,609 remains have been studied, including 191 elements belonging to the LF8 child (*SI Appendix*, Table S9). Among the fauna, 127 elements are identified, among which only 22 were taxonomically identifiable, which represents only 2.9% of the NR. The presence of a huge number (NR = 3,492) of indeterminate small burnt bone fragments (less than 25 mm long) explains this low rate (see below). One small fragment of coprolite completes the series.

Human remains represent the main part of the NISP. Among the identified fauna, large bovids and horse dominate the spectrum, followed by red deer and reindeer (*SI Appendix*, Table S8). The La Ferrassie large bovid remains from the 1968-1973 excavations were classified by Delpech (70) as either *Bos primigenius*, *Bison priscus*, *Bison schoetensacki*? or *Bison* sp. Those corresponding to levels L2bj to L10 in the front excavation plus M1 and M2 in the sagittal stratigraphic profile were identified as indeterminate Bovines. More recently, the large bovids identified by S. Madelaine in the Mousterian levels belong to *Bison* sp. (10). In our sample, the taxonomic attribution of a horn to *Bison* sp. is based on its general straight morphology (versus the more curved and twisted horns of *Bos primigenius*) and the presence of well-marked crests (71). Nevertheless, despite the apparent absence of aurochs at La Ferrassie, we will use here the general term of large bovids.

Finally, ZooMS analyses (see above) on 17 morphologically unidentifiable fragments provided the following taxonomic results: one hominin, one *Ursus* sp., and 13 *Bos*/*Bison* fragments and two unidentified fragments.

Anatomical representation

The anatomical representation of the LF8 child is limited to the cranium, vertebral column, thorax and pelvis. For the appendicular skeletal, only four hand phalanges are present (*SI Appendix*, Table S10) and no long bones of the limbs were recovered (1, 7).

Bovines are mostly represented by head elements, including skull and mandible fragments. One proximal shaft fragment of a right ulna is the only post-cranial element. Skull fragments count one portion of the lacrymal bone (anterior side of the orbit), one part of the frontal bone (upper side of the orbit, possibly left part) and an almost complete bison horn-core (left side?). Five fragments of a right hemi-mandible were recorded. Two of them refitted on the basis of green bone fractures (numbers 464 and 488 from the layer M2). The nearly complete horn-core is composed of twenty-five broken elements that could be refitted in three main fragments, themselves permitting to virtually reconstruct the whole remain. We also note that nearly the fragments that were used for ZooMS analyses are from *Bos*/*Bison*.

Horse (*Equus ferus*) is represented mainly by fetal remains, including three metapodials and one tibia. Other elements include one proximal shaft fragment of a right ulna, one proximal portion of a scapula and one enamel fragment of an indeterminate cheek tooth.

Red deer (*Cervus elaphus*) is present with two metacarpal fragments and one enamel fragment of an indeterminate cheek tooth. Reindeer (*Rangifer tarandus*) is represented by one incisive (R I/2 D) of a young adult. Additionally, two distal fragments of a humerus and a femur are classified as indeterminate cervids.

Lastly, apart from those securely attributed to horses, there are additional (NR = 14) fetal remains of other ungulates, including radius, tibiae, ribs, vertebrae and indeterminate shaft fragments.

Bone distribution and fragmentation

The spatial distribution of the human elements suggests that most of them were in anatomical congruence. On the opposite, animal bones were found dispersed throughout the layer (*SI Appendix*, Fig. S16). Only the two fragments of the large bovids mandible recovered in the layer M2, refitted on ancient breakage edges.

The faunal and human series show significant differences in the degree and type of bone fragmentation. While the human sample yields mainly complete or almost complete remains (NR=33, 19% of the bone elements; *SI Appendix*, Tables S5 and S7) with a few complete isolated teeth, the fauna is only represented by teeth or bone fragments. The only complete faunal elements are 12 fetal or neonate remains and two ossified costal cartilage. Moreover, the very low identification index for the animal sample (2.9%) confirms its high degree of fragmentation (*SI Appendix*, Table S9).

The sorting of the fragments by size classes is nevertheless almost the same, including a majority of fragments less than 25 mm long for each series, with some large fragments found among the animal bones, especially bison horn fragments (*SI Appendix*, Table S11). However, although the LF8 human represents a two-year-old child skeletal consisting of small anatomical elements, their low degree of fragmentation and bone destruction provided some large fragments overlapping with those of the middle-size and large-size adult ungulates.

About the causes of fragmentation, we recorded various types of fractures for both series. Among the LF8 human remains, many fragments display alterations recorded as pits, punctures, peeling, crushing or scooping out. Unfortunately, most of them are associated with recent surface removals and/or present recent breakages of their edges, which make them difficult to interpret. The fragility of these immature human bones, having mainly cancellous tissue and thin cortical, and possibly their excavation and/or curation conditions, could partly explain these indeterminate and recent damages, respectively 97.9% and 70.2% for the fractures and 59.2% and 67.8% for the surface alterations (*SI Appendix*, Table S12). No clear green bone breakage is attested.

Regarding the faunal material, the situation is different. The sample is dominated by middle-size or large-size ungulates shaft fragments whose fracturation causes are much easier to identify. Green bone fractures are present for 13.7% of the NR and a third of them present percussion marks (*SI Appendix*, Table S12). Post-depositional and recent breakages are present with similarly low rates, around 5% of the NR, and indeterminate fractures represent only 15.2% of the sample. Among the recorded ungulate shaft fragments, the quasi totality belongs to the first size and circumference categories L1 and C1 (*SI Appendix*, Table S13), which is characteristic of marrow removal processed by humans (43). Excepting fetal remains, no animal bone cylinders were found. The absence of limb elements in the human skeletal prevents any comparison of the long bone breakage features between the human and animal samples.

Bone preservation and surface modifications

The bone destruction indexes and surface illegibility rates indicate a good state of bone preservation for the two series (*SI Appendix*, Table S12). Nevertheless, some types of alteration statistically differ between both faunal and human bones. Cracking, desquamation and smooth edges, all related to the time of surface bone exposure (weathering modifications), have higher rates among the human sample, while still limited to a low degree of alteration (first stages). On the opposite, edaphic alterations, such as root etchings, concretions, chemical corrosion and black colorations (possibly manganese oxides or organic deposits), correlated to the humidity and chemical action of the sediments (post-burial modifications), highlight similar rates, mostly non or slightly significantly different. Root etchings and concretions are very scarce for the two samples, whereas the first stages of chemical corrosion and colorations reach more than half of the bone surfaces.

A total of 18 faunal remains, representing 14.9% of the NR, were chewed by carnivores (*SI Appendix*, Table S12). No human bone was affected by this modification. Pits, punctures and scores are present on all taxa, except on the red deer remains, which are very scarce (NISP=3). Measurements of the pits on cortical bone are provided (*SI Appendix*, Fig. S17). Based on the criteria defined by Domínguez-Rodrigo and Piqueras (72), it argues for the main action of a middle-size or a small carnivore such as a canid. However, the presence of a few pits of greater dimension and the overlapping of the means and standard deviations with those of hyenas, lions and bears cannot rule out an occasional action of a larger carnivore (*SI Appendix*, Fig. S18). Regarding the faunal remains from all the Mousterian levels at La Ferrassie, carnivores are rare and are represented by hyenas, bears, wolves and foxes (Madelaine, in (10)).

No cut-marks, percussion marks or even fire traces are recorded among the human bones (*SI Appendix*, Table S12). On the opposite, all these marks are present on the animal sample, highlighting butchering activities for all animal taxa.

Two refitted fragments of a large bovid mandible present numerous scraping marks associated with percussion pits and a large bovid ulna has some skinning or filleting cutting marks on its proximal and posterior shaft portion. The latter is also associated with incipient percussion pits. Nearly fifteen incisions were found on a red deer metacarpal shaft fragment indicating skinning activities. A distal fragment of a cervid humerus shows a dozen small and thin incisions on the lateral epicondyle, which could be interpreted as disarticulation marks and a cortical negative flake on the medial epicondyle resulting of hammerstone breakage. A horse ulna shows on its posterior side and proximal portion a percussion notch that can also result of marrow removal or disarticulation processing.

There are six cut-marked rib specimens belonging to indeterminate middle-size ungulates. Many of these are small fragments whose location (dorsal or ventral side) is difficult to identify. However, scraping marks and incisions were recorded, indicating possibly both defleshing and evisceration. Additionally, three of them have percussion notches and pits. Finally, four indeterminate skull fragments have scraping marks and incisions, indicating periosteum removal and/or skinning.

The 24 other cut-marked specimens are indeterminate fragments, mostly long bone shaft fragments with incisions showing meat removal activities. The marrow removal activity is also supported by the presence of percussion notches, cortical flakes and pits on 16 long bone indeterminate fragments.

Additionally, ten specimens, among which there are two ribs and one skull fragments, show some scattered percussion pits or cupules mostly connected with sliding striations that could be related either to marrow removal (incipient notches) or lithic production activities (bone retouchers). The lack of easily circumscribed and identified used area argues rather for the first possibility.

Fire traces are only found among the faunal sample, as the Mousterian layer M2 provided thousands of small burnt bones. Only 17 belong to the layer M3. All represent 79.1% of the totality of the faunal remains (*SI Appendix*, Table S14). Among the burnt elements, most are smaller than 25 mm (99.4%), a great majority (81.7%) is trabecular and 97.7% have reached the carbonization stage. Compared to experimental data (73), these three percentages indicate that the burnt sample can be correlated to a common use of bone as fuel. One identified specimen, a distal shaft fragment of a cervid femur, show fire traces on its extremity (grey-black coloration). The fire exposure of articular portions could indicate the roasting of some meat quarters.

All the characteristics of the faunal sample, spectrum as taphonomic modifications, fit well with the one coming from the new excavations carried out at La Ferrassie (Madelaine and Steele, in (10)). Among Mousterian layers, the same types of ungulates, supplemented with a few remains of roe deer and rhinoceros, and a single fox remain was identified. Severe alterations leading to the illegibility of the bone surfaces concern only a small part of the sample (< 20%). Carnivore and human modifications are also present with comparable rates to those of LF8, indicating humans as the main accumulators. Likewise, burnt bones are small fragments coming from sieving (< 25 mm). They are also numerous, although in smaller quantities than those associated with LF8. Given all these similarities, the possibility that both faunal samples were connected in the past and that a part was used to fill the LF8 burial may not be excluded.

Lithics

There is no clear difference in term of technology, state of preservation and constitution between the two restricted samples of lithic elements coming from the layers M2 and L2B-L2Bj in square 1. It should be noted that these two samples are very limited in size. Comparisons with assemblages from other areas of the La Ferrassie site will be done in the future, once the material discovered during the 2010-2015 period is fully studied and published, because during the excavations of the early 20th century only a small amount of material was recovered.

Geology

A sample of loose sediment taken from the area of the LF8 specimen was consolidated and processed into a 60 x 90 mm thin section. This sample was selected from a bag of sediment that was the result of a sieving event done in square 1, layer M2 in 1973. As this was an artificially consolidated sample, the best we hoped for is some observations on the general makeup of the constituents and perhaps find clues that might be more diagnostic about the history of the deposit. Compositionally, the sieved sediments overwhelmingly consist of various-sized grains of limestone and quartz, along with some burnt bone fragments. This composition matches that of many of the sediments from the LF8 area in general, including the burned bone grains, which are scattered throughout the LF8 sedimentary column. In addition, present in the thin section are limestone grains and aggregates coated with reddish brown silt clay, which are also present in other thin sections. Interesting, however, is the occurrence of a grain with the remnant of a relatively thick coating of reddish brown clay. Similar types of coatings were observed especially in thin section LAF-PG-408-1, which comes from Layer B (formerly Layer 4), near the bottom of the profile. The sedimentary nature of this sample would be consistent with the stratigraphic position of the LF8 specimen inferred from the spatial data and the photographs (*SI Appendix*, Fig. S5), although the nature of the sample does not allow further inferences.

SI Appendix, Fig. S10 shows the state of the area where was found LF8 at the end of the 2014 excavation. Unfortunately, the exact sedimentary environment of LF8 could not be studied during the recent field season because it was completely excavated in 1973. We could nevertheless identify the stratigraphy on several profiles located around the area where was discovered the child. Five layers were recognized.

Attempt of interpretation of the case of the bear bone

Among the specimens that were analyzed with ZooMS, the bear (*Ursus* sp., family Ursidae) specimen has deamidation parameters remarkably different, in that it is significantly less deamidated than all the other specimens (in this case, values are closer to 1 for both axes, *SI Appendix*, Fig. S15). In other words, the bear specimen, supposed to be from the same context as the LF8-associated specimens, has a diagenetic signature different from the other fauna remains analyzed from this area. This ursid remain has yielded an age of 39.7-38.6 ka cal BP (95.4%) (R-EVA 3340, *SI Appendix*, Table S1), younger than all the other specimens from both layers L2B and M2 and potentially explaining the lower deamidation value.

There are two alternative explanations possible for the origin and interpretation of this specimen, among which we cannot distinguish. First, the ursid specimen might indicate that the bone material spatially associated with LF8 contains material from different chronological and/or diagenetic histories. Alternatively, the inclusion of this specimen might be due to an excavation error in 1970. It might have fallen from an overlying layer from exposed excavation profiles and be incorporated as part of the M2 layer material during the last days of excavation in 1970.

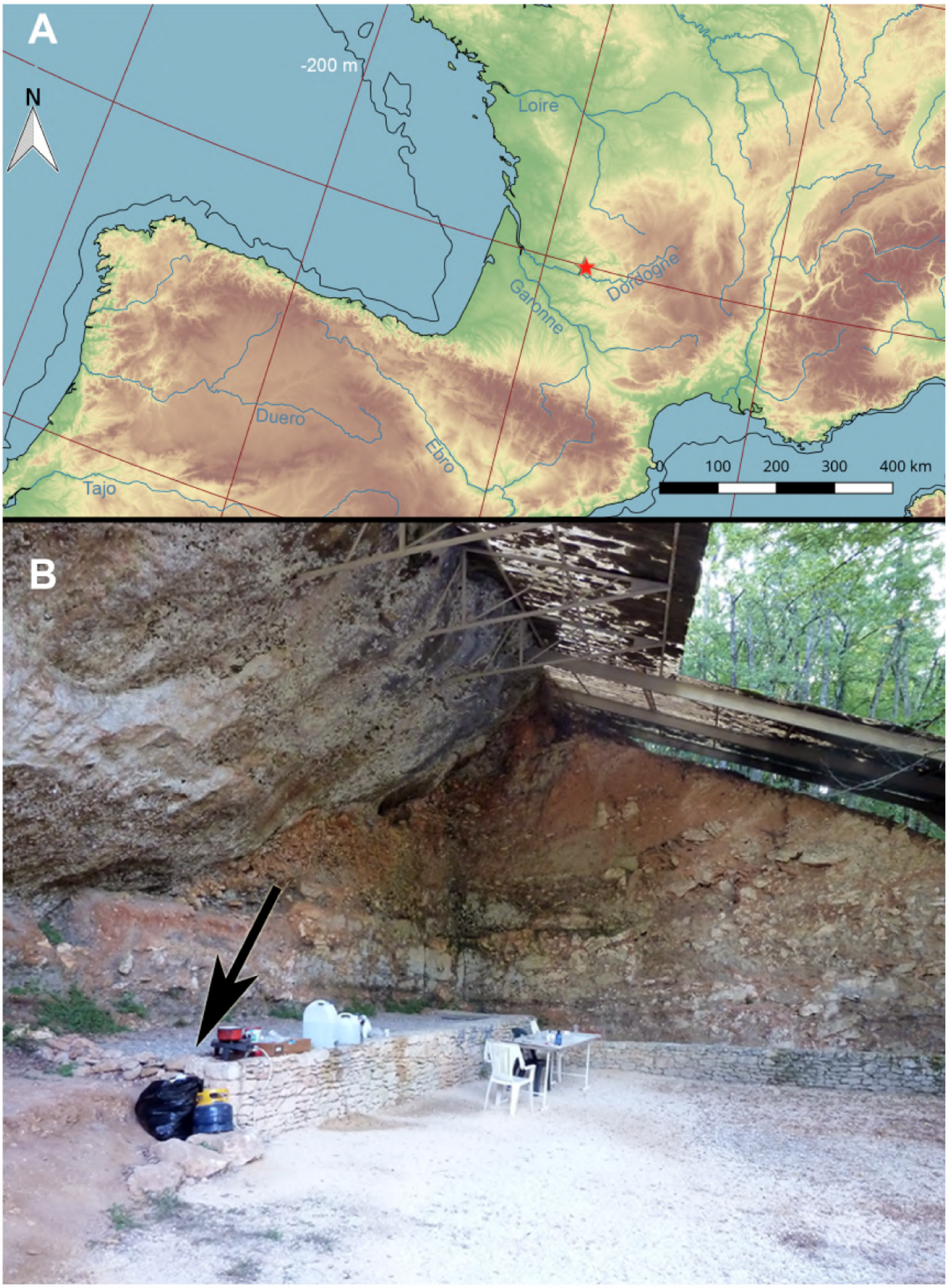

Fig. S1. Site location (A) and eastern sector of the rockshelter where the 1968-1973 excavations took place (B). The arrow indicates the approximate location where La Ferrassie 8 (LF8) was found. This photo was taken in august 2013 before the excavation in the LF8 sector. Base cartography obtained from the European Environment Agency. Rivers and bathymetry obtained from Natural Earth. Map generated with QGIS 3.14 (https://www.qgis.org) and Inkscape 0.91 (https://www.inkscape.org).

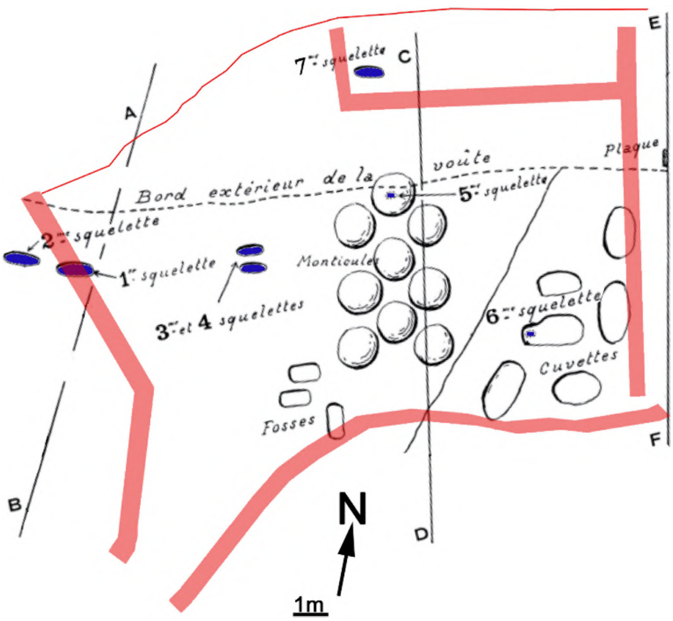

Fig. S2. Position of the seven Neandertal skeletons found at the site of La Ferrassie, Dordogne, France. La Ferrassie 8 corresponds to the label "*7ème squelette*", and its position is represented on the map after Peyrony (74) that shows the six first Neandertal individuals, relatively to actual elements on the site: the actual frontal stratigraphy shown as the thin red line and the walls built after the 1968-1973 excavations to protect the sediment and delimitate the site are shown in thick red.

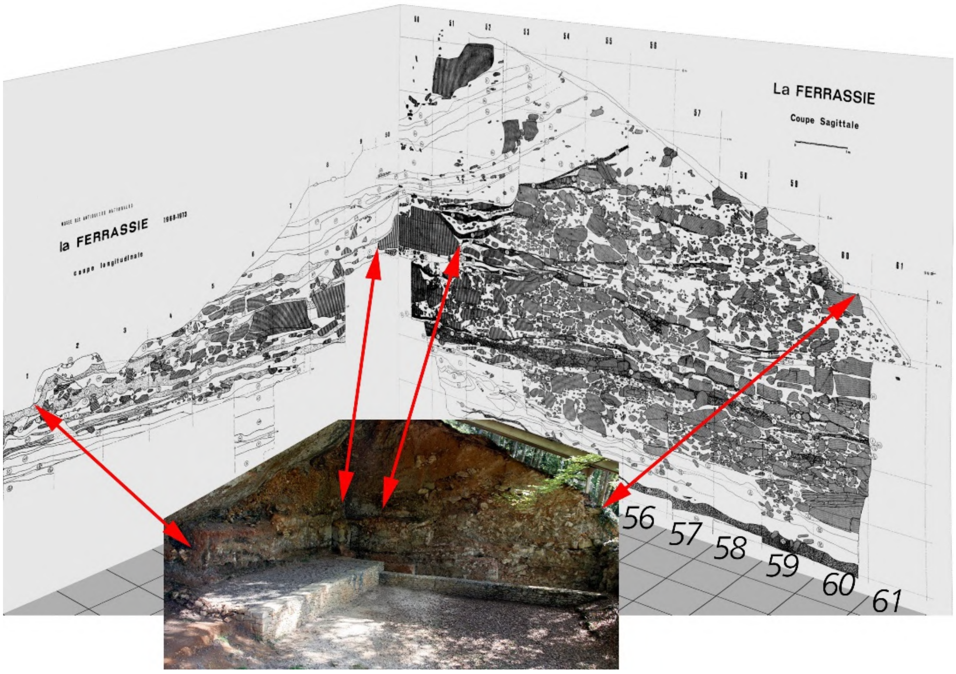

Fig. S3. Idealized sketch (based on the profiles by Laville and Tuffreau (64) which approximately locates some of the squares excavated by Delporte in 1968-1973.

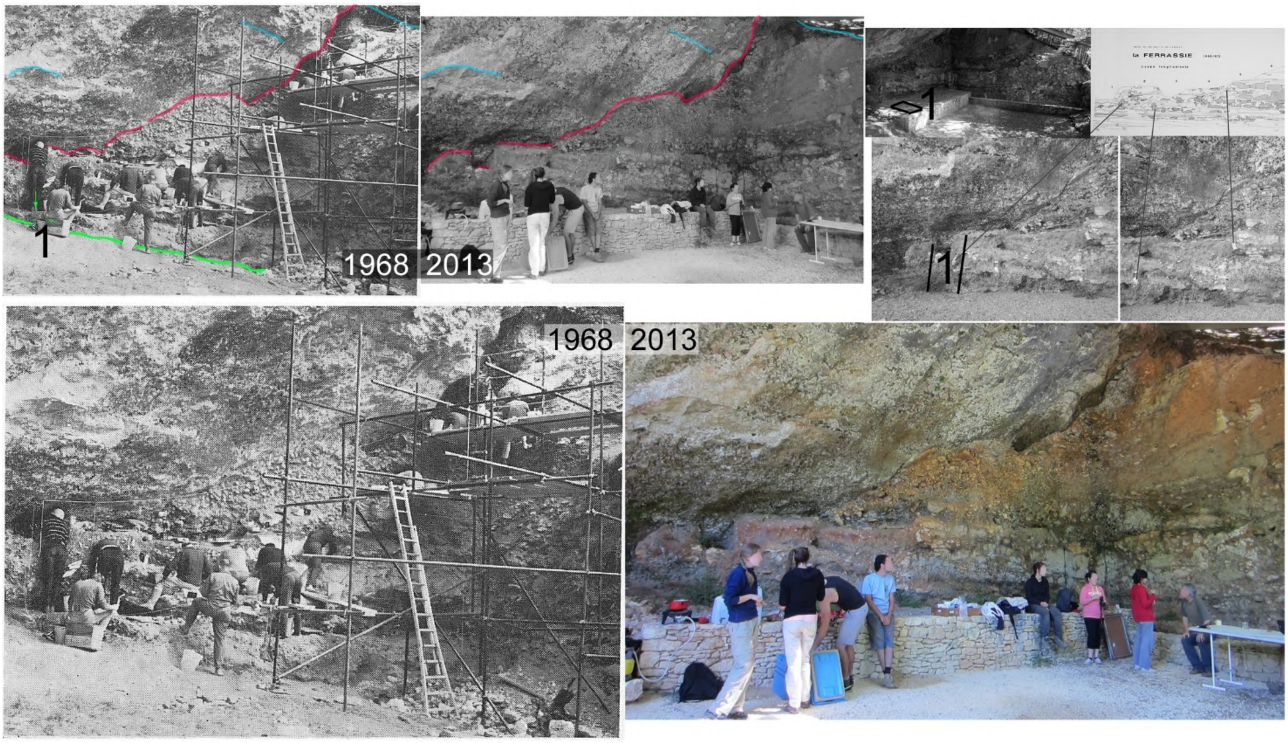

Fig. S4. Comparison of the exposed sediments at La Ferrassie between 1968 (63) and 2013. Red line: the upper extent of the frontal profile; blue line: matching details on the wall of the *abri*; green line: inferior visible limit of the exposed sediment in 1968. The green arrows and label 1 show the position of square 1 of Delporte’s excavations where LF8 was found.

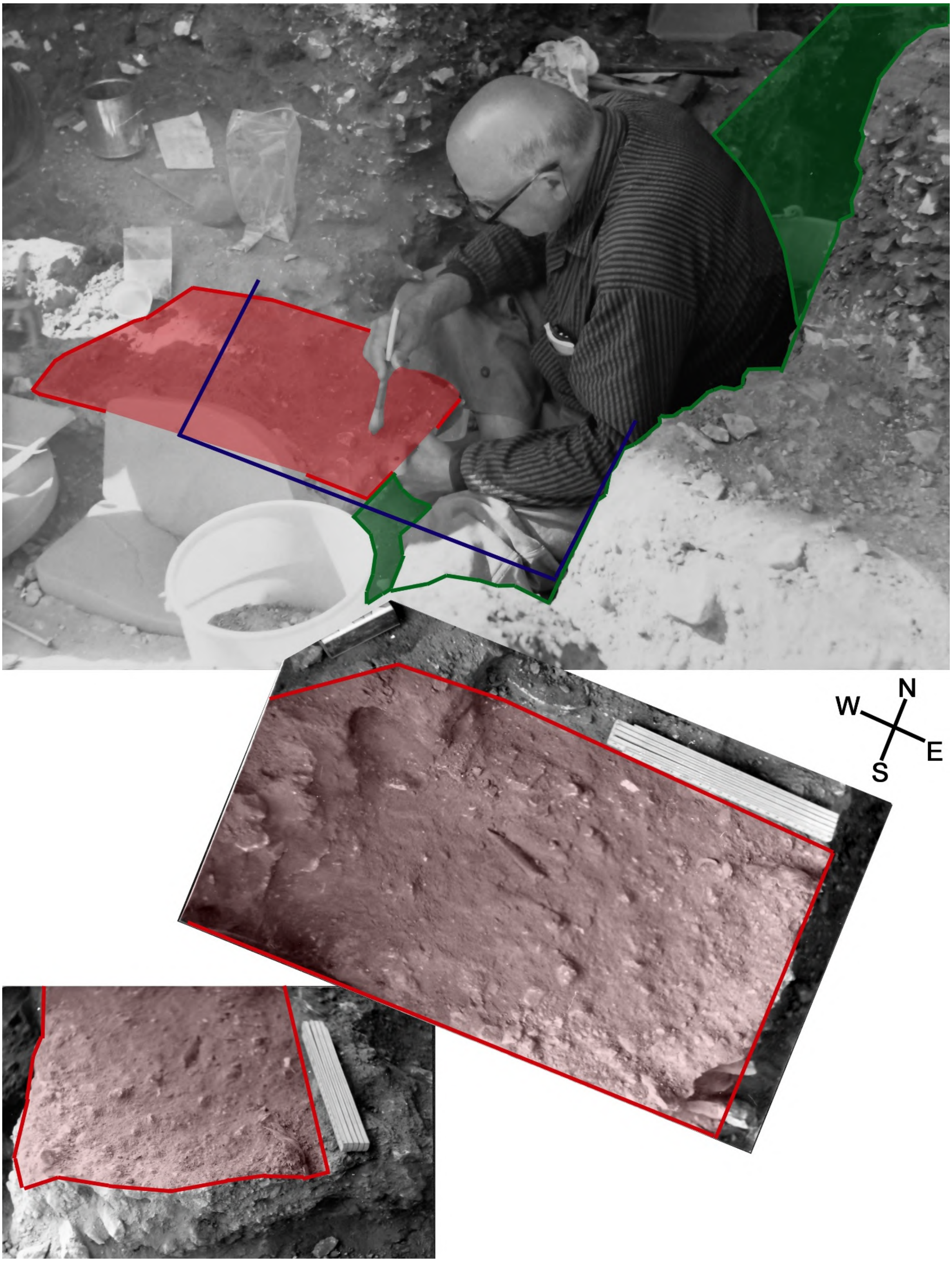

Fig. S5. General view of the excavated area of square 1 on August 27th 1973. The excavated area where the elements of LF8 were found in 1973 is in red. The blue lines border the approximate original extension of square 1. The green area corresponds to the trench excavated in 1970 where a parietal fragment and several teeth were identified. Original photos by Christophe Toupet, donated to the Musée d'Archéologie Nationale (Saint-Germain-en-Laye, France).

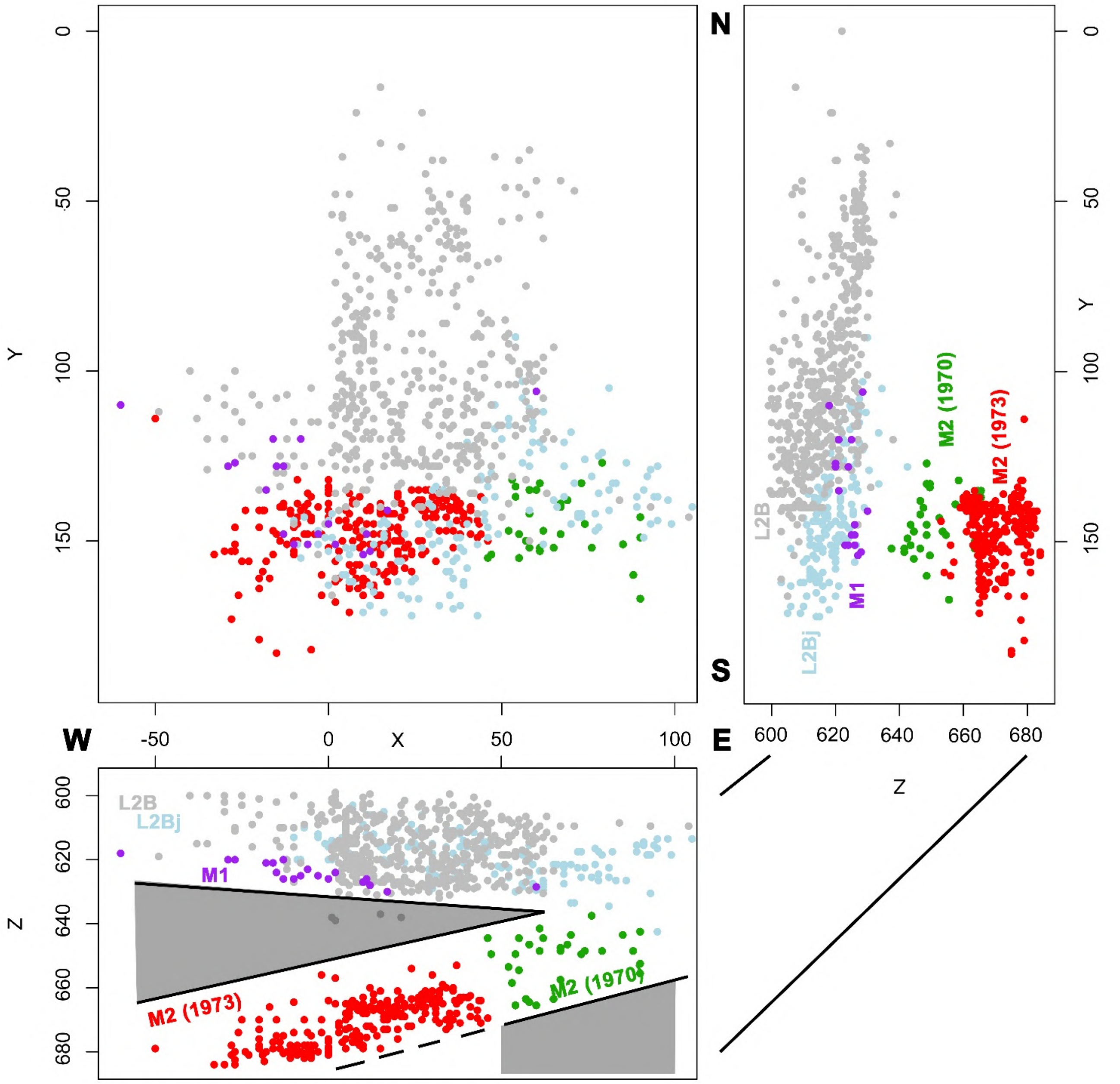

Fig. S6. 3D distribution of the finds in square 1 from layer L2 (grey), L2Bj (light blue), M1 (purple) and M2 (in red finds from 1973 and in green finds from 1970) in YZ, XY and XZ orientations. The X axis corresponds to west-east, Y to south-north and Z to the vertical extension. The grey areas on the XZ profile correspond to the excavated areas that are sterile. The new excavations performed at the site in 2014 have confirmed that below the 1973 findings, the M2 layer is sterile (see Fig. S9).

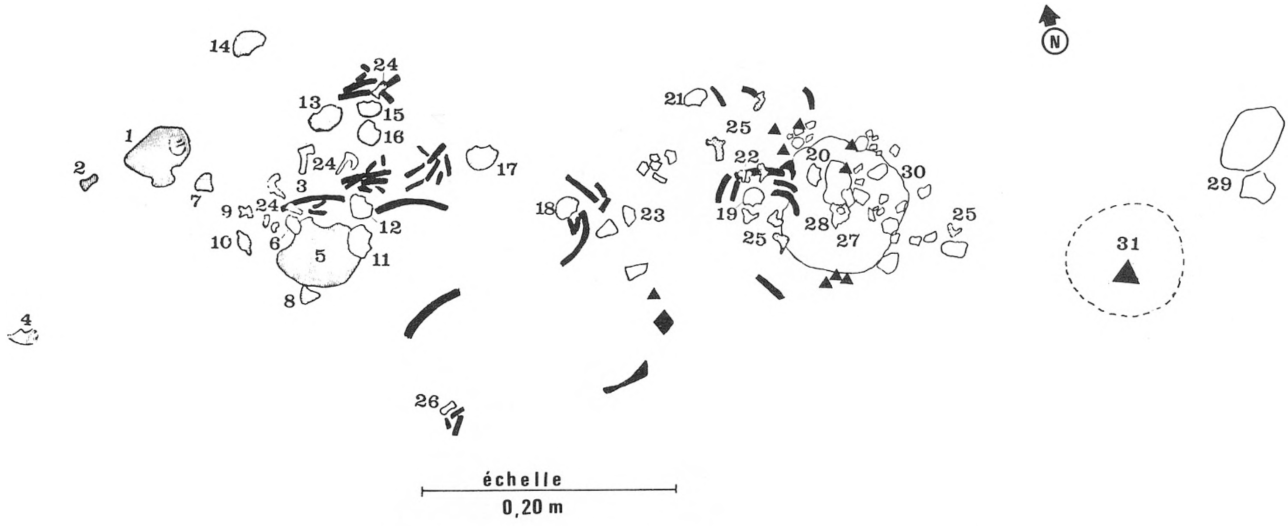

FIG. 14. — Répartition des restes humains attribués à La Ferrassie 8 (fouilles H. Delporte, 1973).
1) Ilion droit. 2) Pubis droit. 3) Pubis gauche. 4) Ischion gauche. 5) Ilion gauche. 6) Première vertèbre sacrée (point central). 7) et 8) Ailerons sacrés droit et gauche (points costaux). 9) Aileron sacré droit (point neural). 10) 2e ou 3e vertèbre sacrée (point central). 11) L5. 12) L4. 13) L3. 14). L2. 15) L1. 16) T12. 17) T11. 18) Vertèbres thoracique moyenne. 19) Vertèbre thoracique supérieure. 20) Vertèbre thoracique supérieure ou cervicale inférieure. 21) Vertèbre cervicale inférieure. 22) Apophyse odontoïde de l'axis. 23) Corps de l'axis. 24) Arcs vertébraux lombaires. 25) Arcs vertébraux thoraciques et cervicaux. 26) Phalange. 27) Occipital. 28) Os wormiens. 29) Restes pariétaux. 30) Fragments du crâne cérébral. 31) 10 dents (tamisage 1970)

Fig. S7. Heim's figure 14 (1), showing a general XY view of the LF8 elements. There are errors in the positions of the occipital and the coxal bones. The occipital was found in a vertical position and not horizontal as represented in this figure. The archives also contain a small sketch of the orientation of the iliac bones of the pelvis that shows that the acetabular part of both bones was oriented towards the east. The isolated rib east of number 26 has now been identified as a faunal remain. Labels: 1. Right ilium; 2. Right pubis; 3. Left pubis; 4. Left ischium; 5. Left ilium; 6. First sacral vertebra (centrum); 7 and 8. First sacral vertebra (right and left lateral elements); 9. First sacral vertebra (right neural arch); 10. Second and third sacral vertebrae; 11. L5; 12. L4; 13. L3; 14. L2; 15. L1; 16. T12; 17. T11; 18. Middle thoracic vertebra; 19. Upper thoracic vertebra; 20. Upper thoracic vertebra or lower cervical vertebra; 21. Lower cervical vertebra; 22. Axis (dens); 23. Axis (centrum); 24. Lumbar neural archs; 25. Cervical and thoracic neural archs; 26. Phalanx; 27. Occipital bone; 28. Wormian bone; 29. Parietal remains; 30. Fragments of neuro-cranium; 31. 10 teeth (screening performed in 1970).

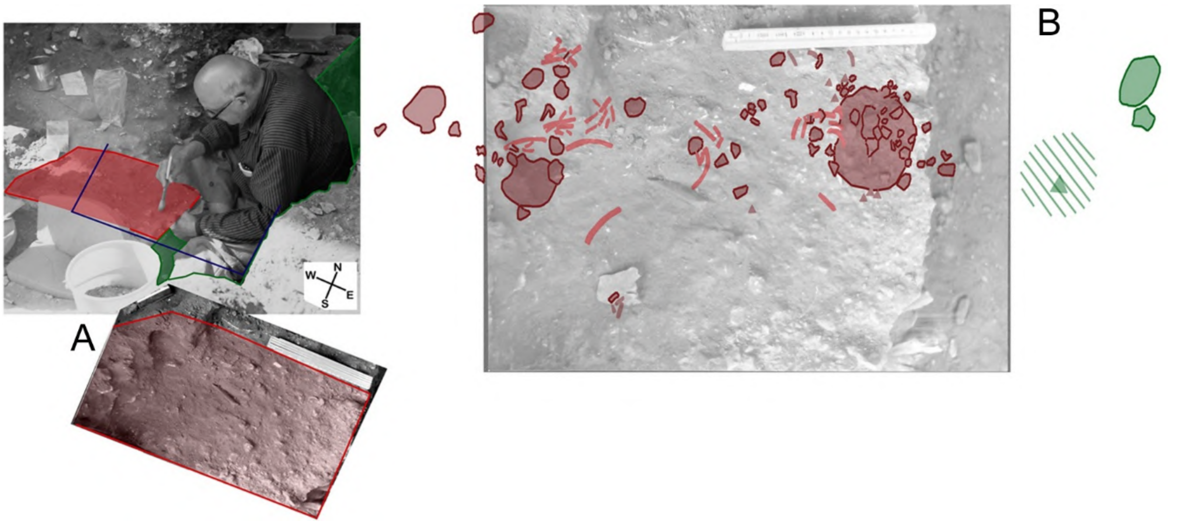

Fig. S8. Superior view of a part of the excavated area of square 1 (on August 27th 1973). In B, the elements in red are those represented on the map by Heim (1) (Fig. S6) that come from the 1973 excavation, and the two areas in green on the right correspond to the two parietal fragments and the screened area where seven teeth were found in 1970. Original photos by Christophe Toupet, donated to the Musée d'Archéologie Nationale (Saint-Germain-en-Laye, France).

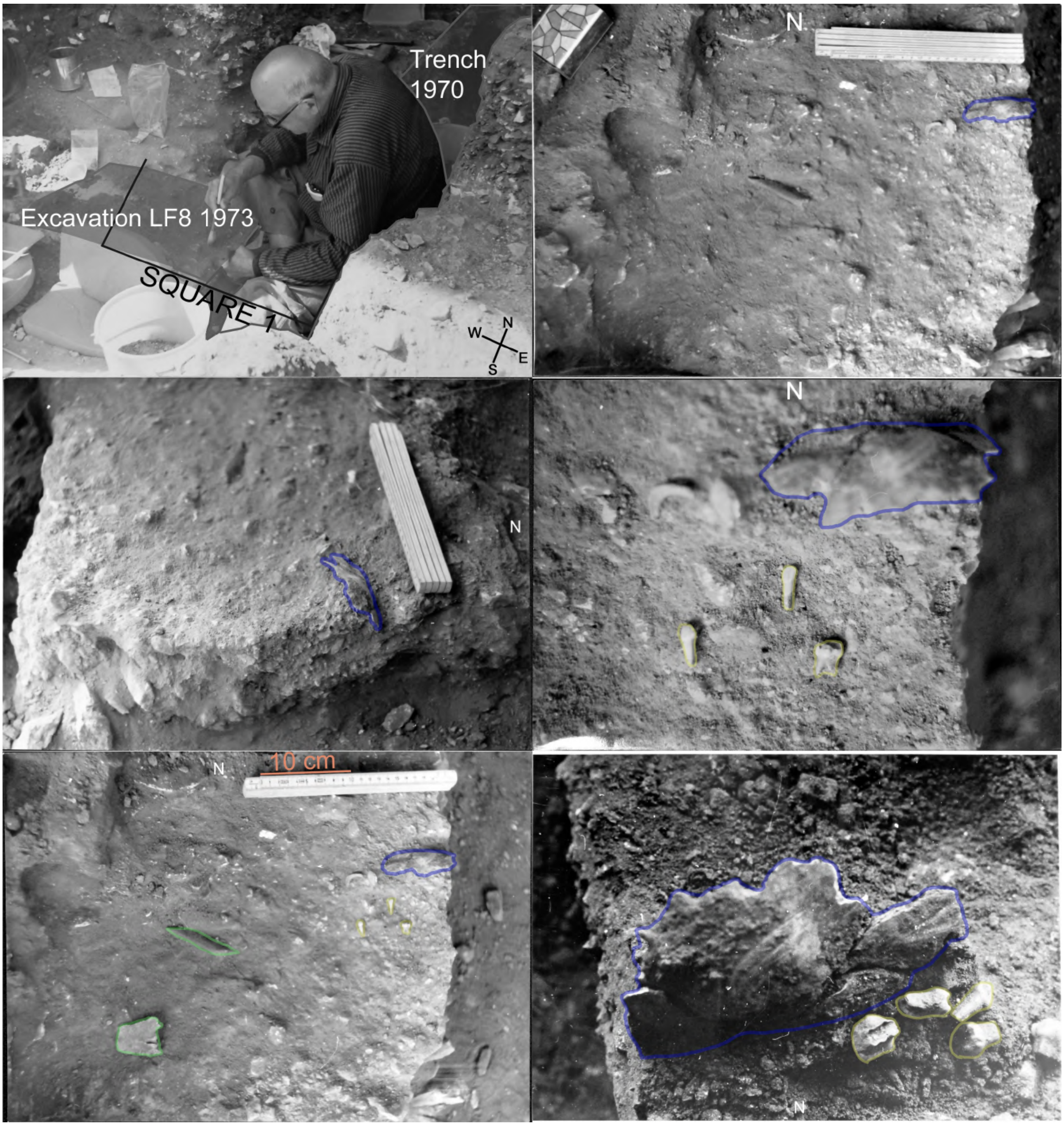

Fig. S9. The only six available photographs of the discovery of La Ferrassie 8 child, all were taken on 27th August 1973 by Christophe Toupet. The north is indicated on all the photographs, a scale is also visible on most of them. The extension of square 1 and of the excavated area in 1973, as well as the extension of the trench of 1970, are visible on the top-left image. The occipital bone is highlighted in blue, human teeth are in yellow (they are not in their original disposition on any of the photographs) and faunal remains that were recognized in the brown box in the MNHN are in green. Original photos by Christophe Toupet, donated to the Musée d'Archéologie Nationale (Saint-Germain-en-Laye, France).

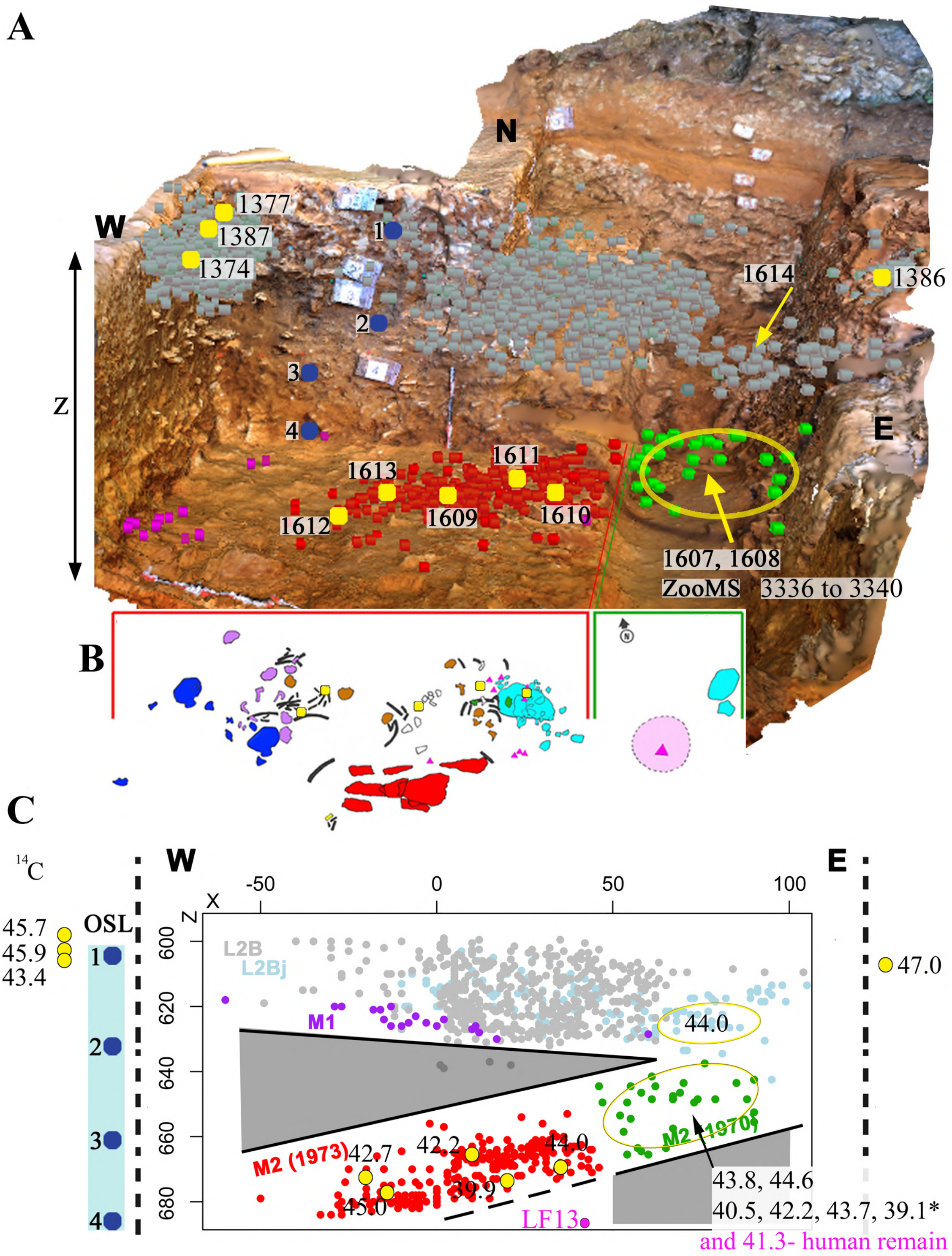

Fig. S10. Sampling strategy for luminescence dating, radiocarbon chronology and ZooMS analysis. All the analysed samples are localized relatively to all the other archaeo-paleontological remains into a 3D view of square one at the end of the excavations in 2014 (A) and also relatively to their WE (X) coordinates and elevation (Z) as deduced from the notebooks of the 1970 and 1973 excavations and conversion of the data of the 2014 excavations in the same referential (C). The drawing (B) is reworked from Heim (1), *SI Appendix*, Fig. S7, and from the original map drawn during the excavations and found at the MAN. It shows the position of the human remains at the time of the discovery in 1973 with from west to east: elements in dark blue: right ilium, right pubis, left pubis, left ischium, left ilium; elements in purple are lumbar vertebras: first sacral vertebra (centrum), first sacral vertebra (right and left lateral elements), first sacral vertebra (right neural arch), second and third sacral vertebrae, L5, L4, L3, L2, L1; elements in brown are thoracic

vertebras: T12, T11, middle thoracic vertebras, upper thoracic vertebra, upper thoracic vertebra or lower cervical vertebra; elements in green are: lower cervical vertebra, axis (dens), axis (centrum), lumbar neural arches, cervical and thoracic neural archs; elements in black are costal remains; elements in light blue are cranial elements, including the large occipital bone; triangles are teeth) while the elements in red correspond to the fragment of the bison horn-core. Analysed samples: Blue dots (with labels from 1 to 4): samples for luminescence dating, coming from the northern profile of square 1 (and in C result obtained at the level of the L2B-L2Bj complex and at the same depth than the LF8 child) and approximate vertical distribution relatively to the archaeo-paleontological materials found in the Mousterian layer in 1970 (green dots) and in 1973 (red dots), as well as those of the overlying layer (grey dots) and the few elements that were found in 2014 out of secure stratigraphic context in a dirt layer (pink dots, including the new hominin tooth, LF13); yellow dots and yellow arrows: provenance of the samples for radiocarbon chronology and for ZooMS analysis (noted ZooMS, including the four elements dated posteriorly to these analysis and particularly to the identification of one hominin bone); associated with their labels that correspond to the R-EVA codes in A, and the mean values of the range for the dates cal BP (95.4) (Table 1; *SI Appendix*, Table S1) obtained on different samples from the different layers in C. * indicates the date for the bear bone that is probably not from this layer.

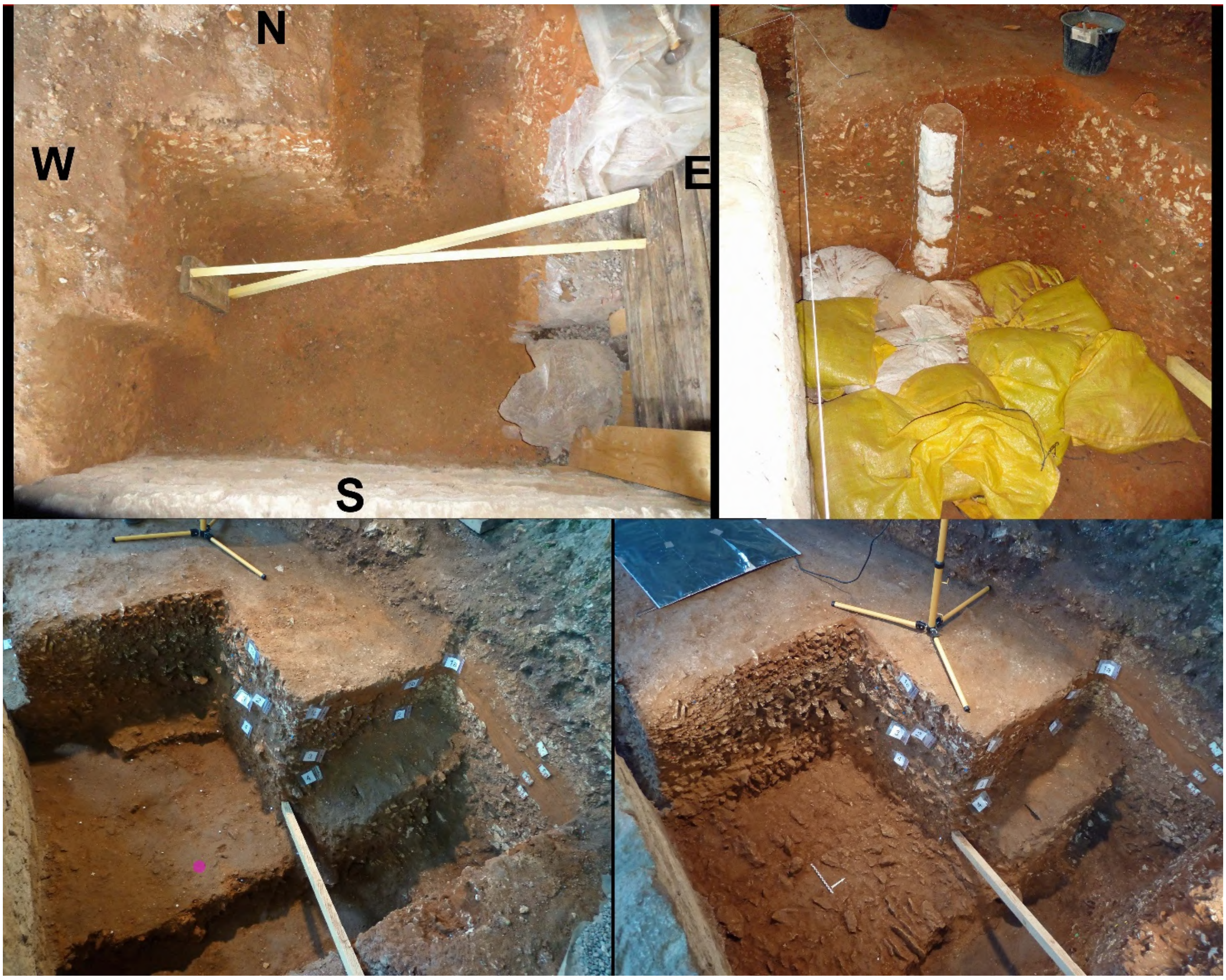

Fig. S11. Evolution of the aspect of square 1 during the excavations in 2014: original state after removing of the artificial filling left in 1973 (top left view), July 10th; excavation and sampling of the small sedimentary bank left on the western wall of square 1 (top-right view), July 27th; view of square one just before the excavations, August 4th, and position of LF13 within the dirt layer; view of square one after the excavations, August 14th.

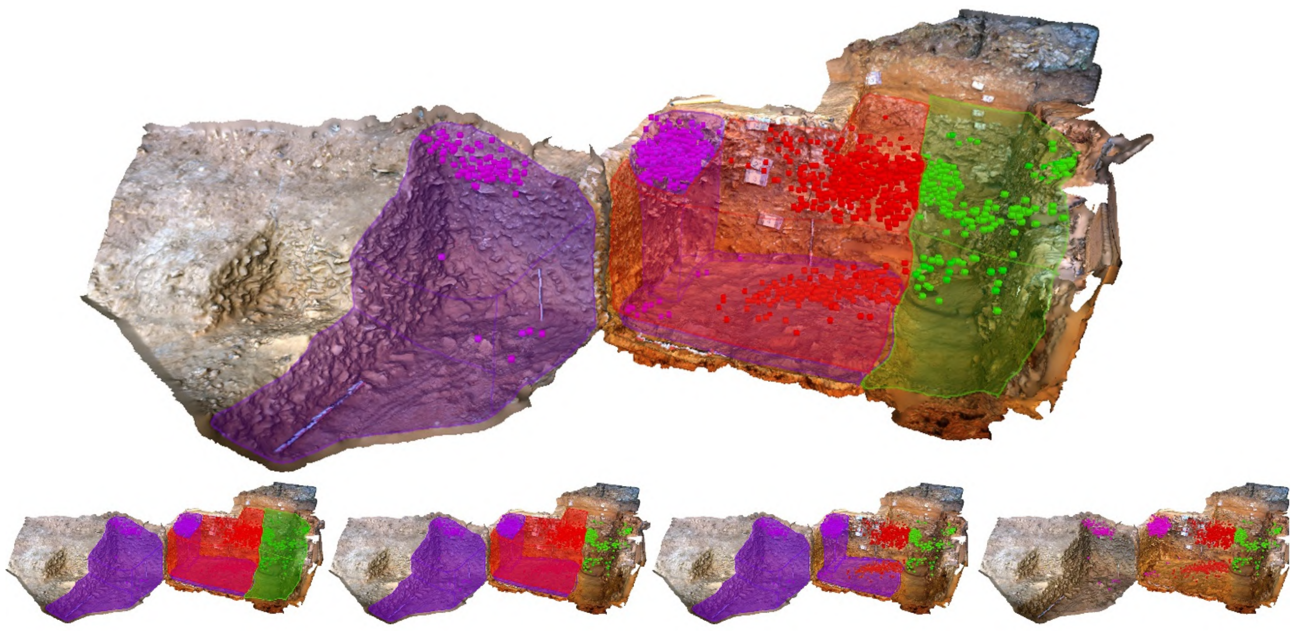

Fig. S12. 3D visualization of the sedimentary filling excavated during the different campaigns, the green area and dots correspond to the area excavated and the objects found in 1970, red is for 1973 and purple is for 2014. The bottom lines illustrates from left to right the filling of the area before 1970, at the end of the 1970 campaign, at the end of the 1973 campaign and at the end of the 2014 campaign as well as all the objects coordinated during each campaign and by extension the sterile areas.

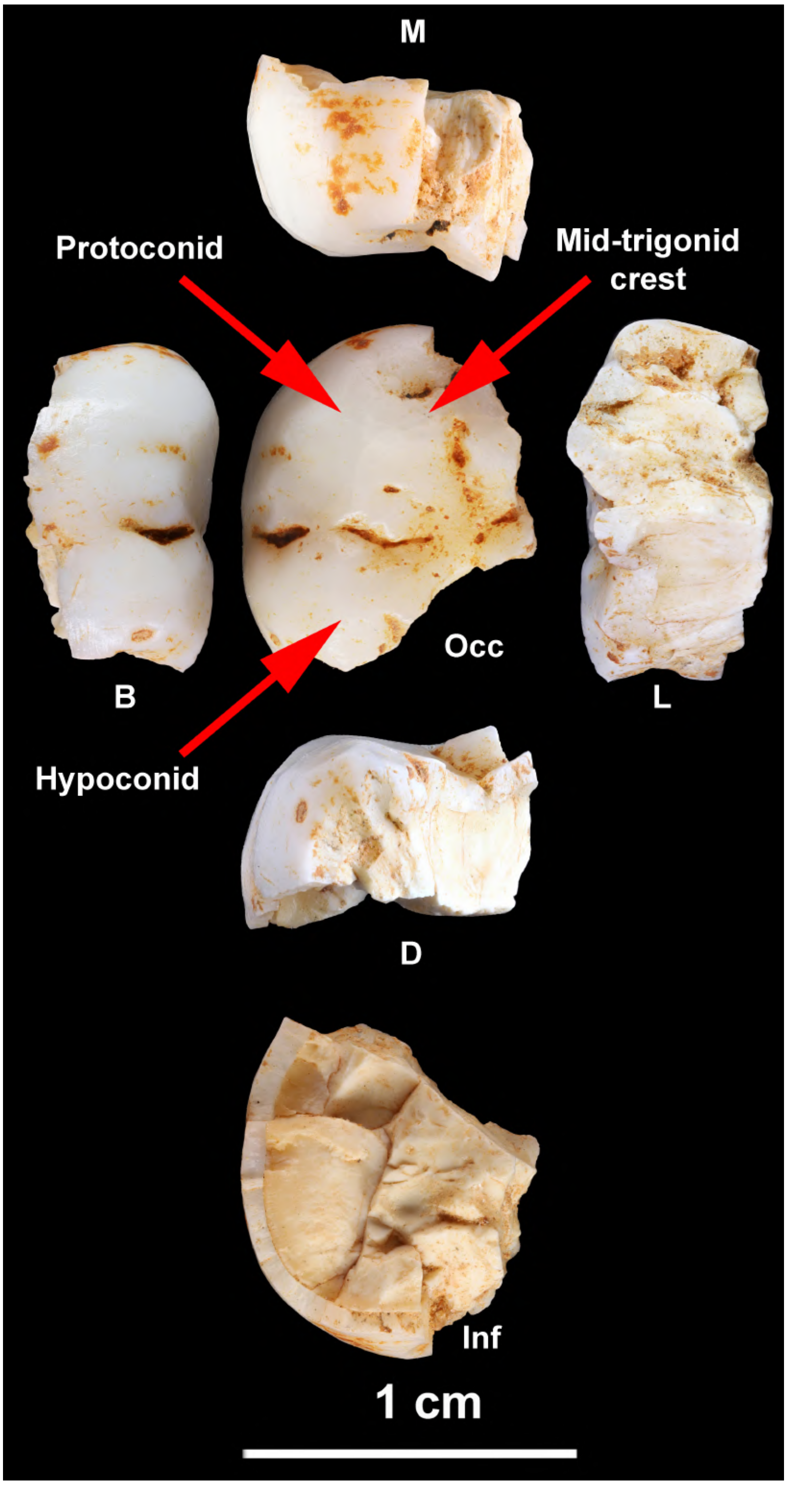

Fig. S13. Mesial (M), occlusal (Occ), buccal (B), lingual (L), distal (D) and inferior (Inf) views of La Ferrassie 13 (LF13), the new hominin specimen discovered during the 2014 excavations. This specimen represents a lower left molar, whose exact anatomical determination is elusive (Photos M. Bessou, Université de Bordeaux, UMR PACEA).

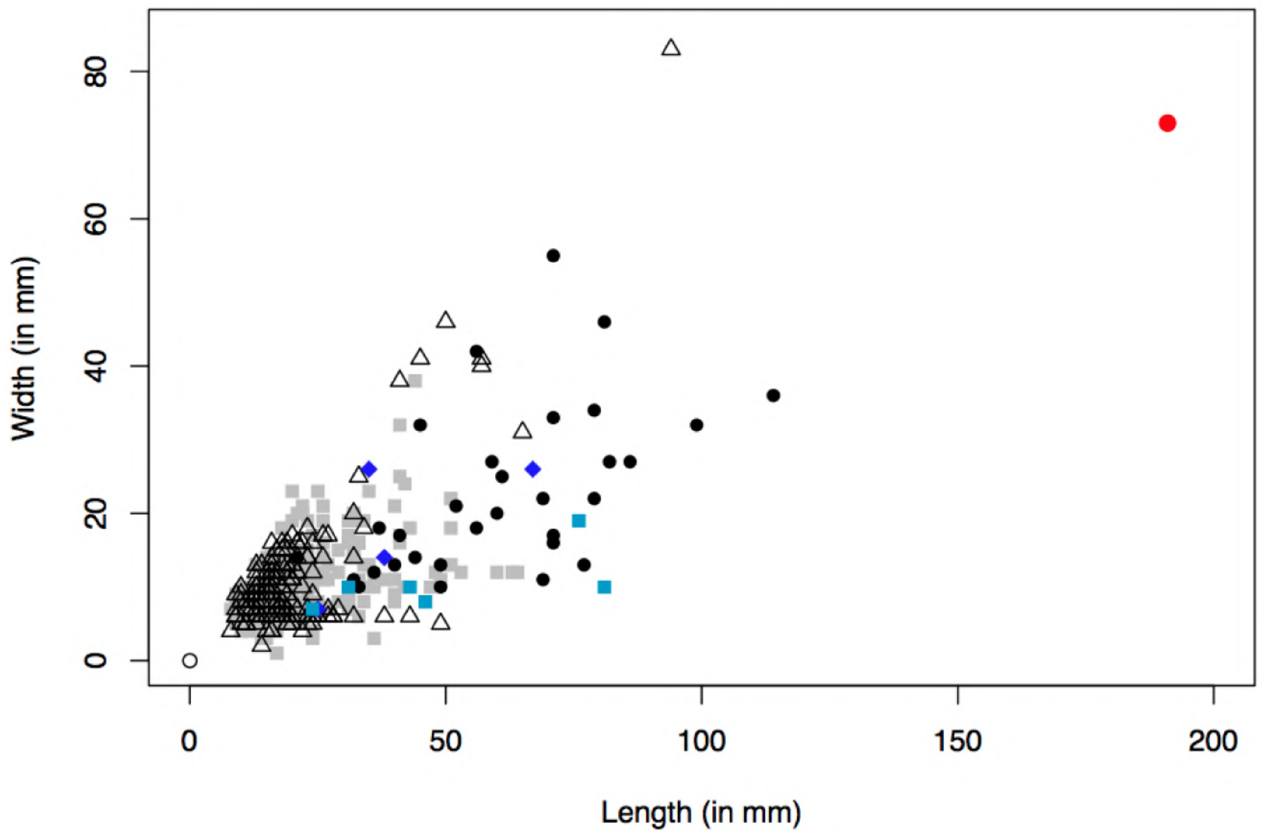

Fig. S14. Bone elements distribution by size (length and breadth) and taxa (red circle: *Bison* horn-core (refitted), triangles: *Homo*, black circles: *Bison* (including all isolated horn fragments), blue squares: *Equus*, purple diamonds: *Cervus*/*Rangifer*). The horn fragment (after the refittings, red circle) has been plotted and is outside the range of the collection.

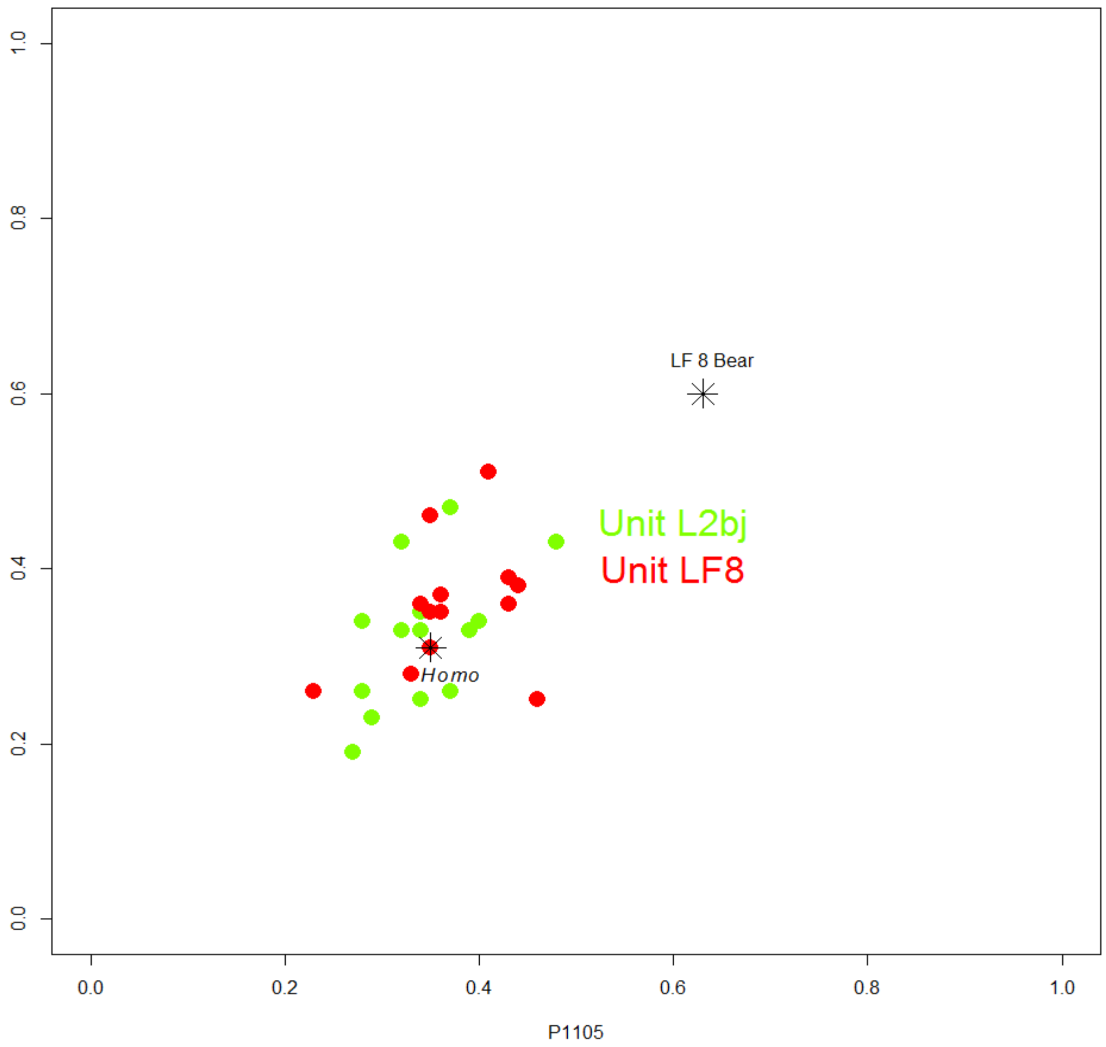

Fig. S15. Deamidation values for samples originating from level L2bj (in green) and the samples associated to LF8, including the *Bos/Bison* remains, the hominin identified through ZooMS (both in red), and the bear fragment found in stratigraphic association with LF8.

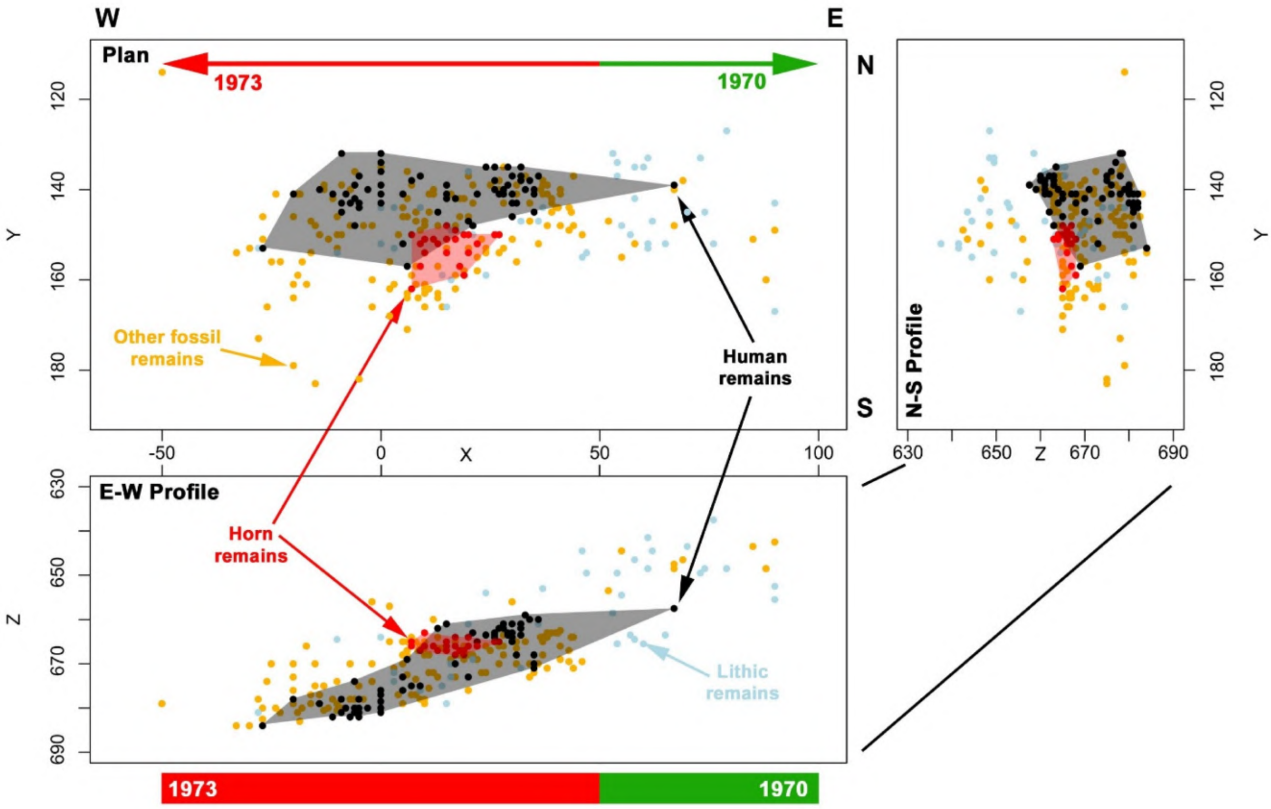

Fig. S16. 3D Distribution of the findings from square 1 in layer M2: identified human remains (black); other fossil remains (orange): these correspond to either unidentifiable specimens or where there is a lack of correspondence between the label and object; lithics (light blue); and fragments of the horn-core (red). X axis corresponds to west-east, Y to south-north and Z to the vertical extension. Note the in the XZ profile the lower density of the finds in the 1970 field season likely due to the faster excavation (see text).

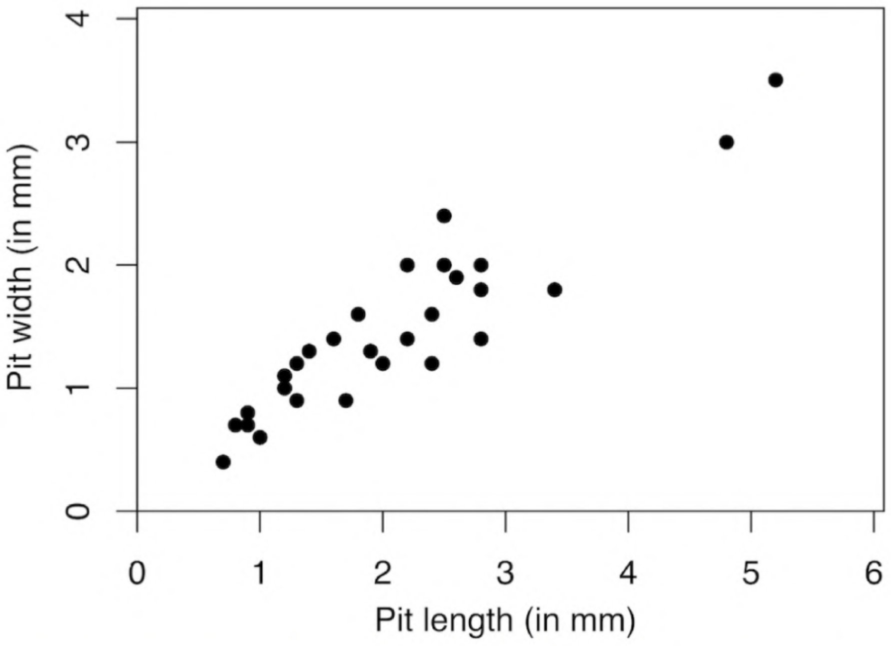

Fig. S17. Maximum length and breadth (mm) for pit marks on cortical bone (LF8 fauna, $n = 29$).

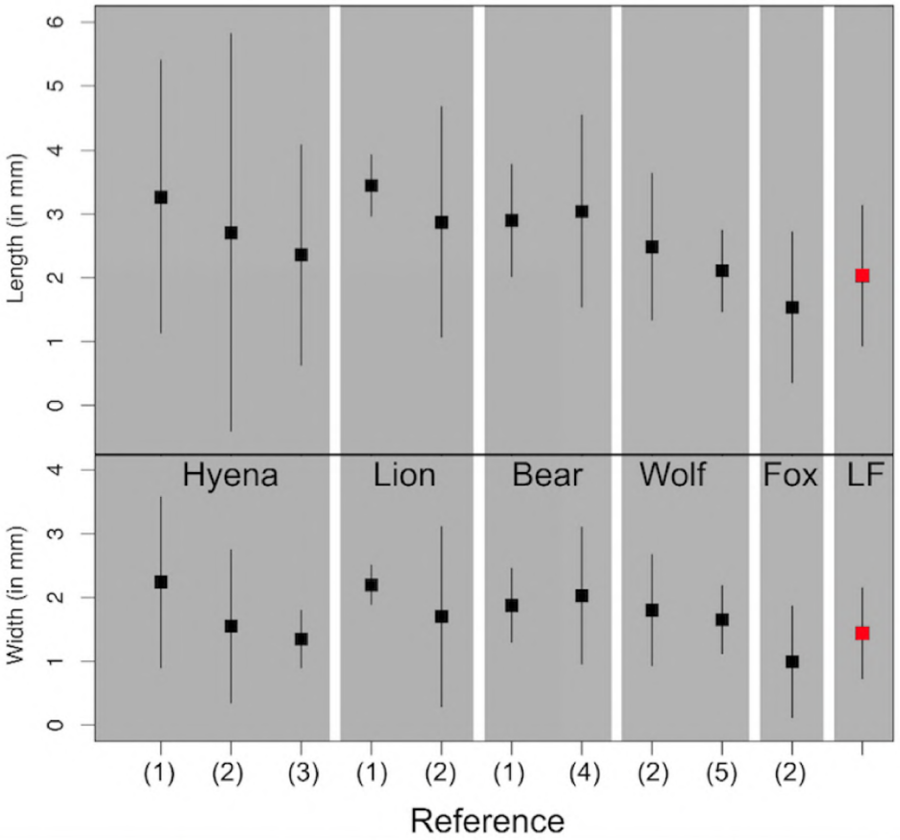

Fig. S18. Mean ± 1 standard deviation of pit length and breadth on cortical bone. References for the figure: (1) Domínguez-Rodrigo and Piqueras (72); (2) from Andres et al. (75); (3) Selvaggio and Wilder (76); (4) Saladié et al. (77) Sala et al. (78). In red, the dimensions of the pits found in the faunal remains found together with LF8 in level M2 (n = 29).

Table S1. AMS radiocarbon dating of 17 samples from La Ferrassie, amount of collagen extracted (%Coll) refer to the >30 kDa fraction, C:N ratios.

MPI-Code	Field season	Original label	Layer	% Coll	C:N	AMS Nr	^{14}C Age (BP)	1s Err	Cal BP 68.3%		Cal BP 95.4%	
									From	To	From	To
R-EVA-1386*	2014	LAF8-3	L2B-L2Bj	2.9	3.2	MAMS-25533	44,380	980	47,830	45,850	49,090	44,950
R-EVA-1374	2014	LAF8-118	L2B-L2Bj	4.1	3.2	MAMS-25531	43,010	830	46,130	44,740	47,390	44,390
R-EVA-1387	2014	LAF8-83	L2B-L2Bj	3.2	3.2	MAMS-25534	42,870	800	46,000	44,720	47,150	44,320
R-EVA-1377	2014	LAF8-143	L2B-L2Bj	2.5	3.3	MAMS-25532	39,880	570	43,830	42,720	44,230	42,550
R-EVA-1614	1970	F'1970 SQ1, layer L2Bj	L2Bj	3.4	3.2	MAMS-27343	41,070	320	44,490	43,480	44,580	43,280
R-EVA-1607	1970	F'70 SQ1	AC	6.6	3.2	MAMS-27340	40,720	310	44,120	43,360	44,380	43,130
R-EVA-1608	1970	F'70 SQ1, layer of the child	AC	6.5	3.2	MAMS-29545	41,820	260	44,800	44,420	45,060	44,240
R-EVA-1609	1973	F'73 SQ1, layer M2 bone nº375	AC	3.5	3.3	MAMS-29546	37,670	170	42,260	42,070	42,330	41,970
R-EVA-1610	1973	F'73 SQ1, close to skull nº428	AC	6.6	3.3	MAMS-27341	41,260	350	44,590	43,980	44,740	43,340
R-EVA-1611	1973	F'73 SQ1, close to skull nº446	AC	9.6	3.3	MAMS-27342	34,770	170	40,130	39,630	40,430	39,500
R-EVA-1612	1973	F'73 SQ1, nº close to pelvis	AC	1.6	3.3	MAMS-29547	39,230	190	42,830	42,610	42,930	42,510
R-EVA-1613	1973	F'73 SQ1, nº487	AC	7.5	3.2	MAMS-29548	42,350	290	45,190	44,670	45,500	44,510
R-EVA 3336**	1970	LF-86	AC	5.7	3.3	ETH-99102.1.1	36,171	222	41,400	41,020	41,710	40,820
R-EVA 3337	1970	LF-88	AC	8.8	3.2	ETH-99103.1.1	35,396	203	40,870	40,360	41,020	40,020
R-EVA 3338	1970	LF-89	AC	7.5	3.3	ETH-99104.1.1	37,749	262	42,300	42,070	42,400	41,940
R-EVA 3339	1970	LF-92	AC	7.7	3.3	ETH-99105.1.1	40,576	361	44,040	43,240	44,340	43,020
R-EVA 3340***	1970	LF-98	AC	3.6	3.4	ETH-99106.1.1	34,032	177	39,460	39,070	39,650	38,590

Elements found in 2014 are attributed to the L2B-L2Bj complex because it was not possible to directly relate the excavated area, at the same elevation but located to the west, relative to the layers excavated in 1970.
Ages have been calibrated using OxCal 4.4 (79) using the international calibration curve IntCal20 (80).
AC=associated to the child (LF8), those elements come from the bag filed in 1970 with the objects founds in the layer M2 in the eastern part of square 1 (in green on *SI Appendix,* Fig. S10) and from the box filled in 1973 during the excavation of the hominin remains in the western part of square 1 (in red on fig. S10).
* May extend out of range, but this bone is located to the west of the area where LF8 was found. We cannot ascertain its provenience from the L2Bj layer that was located above the archaeo-paleontological layer that contained the child.
** Identified by ZooMS as a human remain, and as a Neandertal using mitochondrial DNA.
*** This bone was collected in 1970 while the area was excavated more rapidly than in 1973. Its deamidation value is different from the other elements analysed by ZooMS (*SI Appendix,* Fig. S15, indicating a different post-mortem history.

Table S2. OSL results, dose rate data.

Sample	Layer	Post-IR IRSL at 160 °C			Post-IR IRSL at 290 °C		
		D_e (Gy)	Age (ka)	KF to CDM-based, SG-OSL age ratio	D_e (Gy)	Age (ka)	KF to CDM-based, SG-OSL age ratio
LF8-1	1	596 ± 151	251 ± 64	3.6 ± 1.0	740 ± 134	312 ± 58	4.5 ± 0.9
LF8-2	3	233 ± 8	134 ± 7	1.9 ± 0.2	438 ± 27	253 ± 18	3.5 ± 0.4
LF8-3	4	429 ± 87	266 ± 54	2.4 ± 0.5	633 ± 113	393 ± 72	3.5 ± 0.7
LF8-4	5	515 ± 48	314 ± 32	2.4 ± 0.3	854 ± 52	522 ± 37	3.9 ± 0.4

Table S3. Overview of lysates, DNA extracts and libraries prepared in this study.

Experiment	Library ID	Lysate ID	Extract ID	Description	Number of library molecules	Number of control molecules
1	A26847	Lys8130	E16927	Sample 1, first extract	6.42E+10	1.05E+06
1	A26848	Lys8131	E16928	Extraction blank	1.36E+08	1.03E+06
1	A26869	-		Library blank	1.56E+08	1.13E+06
2	A31968	Lys8130	E19153	Sample 1, second extract	6.68E+10	9.16E+05
2	A31969	Lys8951	E19154	Sample 2	3.95E+10	6.83E+05
2	A31970	Lys8952	E19155	Extraction blank	1.58E+08	6.31E+05
2	A31972	-		Library blank	1.79E+08	9.40E+05

Table S4. Sequencing summary statistics obtained after enrichment for hominin mtDNA.

Exp.	Library ID	Description	Sequences generated	Mapped sequences (L ≥ 35bp, MQ ≥ 25)	Unique sequences	5' C-to-T substitution frequency [%] (95% CI)	3' C-to-T substitution frequency [%] (95% CI)	Putatively deaminated fragments
1	A26847	Sample 1, first extract	40,707	1,501	1,002	19.5 (14.2-25.7)	14.6 (10.2-20.0)	67
1	A26848	Extraction blank	2,335	304	45	0.0 (0.0-33.6)	0.0 (0.0-23.2)	0
1	A26869	Library blank	2,220	232	25	0.0 (0.0-36.9)	0.0 (0.0-60.2)	0
2	A31968	Sample 1, second extract	2,717,894	60,411	2,580	21.9 (18.5-25.6)	12.7 (10.2-15.5)	191
2	A31969	Sample 2	4,175,760	95,179	2,238	30.2 (26.3-34.4)	22.2 (18.9-25.9)	274
2	A31970	Extraction blank	304,914	76,696	433	1.3 (0.0-7.2)	1.5 (0.2-5.4)	3
2	A31972	Library blank	187,416	4,333	34	7.7 (0.2-36.0)	0.0 (0.0-41.0)	1

L = length, bp = base pairs, MQ = mapping quality, CI = binomial confidence interval

Table S5. Classification of sequenced DNA fragments according to their sharing of the Neandertal or present-day human state at diagnostic positions in the mitochondrial genome. Numbers are shown for (i) all DNA fragments and (ii) the sub-set of putatively deaminated fragments, i.e. fragments showing a C-to-T substitution at the first or last position.

			All fragments			Putatively deaminated fragments		
Exp.	Library ID	Description	Neandertal state	Present-day human state	%Nea. state	Neandertal state	Present-day human state	%Nea. state
1	A26847	Sample 1, first extract	34	200	14.5	8	0	100.0
1	A26848	Extraction blank	0	14	0.0	0	0	N/A
1	A26869	Library blank	0	5	0.0	0	0	N/A
2	A31968	Sample 1, second extract	107	510	17.3	19	0	100.0
2	A31969	Sample 2	153	359	29.9	38	1	97.4
2	A31970	Extraction blank	0	103	0.0	0	1	0.0
2	A31972	Library blank	0	8	0.0	0	1	0.0

N/A = not available

Table S6. Single grain OSL data.

Sample	Layer	Numbers of grains		CDM		MDM		IEU	
		S_T < 20% & D_0>100 Gy	Sat. (%)	D_e (Gy)	OD (%)	D_e (Gy)	Age (ka)	D_e (Gy)	Age (ka)
LF8-1	1	148 (358)	16 (25)	94 ± 4	41 ± 3	68 ± 9	43 ± 6	86 ± 3	54 ± 3
LF8-2	3	156 (352)	6 (7)	57 ± 2	42 ± 3	51 ± 2	55 ± 3	53 ± 2	56 ± 3
LF8-3	4	212 (365)	15 (20)	72 ± 3	56 ± 3	55 ± 3	67 ± 4	58 ± 2	71 ± 3
LF8-4	5	165 (321)	27 (31)	84 ± 5	63 ± 5	50 ± 6	60 ± 7	56 ± 2	66 ± 4

CDM = Central Dose Model; MDM = Minimum Dose Mode; IEU = Internal External Uncertainty model.
ST < 20% & D0>100 Gy' corresponds to the number of measured grains for which the uncertainty on the Ln/Tn ratio is less than 20 % and the D0 value (as defined in 15) is greater than 100 Gy. 'Sat. (%)' corresponds to the fraction of these grains in saturation (as defined in the main text). For these two columns, the numbers in brackets correspond to the same values, without considering the D0 selection criterion. 'D_e (Gy)' is the equivalent dose determined by the corresponding model; 'OD (%)' is the overdispersion calculated with the CDM.

Table S7. Multi-grain K-feldspar post-IR IRSL data. 'KF to CAM-based, SG-OSL age ratio' corresponds to the ratio of the multi-grain, post-IR IRSL age to the CDM-based single grain OSL age.

Sample	Layer (2014)	K (%)	U top (ppm)	U bottom (ppm)	Th (ppm)	Beta dose rate (Gy/ka)	Gamma dose rate (Gy/ka)	Cosmic dose rate (Gy/ka)	Total dose rate to quartz (Gy/ka)	Total dose rate to feldspars (Gy/ka)
LF8-1	1	0.87 ± 0.02	2.23 ± 0.10	1.78 ± 0.02	14.9 ± 0.1	1.01 ± 0.03	0.66 ± 0.07	0.35 ± 0.02	1.58 ± 0.05	2.37 ± 0.06
LF8-2	3	0.46 ± 0.01	1.56 ± 0.08	0.82 ± 0.02	4.04 ± 0.06	0.47 ± 0.02	0.66 ± 0.07	0.25 ± 0.02	0.94 ± 0.04	1.74 ± 0.05
LF8-3	4	0.44 ± 0.01	0.72 ± 0.06	0.55 ± 0.01	4.26 ± 0.05	0.41 ± 0.02	0.49 ± 0.06	0.19 ± 0.01	0.82 ± 0.04	1.61 ± 0.05
LF8-4	5	0.47 ± 0.01	0.65 ± 0.05	0.53 ± 0.01	4.53 ± 0.05	0.43 ± 0.02	0.20 ± 0.03	0.19 ± 0.01	0.84 ± 0.04	1.64 ± 0.05

Table S8. Relative abundance of faunal and human remains in the Mousterian layers M1 and M2 excavated in 1970 and 1973 (NR, NISP, MNI).

**Taxon**	**recorded NR**	**unrecorded NR**	**total NR**	**%NISP**	**MNI**
Homo neanderthalensis	**106**	**85**	**191**	**89.7%**	**1**
Cervus elaphus	2	1	3	1.4%	1
Rangifer tarandus		1	1	0.5%	1
Cervidae indet.	2		2	0.9%	
Equus ferus	3	4	7	3.3%	2 (1 fetus)
Bison sp.	1		1	0.5%	
Bos/*Bison*	8		8	3.8%	1
Faunal NISP/MNI	**16**	**6**	**22**	**10.3%**	**5**
Total NISP/MNI	**122**	**91**	**213**	**100.0%**	**6**
Middle-size ungulates	37	33	70		
Large-size ungulates	3	2	5		
Indeterminate mammals	135	4,186	4,321		
Total indeterminate	**175**	**4,221**	**4,396**		
Total	**297**	**4,312**	**4,609**		

NR = number of remains; recorded NR refers to those paleontological specimens for which we the spatial information has been recorded; NISP = number of identified specimens; MNI = minimum number of individuals.
Note that the ZooMS identified among 17 indeterminate fragments 12 *Bos*/*Bison*, one *Ursus* and one hominin.

Table S9. Number of remains (NR), number of identified specimens (NISP) and various preservation indexes for both studied series.

NR/NISP/Indexes	Human (LF8)	Fauna
Total NR	193	4,416
NISP isolated teeth	19	4
NISP	193	22
NISPa	193	127
MNI	1	5
NR recorded	108	191
NR unrecorded	85	4,225
Identification index	100.0%	2.9%
Bone destruction index	9.8%	3.1%
Completeness index	19.0%	0.0%
Coprolites	0	1

Table S10. Anatomical element identification of the LF8 human and faunal remains (NISP).

**NISP**	***Homo***	***Bos/Bison***	***Equus***	***Cervus/Rangifer***
Horn	-	1	-	
Skull	53	2		
Mandible	4	5		
Isolated teeth (lower)	11			1
Isolated teeth (upper)	8			
Isolated teeth (indet.)			1	1
Cervical vertebrae	11			
Thoracic vertebrae	18			
Lumbar vertebrae	9			
Sacrum	5			
Coxal	5			
Rib	63			
Scapula			1	
Humerus				1
Radio-ulna		1	1	
Carpal				
Metacarpal			1	2
Hand phalanx 1	4			
Hand phalanx 2				
Hand phalanx 3				
Femur				1
Tibia			1	
Tarsal				
Metatarsal			1	
Foot phalanx 1				
Foot phalanx 2				
Foot phalanx 3				
Indeterminate metapodials			1	
Sesamoid				
Total NISP	172	9	6	4
Total NISP (with teeth)	191	9	7	6

Table S11. Number of remains (NR)[a] by size classes (in mm) of the human and faunal remains.

Taxon		Size classes (in mm)							Total
		**0-25**	**26-50**	**51-75**	**76-100**	**101-125**	**126-150**	**151-175**	
**Human**	NR	121	16	3	1				141
	**%**	85.8%	11.3%	2.1%	0.7%	0.0%	0.0%	0.0%	100.0%
**Fauna**	NR	757	85	12	5			1	860
	**%**	88.0%	9.9%	1.4%	0.6%	0.0%	0.0%	0.1%	100.0%

[a] NR without teeth, burnt bones and complete elements.

Table S12. Climatic, edaphic and biotic alterations for the human and faunal remains.

Alteration	Human						Fauna						z score	%χ^2
	Stages			n Total	NR total	% (C.I.)	Stages			n Total	NR total	% (C.I.)		
	1	2	3				1	2	3					
**Abiotic alterations χ^2=50.89 (df = 7; *p* < 0.001)**														
Cracking	103	2		105	174	60.3 (1.2)	66	5		71	177	40.1 (0.9)	**3.79[1]**	**14.1**
Desquamation	86	18		104	174	59.8 (1.1)	25	18	12	55	177	31.1 (0.8)	**5.40[1]**	**31.3**
Smooth edges	19			19	174	10.9 (0.5)	5			5	177	2.8 (0.2)	**3.00[2]**	**16.5**
Concretion	17			17	174	9.8 (0.5)	7	2		9	177	5.1 (0.3)	1.68	5.1
Chemical corrosion	99	3		102	174	58.6 (1.1)	64	13	1	78	177	44.1 (1.0)	**2.73[2]**	**7.1**
Root marking				0	174	0	3			3	177	1.7 (0.2)	-1.72	5.8
Oxides (black coloration)	143	5		148	174	85.1 (1.4)	98	50	1	149	177	84.2 (1.3)	0.23	0
Illegible remains				9	174	5.2 (0.3)				29	177	16.4 (0.6)	**-3.38[1]**	**20.0**
**Patterns of fragmentation χ^2 = 672.09 (df = 3; *p* < 0.001)**														
Bone completeness				33	174	19.0 (0.6)				0	879	0	**13.12[1]**	
Green bone fracture				0	141	0				118	860	13.7 (0.2)	**-4.68[1]**	**2.9**
Dry bone fracture				0	141	0				45	860	5.2 (0.2)	**-2.78[2]**	**1.1**
Recent fracture				99	141	70.2 (1.4)				50	860	5.8 (0.2)	**19.91[1]**	**50.2**
Indeterminate fracture				138	141	97.9 (1.6)				131	860	15.2 (0.3)	**20.52[1]**	**45.8**
**Biotic and indeterminate surface alterations χ^2 =223.14 (df=4; *p* < 0.001)**														
Burnt				0	174	0				12	121	9.9 (0.6)	**-4.24[1]**	**7.7**
Cut				0	174	0				39	121	32.2 (1.0)	**-8.04[1]**	**25.1**
Carnivore marks				0	174	0				18	121	14.9 (0.7)	**-5.25[1]**	**11.6**
Indeterminate surface alterations				103	174	0.59195402				5	121	4.1 (0.4)	**9.66[1]**	**26.5**
Recent surface alterations				118	174	0.67816092				7	121	5.8 (0.4)	**10.60[1]**	**29.0**

NR = recorded bone remains and unrecorded bone fragments > 5 cm long.
χ^2 values: the two samples are significantly different regarding abiotic, biotic alterations and fragmentation patterns; %χ^2: χ^2 contribution of each alteration; C.I.: Confidence Intervals; Z-score: significant values in bold ([1] = p < 0.001; [2] = p < 0.01; [3] = p < 0.05).

Table S13. Distribution of the animal long bone shafts according sizes and circumferences[a].

Fauna LF8	Raw data					Percentage				
	L1	L2	L3	L4	TOTAL C	L1	L2	L3	L4	TOTAL C
**C1**	61	3			64	92.4%	4.5%			97.0%
**C2**		2			2	0.0%	3.0%			3.0%
**C3**					0					0.0%
**TOTAL L**	61	5			66	92.4%	7.6%			100.0%

[a]L1 ≤ 1/4; L2 ≤ 1/2; L3 ≤ 3/4 and L4 ≤ 1; C1 < 1/2; C2 ≥ 1/2 and C3 = 1.

Table S14. Number and rates of burnt faunal elements by size classes (mm), tissue type (*spongiosa*, *compacta* or tooth) and degree of burning (non-carbonized: brown or red coloration; carbonized: black coloration; calcined: grey or white coloration).

Burnt elements	Non-carbonized	Carbonized	Calcined	Total	%	Total NR
***Spongiosa* 0-25mm**	39	2,663	138	2,840	82.3%	3,452
***Compacta* 0-25mm**	32	551	47	630	79.9%	788
***Spongiosa* 26-50mm**	3	8	1	12	13.2%	91
***Compacta* 26-50mm**	0			0	0.0%	24
***Comp*/*Spong* > 50**				0	0.0%	20
**Isolated teeth**	5	5		10	24.4%	41
**Total**	79	3,227	186	3,492	79.1%	4,416

References

1. J.L. Heim, Les enfants néandertaliens de la Ferrassie. Étude anthropologique et analyse ontogénique des hommes de Néandertal. (Masson, Paris, 1982b).

2. B. Maureille, A lost Neanderthal neonate found. *Nature* **419**, 33-34 (2002).

3. D. Laville, *La Ferrassie : taphonomie d'un site sépulcral moustérien* Master thesis, Université de Liège (2007).

4. J.L. Heim, Les Hommes fossiles de la Ferrassie. I. Le gisement. Les squelettes adultes (crâne et squelette du tronc). (Masson, Paris, 1976).

5. J.L. Heim, Les hommes fossiles de La Ferrassie. II. Les squelettes d'adultes: squelettes des membres. (Masson, Paris, 1982a).

6. G. Becam, C. Verna, A. Gómez-Robles, A. Gómez-Olivencia, et al., Isolated teeth from La Ferrassie: Reassessment of the old collections, new remains, and their implications. Am J Phys Anthropol **169**(1), 132-142 (2019).

7. A. Gómez-Olivencia, I. Crevecoeur, A. Balzeau. La Ferrassie 8 Neandertal child reloaded: New remains and re-assessment of the original collection. *J Hum Evol* **82**, 107-126 (2015).

8. H. Delporte, *Le grand abri de la Ferrassie* (Éditions du Laboratoire de Paléontologie Humaine et de Préhistoire, 1984).

9. B. Maureille, D. Laville, P. Mennecier, in *Première Humanité. Gestes funéraires des Néandertaliens* (eds B. Vandermeersch *et al.*) 110 (Musée National de Préhistoire, Les Eyzies de Tayac, 2008).

10. A. Turq, et al., Reprise des fouilles dans la partie ouest du gisement de la Ferrassie, Savignac-de-Miremont, Dordogne : problématique et premiers résultats In: Bertran, P., Lenoble, A. (eds.), Quaternaire continental d'Aquitaine : un point sur les travaux récents. Excursion AFEQ-ASF 30 mai-01 juin 2012 (ASF-AFEQ, Bordeaux, 2012) pp. 78–87.

11. G. Guérin, M. Frouin, S. Talamo, V. Aldeias, L. Bruxelles, et al., A multi-method luminescence dating of the Paleolithic sequence of La Ferrassie based on new excavations adjacent to the La Ferrassie 1 and 2 skeletons. *J Archaeol Sci* **58**, 147-166 (2015).

12. C. Thiel, J.P. Buylaert, A. Murray, B. Terhorst, I. Hofer, S. Tsukamoto, M. Frechen, Luminescence dating of the Stratzing loess profile (Austria) - testing the potential of an elevated temperature post-IR IRSL protocol. *Quaternary International* **234**, 23-31 (2011).

13. G.A.T. Duller, L. Bøtter-Jensen, A.S.Murray, A.J. Truscott, Single grain laser luminescence (SGLL) measurements using a novel automated reader. *Nuclear Instruments and Methods* B **155**, 506–514 (1999).

14. A.S. Murray, A.G. Wintle, Luminescence dating of quartz using an improved single-aliquot regenerative-dose protocol. *Radiation Measurements* **32**, 57-73 (2000).

15. K.J. Thomsen, A.S. Murray, J.-P. Buylaert, M. Jain, J. Helt-Hansen, T. Aubry, Testing single-grain quartz OSL methods using known age samples from the Bordes-Fitte rockshelter (Roches d'Abilly site, Central France). *Quaternary Geochronology* **31**, 77–96 (2016).

16. G. Guérin, N. Mercier, G. Adamiec, Dose-rate conversion factors: update. *Ancient TL* **29**, 5-8 (2011).

17. G. Guérin, N. Mercier, R. Nathan, G. Adamiec, Y. Lefrais, On the use of the infinite matrix assumption and associated concepts: a critical review. *Radiation Measurements* **47**, 778-785 (2012).

18. G. Guérin, N. Mercier, Preliminary insight into dose deposition processes in sedimentary media on a grain scale: Monte Carlo modelling of the effect of water on gamma dose-rates. *Radiation Measurements* **47**, 541-547 (2012).

19. R. Longin, New method of collagen extraction for radiocarbon dating. *Nature* **230**(5291), 241-242 (1971).

20. T. A. Brown, *et al.*, Improved Collagen Extraction by modified Longin method. *Radiocarbon* **30**(2), 171-177 (1988).

21. F. Brock, *et al.*, Quality assurance of ultrafiltered bone dating. *Radiocarbon* **49**(2), 187–192 (2007).

22. P. Korlević, *et al.*, A combined method for DNA analysis and radiocarbon dating from a single sample. *Scientific Reports* **8**(1), 4127 (2018).

23. B. Kromer, *et al.,* MAMS – A new AMS facility at the Curt-Engelhorn-Centre for Achaeometry, Mannheim, Germany. *Nuclear Instruments and Methods in Physics Research Section B: Beam Interactions with Materials and Atoms* **294**(0), 11-13 (2013).

24. H. Fewlass, T. Tuna, Y. Fagault, J.-J. Hublin, B. Kromer, E. Bard, S. Talamo, Pretreatment and gaseous radiocarbon dating of 40–100 mg archaeological bone. *Scientific Reports*, **9**(1), 5342 (2019).

25. L. Wacker, M. Němec, J. Bourquin, A revolutionary graphitisation system: Fully automated, compact and simple. *Nuclear Instruments and Methods in Physics Research B* **268**(7-8), 931-4 (2010).

26. L. Wacker, G. Bonani, M. Friedrich, I. Hajdas, B. Kromer, et al., MICADAS: routine and high-precision radiocarbon dating. *Radiocarbon* **52**(2-3), 252-262 (2010).

27. F. Welker, M. Hajdinjak, S. Talamo, K. Jaouen, M. Dannemann, et al., Palaeoproteomic evidence identifies archaic hominins associated with the Châtelperronian at the Grotte du Renne. *Proc Natl Acad Sci USA* **113**(40), 11162-11167 (2016).

28. M. Buckley, M. Collons, J. Thomas-Oates, J.C. Wilson, Species identification by analysis of bone collagen using matrix−assisted laser desorption/ionisation time−of−flight mass spectrometry. *Rapid Commun. Mass Spectrom*. 23, 3843-3854 (2009).

29. N.L. Van Doorn, J. Wilson, M. Soressi, M.J., Collins Site-specific deamidation of glutamine: a new marker of bone collagen deterioration. *Rapid Commun. Mass Spectrom*. **26**, 2319–2327 (2012).

30. Rohland, N., et al., Extraction of highly degraded DNA from ancient bones, teeth and sediments for high-throughput sequencing. *Nat Protoc* **13**, 2447-2461 (2018).

31. Gansauge, M. T., et al., Manual and automated preparation of single-stranded DNA libraries for the sequencing of DNA from ancient biological remains and other sources of highly degraded DNA. *Nat Protoc*, in press.

32. Maricic, T., et al., Multiplexed DNA sequence capture of mitochondrial genomes using PCR. *PLoS One* **5**, e14004 (2010).

33. Slon, V., et al., Neandertal and Denisovan DNA from Pleistocene sediments. *Science* **356**, 605-608 (2017).

34. Andrews, R.M., et al., Reanalysis and revision of the Cambridge reference sequence for human mitochondrial DNA. *Nat Genet* **23**, 147 (1999).

35. Fu, Q., et al. DNA analysis of an early modern human from Tianyuan Cave, China. *Proc Natl Acad Sci USA* **110**, 2223-2227 (2013).

36. Renaud, G., et al., leeHom: adaptor trimming and merging for Illumina sequencing reads. *NAR* **42**, e141 (2014).

37. Li, H., Durbin, R., Fast and accurate long-read alignment with Burrows-Wheeler transform. *Bioinformatics* **26**, 589–595 (2010).

38. Meyer, M., et al., A High-Coverage Genome Sequence from an Archaic Denisovan Individual. *Science* **338,** 222-226 (2012).

39. Briggs, et al., Patterns of damage in genomic DNA sequences from a Neandertal. *Proc Natl Acad Sci USA* **104**, 14616-14621 (2007).

40. Green, R. E., et al., A complete Neandertal mitochondrial genome sequence determined by high-throughput sequencing. *Cell* **134**, 416–426 (2008).

41. L.R. Binford, Faunal Remains from Klasies River Mouth (Academic Press, New York, 1984).

42. R.L. Lyman, Vertebrate Taphonomy (Cambridge University Press, 1994) 524 p.

43. P. Villa, E. Mahieu. Breakage patterns of human long bones. *J. Hum. Evol.* **21**, 27-48 (1991).

44. R.J. Blumenschine, M.M. Selvaggio, Percussion marks on bone surfaces as a new diagnostic of hominid behavior. *Nature* **333**, 763-765 (1988).

45. R.J. Blumenschine, M.M. Selvaggio, On the marks of marrow bone processing by hammerstones and hyenas: their anatomical patterning and archaeological implications. In: Clark, J.D. (Ed.), Cultural Beginnings – Approaches to Understanding Early Hominid Life-ways in the African Savanna, (UISPP, Mainz, 1987) pp. 17–32.

46. A. Grant. The use of tooth wear as a guide to the age of domestic ungulates. In (Wilson, B., Grigson, C. & Payne, S., eds.), Ageing and sexing animal bones from archaeological sites, (British Archaeological Reports International Series, 1982), S109, p. 91-108.

47. R.G. Klein, C. Cruz-Uribe, The Analysis of Animal Bones from Archaeological Sites. (University of Chicago Press, 1984).

48. A.K. Behrensmeyer. Taphonomic and ecologic information from bone weathering. *Palaeobiology* **4**, 150–162 (1978).

49. L.R. Binford Bones: Ancient Men and Modern Myths (Academic Press, New York, 1981).

50. R.L. Lyman. Vertebrate taphonomy (Cambridge University Press., Cambridge, 1994).

51. J.W. Fisher, Bone surface modifications in zooarchaeology. *Journal of Archaeological Method and Theory* **2(1)**, 7-68 (1995).

52. P. Shipman, J. J. Rose, Early hominid hunting, butchering, and carcass processing behaviors: approaches to the fossil record. *Journal of Anthropological Archaeology* **2**, 57-98 (1983).

53. P. Shipman, J. J. Rose, Cutmark Mimics on Modern and Fossil Bovid Bones. *Current Anthropology* **25**, 1, 116-117 (1984).

54. A.K. Behrensmeyer, K.D. Gordon, G.T. Yanagi, Trampling as a cause of bone surface damage and pseudo-cutmarks. *Nature* **319**, 768-771 (1986).

55. S. L. Olsen, P. ShipmanSurface modification on bone: trampling versus butchery. *J. Archaeol. Sci.* **15**, 535-553 (1988).

56. R. Blasco, J. Rosell, J.F. Peris, I. Caceres, J.M. Vergès, A new element of trampling: an experimental application on the Level XII faunal record of Bolomor Cave (Valencia, Spain). *J. Archaeol. Sci.* **35**, 1605-1618 (2008).

57. M. Domínguez-Rodrigo, S. de Juana, A.B. Galan, M. Rodriguez, A new protocol to differentiate trampling marks from butchery cut marks. *J. Archaeol. Sci.* **36**, 2643-2654 (2009).

58. P.J. Nilssen, An actualistic butchery study in South Africa and its implications for reconstructing hominid strategies of carcass acquisition and butchery in the Upper Pleistocene and Plio-Pleistocene. Ph.D. Dissertation, University of Cape Town (2000).

59. B.L. Pobiner, M.J. Rogers, C. M. Monahan, J.W.K. Harris, New evidence for hominin carcass processing strategies at 1.5 Ma, Koobi Fora, Kenya. *J. Hum. Evol.* **55**, 103-130 (2008).

60. G. Haynes, A guide for differentiating mammalian carnivore taxa responsible for gnaw damage to herbivore limb bones. *Paleobiology* **9** (2), 164-172 (1983).

61. E. Campmas, C. Beauval, Consommation osseuse des carnivores: résultats de l'étude de l'exploitation de carcasses de boeufs (*Bos taurus*) par des loups captifs. *L'Anthropologie* **94**, 167-186 (2008).

62. T.R. Pickering, M. Dominguez-Rodrigo, C.P. Egeland, C.K. Brain, Beyond leopards: tooth marks and the contribution of multiple carnivore taxa to the accumulation of the Swartkrans Member 3 fossil assemblage. *J. Hum. Evol.* **46**, 595-604 (2004).

63. H. Delporte, Les fouilles du Musée des Antiquités Nationales à la Ferrassie. *Antiquités Nationales* **1**, 15-28 (1969).

64. H. Laville, A. Tuffreau, in *Le grand abri de la Ferrassie* (ed H. Delporte) 25-50 (Éditions du Laboratoire de Paléontologie Humaine et de Préhistoire, 1984).

65. M.D Garralda, B. Maureille, Y. Pautrat, B. Vandermeersch, La molaire d'enfant néandertalien de Genay (Côte-d'Or). *Paleo* **20**, 89-100 (2008).

66. S. Bailey, Neandertal dental Morphology. PhD Dissertation (Arizona State University, 2002).

67. A.G. Wintle, A.S. Murray, A review of quartz optical stimulated luminescence characteristics and their relevance in single-aliquot regeneration dating protocols. *Radiation Measurements* **41**, 369-391 (2006).

68. R.F. Galbraith, R.G. Roberts, G.M. Laslett, H. Yoshida, J.M. Olley, Optical dating of single and multiple grains of quartz from Jinmium rock shelter, northern Australia: Part I, experimental design and statistical models. *Archaeometry* **41**, 339–364 (1999).

69. K.J. Thomsen, A.S. Murray, L. Bøtter-Jensen, J. Kinahan, Determination of burial dose in incompletely bleached fluvial samples using single grains of quartz. *Radiation Measurements* **42**, 370-379 (2007).

70. Delpech, F. in *Le grand abri de la Ferrassie* (ed H. Delporte) 61-89 (Éditions du Laboratoire de Paléontologie Humaine et de Préhistoire, 1984).

71. B. Martínez-Navarro, J. Antonio Pérez-Claros, M.R. Palombo, L. Rook, P. Palmqvist, The Olduvai buffalo *Pelorovis* and the origin of *Bos*. *Quaternary Research* **68**, 220-226 (2007).

72. M. Domínguez-Rodrigo, A. Piqueras, The use of tooth pits to identify carnivore taxa in tooth-marked archaeofaunas and their relevance to reconstruct hominid carcass processing behaviours. *J. Archaeol. Sci.* **30**, 1385-1391 (2003).

73. S. Costamagno, I. Théry-Parisot, J.-C. Castel, J.-P. Brugal, Combustible ou non? Analyse multifactorielle et modèles explicatifs sur des ossements brûlés paléolithiques. In Théry-Parisot I., Costamagno S. et Henry A. (Eds.): Fuel Management during the Paleolithic and Mesolithic Periods. New tools, new interpretations, BAR International Series 1914, p. 65-84 (2009).

74. D. Peyrony, La Ferrassie : Moustérien-Périgordien-Aurignacien. *Préhistoire*, 1-143 (1934).

75. M. Andres, A.O. Gidna, J. Yrevedra, M. Domínguez-Rodrigo, A study of dimensional differences of tooth marks (pits and scores) on bones modified by small and large carnivores. *Archaeological and Anthropological Sciences* **4**, 209–219 (2003).

76. M.M. Selvaggio, J.Wilder, Identifying the Involvement of Multiple Carnivore Taxa with Archaeological Bone Assemblages. *J. Archaeol. Sci.* **28**, 465-470 (2001).

77. P. Saladié, R. Huguet, A. Rodríguez-Hidalgo, I. Cáceres, M. Esteban-Nadal, J.L. Arsuaga, J.M. Bermúdez de Castro, E. Carbonell, Intergroup cannibalism in the European Early Pleistocene: The range expansion and imbalance of power hypotheses. *J. Hum. Evol.* **63**, 682-695 (2012).

78. N. Sala, et al., Carnivore activity in the Sima de los Huesos (Atapuerca, Spain) hominin simple. *Quat. Sci. rev.* **97**, 71-83 (2014).

79. C. Bronk Ramsey, S. Lee, Recent and Planned Developments of the Program OxCal. *Radiocarbon* **55**, 720-730 (2013).

80. P. Reimer et al., The IntCal20 Northern Hemisphere Radiocarbon Age Calibration Curve (0–55 cal kBP). *Radiocarbon* **62**, 725–757 (2020).
