# Supplementary material for: Pluridisciplinary evidence for burial for the La Ferrassie 8 Neandertal child
Source: Sci Rep. 2020 Dec 9;10:21230. doi: 10.1038/s41598-020-77611-z (PMC7725784; doi:10.1038/s41598-020-77611-z)
Supplement: Supplementary file 2 — Supplementary Information 2. [file 41598_2020_77611_MOESM2_ESM.pdf]

74

Ferrassie	Le : / / 70	Page N° 1
Nom et adresse :		CARRÉ : 1
		Visées faites sur : △ = 516,5
		= m.

Observations : (Y a-t-il un relevé, commentaire, photo, ?)

M ou A	N°	X	Y	Z	△ ± Z	Orientation	Pendage	Dimensions	Croquis forme	Désignation et nature	Niveau ou sol
+	1	53	97	92	608,5	NS	↘	45	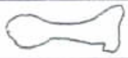	grattoir atypique	L2b
+	2	55	100	93	609,5	EO		55		fragment de nucleus	?
+	3	60	98	94	610,5	EO	→	55		pièce avec quelques retouches	
–	4	47	110	92	608,5	EO	→	35	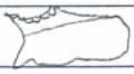	pièce à encoche	
+	5	60	112	97	613,5	EO	↘	35		fragment de racloir	
+	6	63	118	95	611,5	NS		30		pièce retouchée	
+	7	46	132	86	602,5	NS	.	40	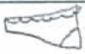	fragm. de racloir	
+	8	53	136	90	606,5	NS	→	50		pièce lustrée	
+	9	84	138	92	608,5	EO		35	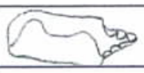	carène atypique?	
+	10	93	140	93	609,5	EO		30		Pièce retouchée	
+	11	104	143	93	609,5	EO		33	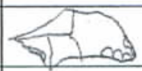	carène atypique?	
+	12	43	123	87	603,5	EO	↘	35	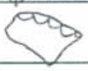	fragment racloir	
+	13	42	136	84	600,5	NS		32	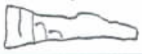	pièce à enlèvements lamellaires	
+	14	43	162	89	605,5	EO	↘	28		pièce retouchée ?!?	
+	15	50	143	90	606,5	EO		45	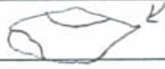	burin dièdre	
+	16	55	46	91	607,5	?		26		pièce retouchée	
+	17	67	44	93	609,5	EO		30		pièce retouchée	
+	18	71	47	93	609,5	–		35		pièce retouchée	
+	19	57	48	90	606,5	NS		17		phalangette (renne)	
+	20	61	54	93	609,5	EO		30	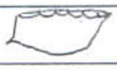	micro racloir?	
+	21	57	103	95	611,5	NS		55	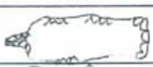	grattoir s/lame retouchée pédonculé	
+	22	45	116	89	605,5	EO	↓	40	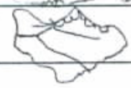	pièce retouchée lustrée	
+	23	50	125	91	607,5			60		pièce retouchée	
+	24	44	127	92	608,5			30		pièce retouchée lustrée	

Ferrassie

Le : 14 / 8 / 70

Page N° 2

CARRÉ : 1

Nom et adresse :

Visées faites sur : Δ = 516,5

= m.

Observations : (Y a-t-il un relevé, commentaire, photo, ?)

M ou A	N°	X	Y	Z	Δ ± Z	Orientation	Pendage	Dimensions	Croquis forme	Désignation et nature	Niveau ou sol
+	25	57	132	93	609,5	EO		30 m/m	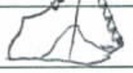	Pièce retouchée lustrée	L2b
+	26	43	132	91	607,5	NS				Fragment molaire brisé	
+	27	52	139	91	607,5	NE SE		45 m/m	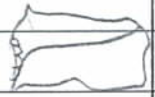	Nucleus Rabot ?	
+	28	58	150	92	608,5	NS		40 m/m		Denticulée retouchée	
+	29	46	85	94	610,5			45 m		Nucleus	
+	30	52	89	98	614,5	EO	←	59	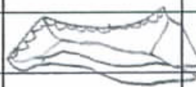	Lame retouchée	
+	31	58	102	99	615,5	EO	↑.	35		genre burin caréné	
+	32	62	100	96,5	618	NO SE		70	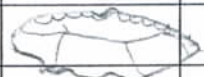	Racloir	
+	33	48	122	96	612	EO		65		Nucleus	
+	34	44	98	90	606,5	EO		45		os roulé et lustré	
+	35	62	96	100	616,5	NE SO	↘	40	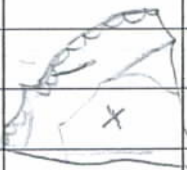	grattoir atypique	
+	36	59	103	98,5	615	EO		50		Racloir Perçoir	
+	37	49	104	97,5	614	NS	↓	34		Racloir	
+	38	44	107	96	612,5	NS		50	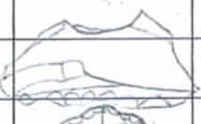	Pièce Retouchée	
+	39	57	114	96	612,5	NS	↓	45		Burin ?	
+	40	59	113	96	612,5		↓	45	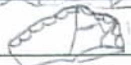	Fragment racloir	
+	41	46	116	95	611,5	EO		45	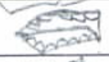	Racloir convergent	
+	42	43	120	92	608,5	NS	↘	40	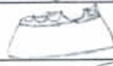	Pièce à encoche	
+	43	45	102	92,5	609	EO		75 m/m	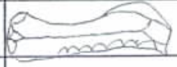	Lame retouchée	
+	44	52	104	96	612,5			28		Dent de boeuf	
+	45	58	99	102	618,5	NS	→	35 m/m		Pièce retouchée	
+	46	57	106	101	617,5	NS		45 m/m	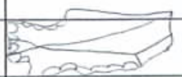	grattoir	
+	47	44	119	96	612,5			30 m/m		Pièce lustrée	
+	48	49	110	99	615,5	NS	→	30 m/m		fragment racloir	

26

Ferrassie

Le : 15/8/70

Page N° 3

CARRÉ : 1

Visées faites sur : △ = 516,5

.... = ... m.

Nom et adresse :

Observations : (Y a-t-il un relevé, commentaire, photo, ... ?)

M ou A	N°	X	Y	Z	△ ± Z	Orientation	Pendage	Dimensions	Croquis forme	Désignation et nature	Niveau ou sol
+	49	65	120	97	613,5	NS	→	34 m/m		Piece retouchée	L2b
+	50	47	128	97	613.5	NS		48 m/m		Lame retouchée	
+	51	50	125	98	614.5	NS	→	30 m/m		Fragment denticulé retouché	
+	52	51	85	105	621.5	NS	→	40 m/m	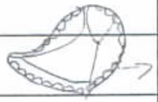	Racloir convergent	
+	53	51	90	102	618,5	NS		40 m/m		Piece retouchée	
+	54	54	93	102.5	619	NS	↘	40 m/m		Piece retouchée	
+	55	47	95	99	615,5		↓	42 m/m		Piece retouchée	
+	56	58	98	103	619.5	NS	→	35 m/m		Piece retouchée	
+	57	60	102	102.5	619	EO		45 m/m	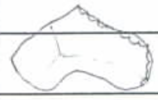	Denticulée retouchée	
+	58	42	106	100	616,5	NS		35 m/m		Piece retouchée	
+	59	40	118	98	614,5	NS	→	58 m/m	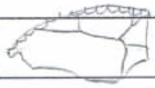	Lame dos abattu (abri audi)	
+	60	56	117	100.5	617	NS	→	45 m/m		Piece dos abattu	
+	61	43	125	97.5	614			50 m/m	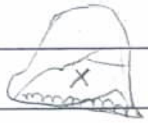	Racloir	
+	62	48	131	96	612,5	NS	→	40 m/m		Piece retouchée	
+	63	53	131	96	612,5	NS	→	35 m/m		Piece retouchée	
+	64	52	92	102	618,5		↘	34		Piece denticulée retouchée	
+	65	65	93	107	623,5	EO	→	36		Denticulé retouché	
+	66	43	108	100		EO		42		Fragment lame retouchée	
+	67	54	10[illegible]	95		EO		38		Fragment lame retouchée	
+	68	44	83	102	618,5	EO	↓	50 m/m	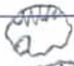	Prototype de carenée museau retouches inverse	
+	69	43	89	102	618,5	NS	↓	60 m/m	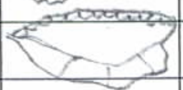	Racloir	
+	70	47	86	105,5	622	NS	←	30		Denticulée encoche	
+	71	52	96	102,5	625	NS		25		Fragment de lame retouchée abrupte	
+	72	55	101	107,5	624	NS	→	20		Denticulée retouchée	

77

Ferrassie

Le : 17 / 8 / 70

Page N° 4

CARRÉ : 1

Nom et adresse :

Visées faites sur : △ = 516,5

= m.

Observations : (Y a-t-il un relevé, commentaire, photo, ?)

M ou A	N°	X	Y	Z	△ ± Z	Orientation	Pendage	Dimensions	Croquis forme	Désignation et nature	Niveau ou sol
+	73	58	101	107,5	624			20		Denticulée encoche	L2b
+	74	57	98	110,5	624	EO		40		Denticulée retouchée	
+	75	57	109	104	620,5	EO		40		Pièce retouchée	
+	76	62	108	107	623,5	NS		30		Denticulé encoche retouche	
+	77	42	122	98	614,5	NS	↓	40		Denticulé retouché	
+	78	45	121	100	616,5	NS	→	35		"	
+	79	50	116	102	618,5	EO		50	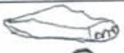	Lame retouchée	
+	80	54	116	101	617,5	EO		40	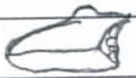	Pièce retouchée cintrée Burin ?	
+	81	54	119	102	618,5	NS	↘	45		Denticulée	
+	82	62	120	101	617,5	NS	→	35		Racloir	
+	83	44	90	102	618,5	NS	↓	55	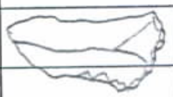	Denticulé retouché	
+	84	45	98	103	619,5	NS	↘	40		Denticulé retouché	
+	85	46	68	110	626,5	NS	↘	45		pièce retouchée	
+	86	57	75	107	623,5	EO		56	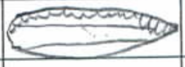	belle lame retouchée	
+	87	45	86	105	621,5	NS	↓	52		pièce retouchée	
+	88	48	37	112	628,5	EO		30		Pièce retouchée	
+	89	55	38	113	629,5			35		Fragment de nucléus	
+	90	58	35	113	629,5	NS	↓	45		Pièce retouchée	
+	91	60	44	112	628,5	NS		40		Denticulée	
+	92	51	46	112	628,5	EO	→	40		Pièce retouchée	
+	93	50	56	110	626,5			45		Couteau à dos	
+	94	55	53	113	629,5	EO	↓	40		Pièce retouchée	
+	95	62	61	114	630,5	EO		40		Petit racloir	
+	96	49	67	111	627,5	EO	↓	45		Denticulée retouchée	

Ferrassie

Le : 18 / 8 / 70

Page N° 6

CARRÉ : 1

Visées faites sur : △ = 516,5

Nom et adresse :

.... = ... m.

Observations : (Y a-t-il un relevé, commentaire, photo, ... ?)

M ou A	N°	X	Y	Z	△ ± Z	Orientation	Pendage	Dimensions	Croquis forme	Désignation et nature	Niveau ou sol
x	116	47	130	97	613,5	E O		35		Denticulée	L 265
x	117	48	137	97	613,5	E O	↓	45	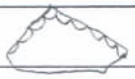	Denticulée lustrée	
x	118	58	141	99	615,5	E O	→	40		Denticulée	
x	119	73	143	101	617,5	E O	↓	28		Denticulée	
x	120	91	135	99	615,5	NS				Denticulée	
x	121	89	137	99	615,5	NS		30		Fragment de racloir	
x	122	98	138	97	613,5	NS		25		Fragment de lame retouché	
x	123	95	144	97	613,5			33	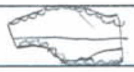	Fragment de lame avec cran à la base	
x	124	105	140	97	613,5	NS	→	45		Denticulée	
x	125	91	149	98	614,5	EO	→	30		Pièce retouchée	
x	126	114	147	100	616,5			23	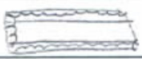	Fragment de lamelle retouché	
x	127	45	144	98	614,5	NS		40		Eclat retouché	
x	128	46	149	99	615,5			L 25 l 15	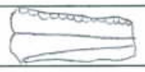	Fragment de lamelle retouché	
x	129	42	136	95	611,5	N'S	↓	35		grattoir s/lame	
x	130	44	131	100	616,5	NS	↘	50		gros éclat travaillé	
x	131	46	134	98	614,5	NS		40		Denticulée retouchée	
x	132	45	138	98	614,3			22		Pièce à encoches	
x	133	46	146	101	617,5	EO		25		Fragment de racloir	
x	134	78	136	101	617,5	E O		30		Eclat retouché	
x	135	82	133	101	617,5			30		Eclat retouché	
x	136	72	142	101	617,5			55		Eclat à fines retouches	
x	137	94	137	99	615,5	EO		35		[illegible]	
x	138	110	140	99	615,5	NS		30		Fragment de lame retouché encoche	
x	139	44	132	101	617,5	NS		40		Pièce retouchée	

86

Ferrassie

Nom et adresse :

Page N° 7

Le : 18/8/70

CARRÉ : 1

Visées faites sur : △ = 516,5

= m.

Observations : (Y a-t-il un relevé, commentaire, photo, ?)

M ou A	N°	X	Y	Z	△ ± Z	Orientation	Pendage	Dimensions	Croquis forme	Désignation et nature	Niveau ou sol
+	140	45	142	100	616,5	NS	—	33	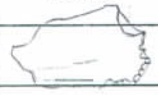	Grattoir	
+	141	40	121	98	614,5	NS	↘	30		Fragment de lame retouchée	
+	142	56	127	102	618,5	EO	—	30		Lamelle retouchée	
+	143	58	124	102	618,5	EO		38		Piece retouchée	
+	144	63	151	102	618,5	NS		40	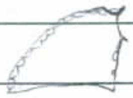	Petit Racloir	
+	145	68	137	105	621,5	EO	↘	40		Piece retouchée	
+	146	79	124	102	618,5	NS	.	35	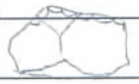	Petit racloir	
+	147	92	131	102	618,5	EO		30		Petite lame retouchée	
+	148	89	140	101	617,5			30		Piece retouchée	
+	149	95	137	100	616,5	EO		25		Piece retouchée	
+	150	93	145	102	618,5	EO		27		Petite lame retouchée	
+	151	99	145	102	618,5			35		Piece retouchée	
+	152	58	128	106	620,5			25		Fragment de racloir	
+	153	65	138	105	621,5			30		Denticulée	
+	154	66	140	105	621,5			25		Denticulée	
+	155	77	142	105	621,5			22		Denticulée.	
+	156	81	135	106	622,5			25		Denticulée	
+	157	72	143	106	622,5	NS		30		Fragment de lame retouché	
+	158	[illegible]	[illegible]	105	621,5			20		Denticulée.	
+	159	59	117	105	621,5	NS	↘	36		Piece retouchée.	
+	160	58	[illegible]	108,5	625	NS		35		Denticulé	
+	161	68	121	106	622,5			28		Denticulé	
+	162	74	126	106	622,5	NS		38		Denticulé	
+	163	76	134	109	625,5			25		Denticulé	

87

Ferrassie

Le : 19/8/70

Page N° 8

CARRÉ : 1

Visées faites sur : △ = 516,5

.... = ... m.

Nom et adresse : ......

Observations : (Y a-t-il un relevé, commentaire, photo, ... ?)

M ou A	N°	X	Y	Z	△ ± Z	Orien-tation	Pen-dage	Dimensions	Croquis forme	Désignation et nature	Niveau ou sol
X	164	83	132	110	626,5			30		Denticulé	
X	165	81	141	109	625,5			30		Denticulé	
X	166	78	146	108	624,5	EO		40		Fragment de lame retouché	
X	167	73	142	109	623,5			30		grattoir ou racloir	
	168	62	151	110	626,5			20		Dent pré[illegible] Humaine	+
X	169	30	120	106	622,5			25		Dent	
X	170	52	114	106	621,5	EO		26		Denticulé	
X	171	61	121	107	623,5			28		Denticulé	
X	172	68	128	108	624,5			30		Denticulé	
X	173	98	125	110	626,5			25		Denticulé	
X	174	86	127	110	626,5			20		Denticulé	
X	175	48	110	108	624,5	NS		28		Denticulé	
X	176	48	117	108	624,5	NS		30		Denticulé	
X	177	52	116	109	625,5	EO		40		lamelle retouch[illegible]	

516,5
+ 118
634,5

Le : 2018 / 7 / 0

CARRÉ : 1

Nom et adresse :

Visées faites sur : △ = 516,5

= m.

Observations : (Y a-t-il un relevé, commentaire, photo, ?)

M ou A	N°	X	Y	Z	△ ± Z	Orien-tation	Pen-dage	Dimensions	Croquis forme	Désignation et nature	Niveau ou sol
	178	60	106	112	628,5	EO		40		~~[illegible]~~ eclat	Xa sous jacent a
	179	62	112	111	627,5			25		Denticulé	L2 bJ
	180	59	121	111	627,5			20		Eclat concassage	
	181	62	123	112	628,5	NO SE		38	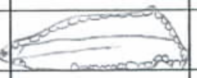	Lame retouchée	
	182	68	121	111	627,5			25		Denticulé	
	183	71	133	117	633,5			25		Denticulé	
	184	59	115	111	627,5			15		Fragment de lame retouchée	
	185	60	112	113	630,5			17		Fragment de lame retouché	
	186	70	118	117	633,5			16		Dent	
	187	56	108	111	627,5			15		Bord de lame retouché	
	188	62	112	113	630,5					Eclat retouché	
	189	54	90	113,5	631	EO	↘			Lame	
	190	93	129	114	631,5	EO	↓			Lame	
	191	56	103	112	629,5					Pièce retouchée	
	192	81	105	118	634,5			25		Pièce retouchée	
	193	95	128	126	642,5			35		fragment lame denticulée gratt.	X b
	194	76	152	121	637,5			30		éclat sans ret.	"
	195	54	134	133	649,5		↓	45		Hawaiian radar? ou fragment de nucleus	
	196	61	133	132	648,5			25		galet roulé	
	197	58	142	130	646,5			30		Eclat	
	198	69	138	130	646,5			60		os	
	199	70	145	127	643,5			35		Eclat	
	200	85	151	127	643,5	EO		50		os	
	201	90	149	126	642,5	EO				os	

132 F 70

Le : 20 / 8 / 70

Page N° 9

CARRÉ : 1

Nom et adresse :

Visées faites sur : △ = 516,5

= m.

Observations : (Y a-t-il un relevé, commentaire, photo, ?)

M ou A	N°	X	Y	Z	△ ± Z	Orientation	Pendage	Dimensions	Croquis forme	Désignation et nature	Niveau ou sol
+	178	60	106	628,5	628,5	E O		40		Eclat	x a sous jacent a
+	179	62	112	111	627,5			25		Denticulé	L2bJ
+	180	59	121	111	627,5			20		Eclat concassage	}
+	181	62	123	112	628,5	NO SE		38	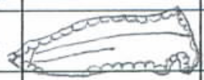	Lame retouchée	
+	182	68	121	111	627,5			25		Denticulé	
+	183	71	133	119	633,5			25		Denticulé	
+	184	59	115	111	627,5			15		Fragment de lame retouchée	
+	185	60	112	113	629,5			17		Fragment de lame retouchée	
+	186	70	118	117	633,5			16		Dent	
+	187	56	108	111	627,5			15		Bout de lame retouché	
+	188	62	112	113,5	629,5					Eclat retouché	
+	189	54	90	113	630	E O	↘			Lame type Chabel Penon	
+	190	93	129	114	630,5	E O	↓			Lame	
+	191	56	103	112	628,5					Piece retouchée	
+	192	81	105	118	634,5			25		Piece retouchée	
+	193	95	128	126	642,5			35		fragment de lame denticulée grad.	x b
+	194	76	152	121	637,5			30		Eclat sans retouche	"
+	195	54	134	133	649,5		↓	45		Mauvais racloir ? ou fragment nucleus	}
+	196	61	133	132	648,5			25		galet roulé	
+	197	58	142	130	646,5			50		Eclat	
+	198	69	138	130	646,5			60		os	
+	199	70	145	127	643,5			35		Eclat	
+	200	85	151	127	643,5	E O		50		os }	
+	201	90	149	126	642,5	E O				os }	

133 F 70

Le : 21 / 8 / 70

Page N° 10

CARRÉ : 1

Nom et adresse :

Visées faites sur : △ = 516,5

= m.

Observations : (Y a-t-il un relevé, commentaire, photo, ?)

M ou A	N°	X	Y	Z	△ ± Z	Orientation	Pendage	Dimensions	Croquis forme	Désignation et nature	Niveau ou sol
+	202	61	153	125	641,5			50		Eclat	Xb
+	203	61	152	125	641,5			30		Eclat	
+	204	79	127	132	648,5	EO?	↘	55	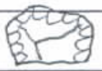	Racloir discoïde épais	
+	205	73	133	133	649,5	EO	→	30		eclat	
+	206	67	140	131	647,5	NS		80		os	
+	207	62	147	128	644,5			40		éclat	
+	208	46	155	128	644,5	NS	→	50		eclat laminaire retouché ?	
+	209	55	155	128	644,5	EO		50		os	
+	210	58	152	130	646,5	EO		70		os côte 2	
+	211	74	145	132	648,5			40		éclat	
+	212	67	148	132	648,5	EO		70		os	
+	213	47	154	133	649,5			45		Eclat	
+	214	70	167	139	655,5					Eclat	
+	215	88	160	132	648,5			40		os	
+	216	55	148	138	654,5			40		Eclat	
+	217	52	147	137	653,5			100		os	
+	218	90	143	136	652,5			40		Eclat	
+	219	64	139	141	657,5			60 x 40		fragment cranien (espèce ???	+
+	220	53	132	142	658,5			35		éclat	
+	221	57	150	147	663,5	EO	↓	35		eclat	Zone plus
+	222	65	152	147	663,5	EO	↓	60		eclat	sombre
+	223	58	135	148	664,5	EO	↘	30		eclat	foyer?
+	224	60	135	149	665,5	EO	→	33		fragm. lame	charbon
+	225	64	137	149	665,5	EO	↓	40		éclat	

Ferrassie 72

Le : 12/8/72

Page N° 3

CARRÉ : 1

Nom et adresse :

Visées faites sur : △ = 570,5

= m.

Observations : (Y a-t-il un relevé, commentaire, photo, ?)

M ou A	N°	Y	X	Z	△ ± Z	Orientation	Pendage	Dimensions	Croquis forme	Désignation et nature	Niveau ou sol
	49	94	27	45	615,5	EW	4	4x2	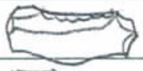		L2b1
	50	86	33	50	620,5	EW	0	4x3	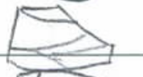		L2b1
	51	81	37	49	619,5	EW	0	4x3	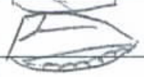		L2b1
	52	79	19	37	607,5	EW	0	7x4		Os (4 morceaux)	L2b1
	53	84	18	40	610,5	EW	2-3	8x2		Os	L2b1
	54	84	18	43,5	614	EW	4	5x3	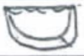		L2b1
	55	100	30	45	615,5	EW	0	5x3	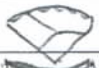		L2b1
	56	100	25	42	612,5	EW	0	3x2	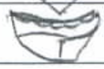		L2b1
	57	72	20	46,5	617	EW	0	7x2		Os	L2b1
	58	69	20	49	619,5	EW	0	5x3	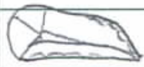		L2b1
	59	38	30	50	620,5	EW	0	5x3	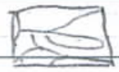		L2b1
	60	64	24	41	611,5	EW	2	9x2		Os	L2b1
	61	62	20	39	609,5	EW	1-2			Os (plusieurs)	L2b1
	62	61	18	49	619,5	EW	0-1	7x3	}	Silex (2 morceaux)	L2b1
	63	63	18	49	619,5	EW	0-1	7x3		(cassé par piquet?)	L2b1
	64	64	36	51	621,5	NS	0	6x4	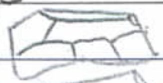		L2b2
	65	71	30	50,5	621	EW	1	4x3	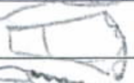		L2b2
	66	63	22	55	625,5	NS	2	3½x2½	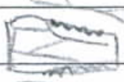		L2b2
	67	74	30	52,5	623	EW	1	4x2	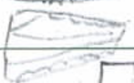		L2b2
	68	63	34	53	623,5	EW	4		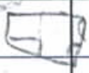		L2b2
	69	89	27	52,5	623	?	?	2x1	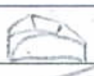		L2b2
	70	66	40	52	622,5	NS	0-1	6x4	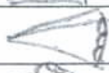		L2b2
	71	67	33	54,5	625	EW	2		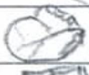		L2b2
	72	67	38	54	624,5	–	0	3x3	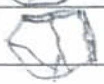		L2b2

La Ferrassie 72	Le : 16/8/72	Page N° 4
		CARRÉ : 1
Nom et adresse :		Visées faites sur : △ = 570,5
		.... = ... m.

Observations : (Y a-t-il un relevé, commentaire, photo, ... ?)

M ou A	N°	Y *	X *	Z	△ ± Z	Orientation	Pendage	Dimensions	Croquis forme	Désignation et nature	Niveau ou sol
	73	63	40	53	623,5	NS	0-1	4x4	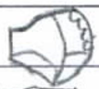		L2b2
	74	77	36	53	623,5	NS	4	3x2	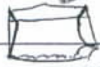		
	75	70	32	57	627,5	NS	0	3x1	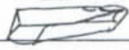		
	76	75	37	55,5	626	EW	1-2	4x2	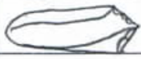		
	77	73	41	55	625,5	NS	0-1	3x3	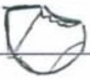		
	78	52	32	52	622,5	NS	1	4x2		Nucleus	
	79	63	38	53,5	624	NS	0-1	3x2	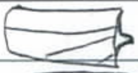		
	80	38	33	49,5	620	NS	0	5x2	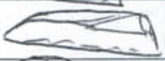		L2b1 (sic
	81	67	40	55	625,5	EW	0	3x3	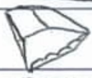		L2b2
	82	64	38	55	625,5	EW	0-1	6x4	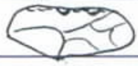		
	83	49	40	52	622,2	EW	2	4x3	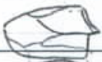		
	84	71	27	57	627,5	EW	0	3x2	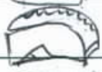		
	85	66	24	57	627,5	EW	0	3x2	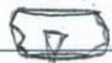		
	86	60	21	57	627,5	EW	0	3x2	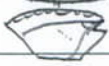		
	87	61	26	58	628,5	EW	4	3x2	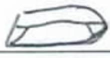		
	88	60	34	56	626,5	EW	0	4x3	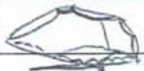		
	89	53	29	55	625,5	NS	3-4	2x2	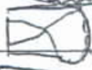		
	90	52	18	55	625,5	NS	0	3x2	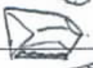		
	91	56	31	56	626,5	NS	0	4x3	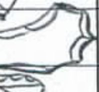		
	92	53	33	56	626,5	EW	3-4	3x2	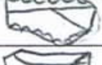		
	93	58	35	56	626,5	EW	0-1	3x3	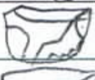		
	94	50	39	55,5	626	EW	2	3x2	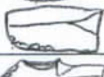		
	95	69	43	58	628,5	EW	2	4x2½	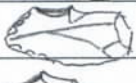		
	96	62	35	60,5	631	EW	4	3x1	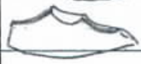	Fragment de denticulé	

La Ferrassie 72

Le : 16, 8, 72

Page N° 5

CARRÉ : 1

Nom et adresse :

Visées faites sur : △ = 570,5

= m.

Observations : (Y a-t-il un relevé, commentaire, photo, ?)

M ou A	N°	Y (~~X~~) *	X (~~Y~~) *	Z	△ ± Z	Orientation	Pendage	Dimensions	Croquis forme	Désignation et nature	Niveau ou sol
	97	58	37	58	628,5	NS	1	4x2			L262
	98	59	39	56,5	627	—	0	3x3			↓
	99	42	28	58	628,5	EW	4	3x2			↓
	100	70	17	51	621,5	EW	2	3x3		Eclat retouché	↓
	101	69	16	55,5	626	EW	2	3x2			↓
	102	52	19	57	627,5	EW	4	3x3		Petit grattoir	↓
	103	56	34	58	628,5	EW	0	3x3			↓
	104	53	40	58	628,5	EW	1-2	3x3			↓
	105	47	29	55,5	626	EW	3-4	2x2			↓
	106	49	30	57	627,5	NS	0	4x1½			↓
	107	53	30	56,5	627	—	0	3x3			↓
	108	48	35	55,5	626	NS	0-1	5x2			↓
	109	62	21	58	628,5	EW	0-1	2x1			↓
	110	61	18	60	630,5	—	0	2x2		Petit Grattoir	↓
	111	67	26	59	629,5	EW	4	2x1			↓
	112	77	32	60	630,5	EW	1	2x½			↓
	113	69	24	59,5	630	NW/SE	4	2x1			↓
	114	51	32	57	627,5	—	0	2x2		Petit Grattoir	↓
	115	60	37	58	628,5	NS	—	4x3		Eclat retouché	↓
	116	51	39	58	628,5	EW	2	2x2			↓
	117	58	29	59,5	630	EW	4	3x2			↓
	118	52	40	58,5	629	EW	0	4x2			↓
	119	93	10	30	600,5	EW	0	6x3			L261
	120	94	10	32	602,5	EW	0	7x5			↓

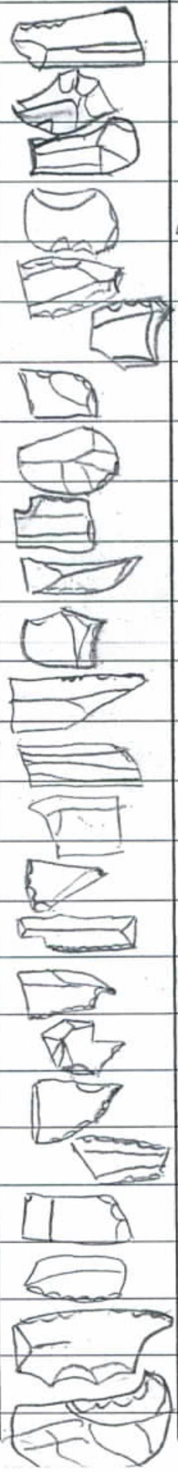

La Ferrassie 72

Le : 17/8/72

Page N° 6

CARRÉ : 1

Nom et adresse

Visées faites sur : △ = 570,5

= m.

Observations : (Y a-t-il un relevé, commentaire, photo, ?)

**

M ou A	N°	Y ~~X~~	X ~~Y~~	Z	△ ± Z	Orientation	Pendage	Dimensions	Croquis forme	Désignation et nature	Niveau ou sol
	121	91	10	33,5	604	NS	4	4x9	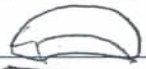	grand éclat	L261
	122	97	13	36	606,5	vertical		8x2	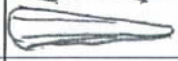	Os	↓
	123	90	9	37	607,5	vertical		5x3		Dent Bos	↓
	124	97	12	29	699,5	EW	1	11x2	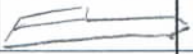	Os	↓

La Ferrassie 72	Le : 18/8/72	Page N° 7
		CARRÉ : 1
Nom et adresse :		Visées faites sur : △ = 570,5
		.... = ... m.

Observations : (Y a-t-il un relevé, commentaire, photo, ... ?)

M ou A	N°	X	Y	Z	△ ± Z	Orientation	Pendage	Dimensions	Croquis forme	Désignation et nature	Niveau ou sol
	125	10	83	33	603,5	EW	3	6x3		Dent Bos	L261
	126	2	74	31	601,5	EW	2	8½x5		Os (Bos ?)	
	127	13	92	38	608,5	NS	0	7x5		Fragment de silex	
	128	9	86	40	610,5	NS	1	5x3		Dent Bos	
	129	13	90	43,5	614	NS	2	4x4	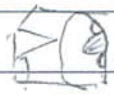		
	130	12	73	42	612,5	NS	1	5x3		Dent Bos	
	131	8	94	44	614,5	NS	3	5x3	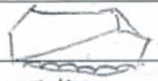	fragment de	
	132	4	89	44	614,5	NS	2	7x3	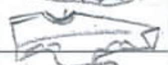	Lame retouché	
	133	14	83	44,5	615	EW	1-2	6x5	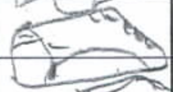	Éclat retouché	
	134	7	84	48	618,5	EW	3	5x3½	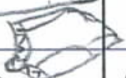		
	135	8	83	48	618,5	EW	0	5x3	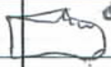	Grattoir	
	136	8	87	50	620,5	EW	0	3x2		Dent (Bos ?)	
	137	8	94	49	619,5	EW	4	5x2	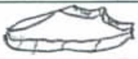		
	138	7	90	50	620,5	NS	3-4	2½x2½	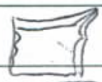		
	139	1	93	49	619,5	NS	3	3x2	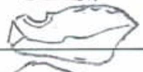		
	140	2	86	46	616,5	EW	3	3x2	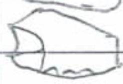	Eclat utilisée ?	
	141	8	89	52,5	623	EW	0	4x1		Dent (Bos ?)	
	142	8	93	51	621	NS	0	4x1	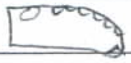	Fragment Lame	
	143	10	87	51,5	622	NS	0	5x2	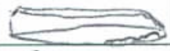	Lame	
	144	8	90	51,5	622	NS	0	4x3	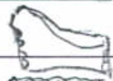		
	145	10	92	53,5	624	NS	0	3x2	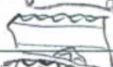		
	146	9	85	53,5	624	–	0	4x3	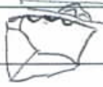		
	147										
	148										

FERRASSIE 72

Le : 18/8/72

Page N° 8

CARRÉ : 1

Nom et adresse :

Visées faites sur : △ = 570,5

= m.

Observations : (Y a-t-il un relevé, commentaire, photo, ?)

M ou A	N°	X	Y	Z	△ ± Z	Orientation	Pendage	Dimensions	Croquis forme	Désignation et nature	Niveau ou sol
	149	35	135	29	599,5	NE-SO	2-NO	4x3		éclat retouché	L2b1
	150	22	128	31	601,5	NE-SO	3-SE	6,5x3		fragm os	L2b1
	151	21	127	32,5	603	NE-SO	1-S	6x2		fragm lame	L2b1
	152	32	136	31,5	602	NE-SO	0	6x1,5		fragm d'os	L2b1
	153	27	123	32	602,5	NO-SE	2-SO	5x4		éclat retouché	L2b1
	154	28	127	34	604,5		0	3x2		" "	L2b1
	155	32	128	34	604,5	NE-SO	3-SO	5x3,5		fragm. quartz	" "
	156	36	135	32,5	603	N S	2-O	3x2		grattoir museau	" "
	157	38	129	35	605,5	N S	0	4x3,5	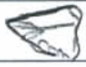	éclat retouché	" "
	158	31	125	33,5	604	E O	0	3x1		fragm. outil retouché	" "
	159	33	116	34	604,5	NE-SO	2-NO		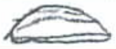	racloir latéral convexe	" "
	160	39	124	35	605,5	N S	3-E	3x4		fragm. racloir	" "
	161	34	133	34	604,5		0	3x2		éclat retouché	" "
	162	41	118	35	605,5		2-O	4,5x5	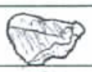	" "	" "
	163	39	113	36	606,5	EN-OS	4		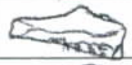	racloir	" "
	164	40	104	34	604,5		0	4,5x4,5	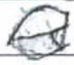	éclat retouché	" "
	165	32	109	35	605,5	E O	0	4,5x3		" "	" "
	166	32	119	37	607,5	NO-SE	2-NE	6x4		" "	" "
	167	41	100	40	610,5	E O	4	4x3,5		" "	" "
	168	35	122	36,5	607	E O	2-N	2,5x2		" "	" "
	169	21	118	35	605,5	NE-SO	2-NO	3x2		fragm racloir ?	" "
	170	26	140	33	603,5	N-S	1-N	3,5x4,5		éclat retouché	" "
	171	41	136	33	603,5	NESO	3-NE	2x2		fragm lame	" "
	172	42	123	39	609,5	NOSE	3-SE	4,5x2		denticulé	" "

LA FERRASSIE

Le : 18 / 8 / 72

Page N° 9

CARRÉ : 1

Visées faites sur : △ = 570,5

.... = ... m.

Nom et adresse :

Observations : (Y a-t-il un relevé, commentaire, photo, ... ?)

M ou A	N°	X	Y	Z	△ ± Z	Orientation	Pendage	Dimensions	Croquis forme	Désignation et nature	Niveau ou sol
	173	21	110	35	605,5			5x3		fragm coté	L2B1
	174	22	110	37	607,5	NO-SE	1-S	6x3		éclat retouché	〃 〃
	175	40	111	37,5	608		2-SE	5x6		fragm racloir	〃 〃
	176	34	113	40,5	611	NO-SE	1-NO	6x3,5		denticulé	〃 〃
	177	36	118	40,5	611	E-O	0	3,5x3		éclat retouché	〃 〃
	178	34	112	42	612,5	NO-SE	1-E	5x4		racloir	〃 〃
	179	34	132	38,5	609	NE-SO	2-S	4x3,5		racloir	〃 〃
	180	27	128	39,5	610			5x3		nucleus	〃 〃
	181	31	128	40	610,5	NE-SO	1-NO	5x4		racloir	〃 〃
	182	29	131	41	611,5	E-S	2-S	5x4,5		éclat retouché	〃 〃
	183	21	133	38	608,5	NO-SE	3-NE	4x2,5		grattoir en bout de lame	〃 〃
	184	23	126	40	610,5	N-S	2-S	4x2,5		éclat retouché racloir ?	〃 〃
	185	27	124	40,5	611	N S	0	4x2,5		fragm racloir	〃 〃
	186	27	140	36	606,5	E-O	3-N	4x2		racloir	〃 〃
	187	19	118	38,5	609	N-S	0	4x3		racloir	〃 〃
	188	40	127	41	611,5	N-S	0	4x3		racloir ?	〃 〃
	189	28	126	42	612,5	NO-SE	0	6x2,5		éclat retouché	〃 〃
	190	36	103	42	612,5	N S		6x4		nucleus	〃 〃
	191	35	108	43	613,5	NO-SE	0	6x4		racloir	〃 〃
	192	27	105	42,5	613	NO-SE	1-O	6x5		racloir	〃 〃
	193	18	120	41	611,5	NE-SO	1-SO	6x4		racloir	〃 〃
	194	40	122	45	615,5		0	4x4		éclat retouché	〃 〃
	195	38	38	41	611,5			3x2		éclat retouché	〃 〃
	196	33	102	48	618,5	E O	0	3,5x2,5		〃 〃	〃 〃

LA FERRASSIE 72	Le : 21/8/72	Page N° 10
		CARRÉ : 1

Nom et adresse :

Visées faites sur : △ = 570,5

= m.

Observations : (Y a-t-il un relevé, commentaire, photo, ?)

M ou A	N°	X	Y	Z	△ ± Z	Orientation	Pendage	Dimensions	Croquis forme	Désignation et nature	Niveau ou sol
	197	40	108	48	618,5	NS	2-SE	5x1,5		pointe chatelperon	L2b1
	198	39	110	47,5	618	EO	1-N	4x3,5		éclat retouché	L2b1
	199	21	120	43,5	614	EO	0	3,5x2		dent	" "
	200	28	115	46	616,5	E-O	1-O	5x1,8		lame	" "
	201	24	104	44	614,5		1-E	5x4,5		racloir ?	" "
	202	19	112	43	613,5	NS	0	3,5x3		racloir	" "
	203	18	115	44	614,5	NO-SE	0	6,5x3		éclat retouché	" "
	204	25	127	46,5	617	NS	0	4,5x2		" "	" "
	205	29	128	47	617,5	NE-SO	1-SO	6x3		fragm. racloir type ferrassie	" "
	206	30	105	50	620,5	NESO	2-NO	4x2,5		éclat retouché	" "
	207	31	109	51	621,5		2-NE	4x4,5		racloir	" "
	208	26	100	50	620,5	E-O	0	3,5x2,5		fragm racloir	" "
	209	27	24	48	618,5			3x2,5		burin	" "
	210	35	117	50	620,5	NO-SE	1-NO	4x1	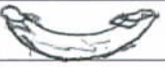	fragm dent ?	" "
	211	30	128	49	619,5	N-S	1-N	4,5x1,5		fragm lame	" "
	212	34	126	49	619,5	NO-SE	0	2,5x2		éclat retouché	" "
	213	35	112	52	622,5	N-S	0	2,5x1	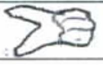	dent cervidés	" "
.	214	34	136	46	616,5	NS	1-N	5x5,5		racloir ?	" "
	215	34	133	48	618,5	EO	0	3x3		fragm lame	" "
	216	21	120	48	618,5	EO	1-N	4x3		racloir	" "
	217	33	98	52	622,5	NO-SE	0	4x2,5		éclat retouché	" "
	218	26	102	52	622,5	E-O	0	3,5x2,5		" "	" "
	219	21	112	50	620,5	NO-SE	0	2,5x2		fragm lame retouchée	" "
	220	16	107	36	606,5		1-S	5x5		nucléus	" "

LA FERRASSIE-72   Le : 21/8/72

Page N° 11

CARRÉ : 1

Nom et adresse :

Visées faites sur : △ = 570,5

.... = ... m.

Observations : (Y a-t-il un relevé, commentaire, photo, ... ?)

M ou A	N°	X	Y	Z	△ ± Z	Orientation	Pendage	Dimensions	Croquis forme	Désignation et nature	Niveau ou sol
	221	14	104	37,5	608	NO-SE	2, NE	6x4		racloir	L2B1
	222	13	104	38	608,5	N-S	2-S	8,5x4		" "	L2b1
	223	7	104	37,5	608	NE-SO	3-SE	8x4		racloir ?	L2b1
	224	15	16,5	37	607,5	E O	1-E	6x3		racloir type Ferrassie	L2b1
	225	12	120	32	602,5	N-S	2-N	6,5x4		racloir	L2b1
	226	7	124	34	604,5	E-O	0	3x2		dent	L2b1
	227	1	116	31,5	602	N-S	1-S	6x5		racloir	L2b1
	228	6	103	43	613,5	NE-SO	0	6,5x3		éclat retouché	L2b1
	229	6,5	107	43	613,5	N S	0	4,6x3		dent	L2b1
	230	3,5	102	46	616,5	E O	3-S	5x3,5		racloir	L2b1
	231	4	106	45	615,5	NO-SE	0	5x1,5		lame	L2b1
	232	2,3	107,5	44	614,5	N-S	0	4x2		dent	L2b1
	233	2	113	37	607,5		0	5x4		racloir	L2b1
	234	5	114	44,5	615	NO-SE	1-S	6,4x4		racloir	L2b1
	235	4	110	44,8	615,3	SO-NE	1-NE	5,1x2		racloir	L2b1
	236	3	122,5	36,5	607	NO-SE	3-SO	4,8x3		éclat retouché ?	L2b1
	237	5	118,5	38	608,5	N-S	1-SE	6x3		racloir	L2b1
	238	5	125	34,5	605	E O	0	5,8x2,5		éclat retouché	L2b1
	239	11	111,5	45,5	616	E O	1-O	5x3,5		nucléus	L2b1
	240	8	125	35,5	606	E O	4	6,8x5,5		gros éclat	L2b1
	241	3	111	43,5	614		1-NE	3x3		éclat retouché ?	L2b1
	242	16,5	125	51	621,5	NO-SE	0	4,5x2,5		éclat retouché	L2b1
	243	15	66	44	614,5	NEE-SOO	4	7,5x7		pierre contre le piquet	L2b1
	244										L2b1

FERRASSIE 73

Le : 14 / 8 / 73

Page N° 1

CARRÉ : 1

Nom et adresse :

Visées faites sur : △ = 583

= m.

Observations : (Y a-t-il un relevé, commentaire, photo, ?)

M ou A	N°	X	Y	Z	△ ± Z	Orientation	Pendage	Dimensions	Croquis forme	Désignation et nature	Niveau ou sol
x	1	8	24	36	619	EO	1E	13		os	L2b2
x	2	9	60	36	619	EO	2N	6		dent Bovidé	—
x	3	12	66	36	619	EO	3S	6		fragm. lame ret	—
x	4	8	72	44	627	EO/NS	0	6		fragm. lame ret	—
x	5	10	135	21	604	EO	0	6		grattoir	—
x	6	10	135	21	604	EO	0			frag. de racloir	—
x	7	6	126	21	604		1			frag os plat	—
x	8	7	120	25	608	EO	0	4 -		frag. racloir	—
x	9	8	111	32	615	NS	0	5		frag. racloir	
x	10	6	112	32	615	EO	2	3 1/2		éclat retouché	—
x	11	7	110	32	615	EO	0	3		" "	—
x	12	4	91	33	616	OE	0	5		grattoir	—
x	13	4	55	40	623	EO	0	28		pièce retouchée	-
x	14	6	60	38	621	EO	sur	chant 6 cm -		frag. racloir	—
x	15	7	62	38	621	NS/EO	0	55		lame épaisse retouch.	—
x	16	14	64	37	620	EO	4	4	pièce	denticulée retouchée	—
x	17	5	69	36	619	EO	1 Est	5,5		racloir ?	—
x	18	21	34	45	628	EO	0	3	pièce à	retouches Dufour	—
x	19	4	37	44	627	EO	4	1,5	ravivage d'	un nucléus	—
x	20	6	48	45	628					éclat	—
x	21	4	54	40	623	EO	1 E.	35	grattoir co	ncave et encoche	—
x	22	11	61	44	627	EO	sur	chant 4 cm		grattoir	—
x	23	14	67	48	631	EO	"	"	3 pièce	à retouches abruptes	-

FERRASSIE 73

Le : 14 / 8 / 73

Page N° 2

CARRÉ : 1

Visées faites sur : △ = 583

= m.

Nom et adresse :

Observations : (Y a-t-il un relevé, commentaire, photo, ?)

M ou A	N°	X	Y	Z	△ ± Z	Orientation	Pendage	Dimensions	Croquis forme	Désignation et nature	Niveau ou sol
											L²b2
x	24			39	622				ramissage		
x	25			39	622				"		—
x	26	2	136	14	600		0			percuteur rond	—
x	27	11	129	23	606		3			pièce retouchée	—
x	28	8	115	29	612	NO/SE		5,5		" "	—
x	29	10	109	34	617			5		" "	—
x	30	31	125	38	621		0			frag. lame retouchée	—
x	31	15	33	54	637	NS	0	3,5		Pièce à retouche remarquable	
x	32	2	48	56	639	NS	25	4		Pièce à retouche abrupte	
x	33	1	54	55	638	EO	4	2,5		pièce retouchée longue	
x	34	10	120	35	618	EO	2	6		" " noire	
x	35	12	120	35	618		1	4		" " claire	
x	36	20	118	39	622			4		morceau d'os arrondi	
x	37	20	125	33	616					pièce à petites dents	
x	38	20	127	33	616		1	4		pièce retouchée	
x	39	20	134	34	617		3	4,5		ravivage de nucléus	—
x	40	15	140	23	606		1	3		pièce retouchée	
x	41	18	115	38	621		1	2,5		fragment pointe châtelperron	L2b2
x	42	32	62	49	632	NS	2	2		pièce retouchée	
x	43	19	139	27	610					" "	
x	44	11	135	27	610					" "	
x	45	5	129	23	606	OE				extrémité articulation	
x	46	16	128	38	621					éclat retouché	
x	47	11	128	30	613						

Ferrassie 73

Le : 16 / août

Page N° 3

CARRÉ : 1

Nom et adresse :

Visées faites sur : △ = 583

= m.

Observations : (Y a-t-il un relevé, commentaire, photo, ?)

M ou A	N°	X	Y	Z	△ ± Z	Orientation	Pendage	Dimensions	Croquis forme	Désignation et nature	Niveau ou sol
x	48	8	117	38	621	NS	2 ouest			éclat retouché	L²b2
x	49	11	118	39	622	EO	1 sud			retouche en bout	
x	50	6	119	30	613					frag. racloir	
x	51	2	110	16	599					frag. nucleus	
x	52	5	107	34	617					frag. retouches abruptes	
x	53	6	111	36	619					éclat retouché	
x	54	10	113	37	620	NS				pièce retouchée	L²b2
x	55	9	107	37	620	NS				frag. lame retouchée.	
x	56	15	107	37	620	EO				pièce retouchée	
x	57	6	79	40	623	NS				frag. pièce à dos	
x	58	9	76	41	624					frag. de gravette ?	
x	59	18	78	43	626					frag. lame à pédoncule	
x	60	18	77	43	626					frag. retouche plate	
x	61	23	76	44	627					frag lame retouche Dufour	
x	62	10	147	20	603						
x	63	3	141	20	603					frag nucleus caréné	
x	64	8	139	30	613						
x	65	5	135	29	612						
x	66	1	[illegible]	18	601					nucleus discoïde	
x	67	6	134	48	621					pièce retouchée	
x	68	8	129	42	625					" "	
x	69	3	123	42	625					" "	
x	70	8	123	43	626					dent. [illegible]	
x	71	12	127	44	627					frag. lame retouchée	

F 73	Le : 16 août 73	Page N° 4
		CARRÉ : 1
Nom et adresse :		Visées faites sur : Δ = 583
		= m.

Observations : (Y a-t-il un relevé, commentaire, photo, ?)

M ou A	N°	X	Y	Z	Δ ± Z	Orientation	Pendage	Dimensions	Croquis forme	Désignation et nature	Niveau ou sol
x	72	19	124	44	627					pièce retouchée	L^2b^2
x	73	15	120	44	627					petit racloir	
x	74	19	123	45	628					frag. lame retouchée	
x	75	13	118	23	606					denticulé	
x	76	6	113	37	610					pièce à retouches } Dufour	
x	77	7	108	37	610					denticulé	
x	78	1	111	33	616					pièce retouchée	
x	79	4	101	33	616					pièce à retouches abruptes	
x	80	12	97	40	623					" " " "	
x	81	6	89	38	621					racloir double convergent	
x	82	4	95	35	618					encoche retouche abrupte	
x	83	3	97	32	615					racloir	
x	84	22	94	40	623					racloir denticulé	
x	85	24	89	40	623					pièce retouchée	
x	86	34	86	41	624					" "	
x	87	5	78	39	622					" "	
x	88	11	82	41	624					frag. racloir	
x	89	12	78	43	626					pièce à retouche inverse	
x	90	18	82	43	626					lame retouchée } Dufour	
x	91	29	77	44	627					denticulé	
x	92	4	140								L2b
x	93	4	140								L2b
x	94	4	140								L2b
x	95	4	140								L2b

F 73	Le : 1 8 / 73	Page N° 5
Nom et adresse :		CARRÉ : 1
		Visées faites sur : △ = 583
		= m.

Observations : (Y a-t-il un relevé, commentaire, photo, ?)

M ou A	N°	X	Y	Z	△ ± Z	Orientation	Pendage	Dimensions	Croquis forme	Désignation et nature	Niveau ou sol
x	96					pièces groupées					L_2b
x	97					en paquet autour					"
x	98					du point					"
x	99					5 - 140 hauteur				comprise entre	"
x	100									22 et 33	"
x	101	5	140	33	616					éclat retouche Dufour	
x	102	5	140	33	616					pointe levallois	~~Xb~~ L_21
x	103	3	85	43	626				~~Xb~~ L_2b	pièce à retouche Dufour	
x	104	13	89	44	627					lame retouchée	
x	105	7	89	42	625					frag. lame retouchée	
x	106	11	84	44	620					éclat retouché	
x	107	17	93	43	626					lame retouches inverses	
x	108	9	98	39	622					pièce à dos -	
x	109	30	95	45	628					pièce à encoche	
x	110	10	105	39	622					pièce à retouches Dufour	
x	111	16	110	44	627					galet denticulé	
x	112	42	90	46	629				frag.	dent	
x	113	32	105	44	627					pièce retouchée	
x	114	29	113	44	627					" "	
x	115	40	105	43	626					" "	
x	116	42	112	43	626				denticulée à	retouches abruptes	
x	117	39	126	37	610					pièce retouchée	
x	118	43	128	37	610					" "	
x	119	41	123	38	611					" "	

F. 73	Le : 17 août	Page N° 6
		CARRÉ : 1
Nom et adresse :		Visées faites sur : △ = 583
		= m.

Observations : (Y a-t-il un relevé, commentaire, photo, ?)

M ou A	N°	X	Y	Z	△ ± Z	Orientation	Pendage	Dimensions	Croquis forme	Désignation et nature	Niveau ou sol
+	120	-1	145	25	608					grattoir denticulé	~~X~~ b2b
+	121	19	127	36	619					ravivage de nucléus	
+	122	12	132	38	621					racloir triple	
+	123	2	122	36	619					pièce retouchée	
+	124	37	124	38	621					frag dent	
+	125	38	125	44	627					pièce retouchée	
^	126	32	124	44	627					grattoir	
x	127	37	122	46	629					grattoir caréné	
x	128	13	114	40	623					éclat retouché	
x	129	18	112	43	626					" "	
x	130	7	105	38	621					lame retouchée	
x	131	9	100	39	622					éclat retouché	
x	132	29	116	43	626					racloir double	
x	133	30	110	45	628					éclat retouches abruptes	
x	134	23	106	44	627					lame retouchée	
x	135	13	100	42	625					frag. lame retouches abruptes	
x	136	22	101	47	630					" " " Dufour	
x	137	3	65	47	630					pièce à dos	
x	138	5	81	44	627					pièce pédonculée retouches abruptes	
x	139	3	102	38	621					frag. lame retouchée	L2b1
x	140	5	103	38	621					pièce retouchée	" "
x	141	4	109	42	625					" "	" "
x	142	9	115	44	627					frag. nucléus retouché	" "
x	143	13	105	43	626					éclat retouché	X. b

Page N° 7

Le : / /

CARRÉ :

Nom et adresse :

Visées faites sur : △ = 583

= m.

Observations : (Y a-t-il un relevé, commentaire, photo, ?)

M ou A	N°	X	Y	Z	△ ± Z	Orientation	Pendage	Dimensions	Croquis forme	Désignation et nature	Niveau ou sol
x	144	18	103	43	626					lame à retouches inverses	~~X b~~ L2b
x	145	17	109	42	625					base de lame retouchée	
x	146	21	113	44	627					lame retouchée	~~X b~~
x	147	20	116	44	627					éclat retouché	~~X b~~
x	148	2	128	36	619					éclat retouché	L2b2
x	149	1	124	34	617					grattoir bec ?	L2b2
x	150	-3	154	24	607					Racloir concave	L2b2
x	151	1	166	22	605					grattoir s. lame	~~X b~~ L2b
x	152	2	161	20	603					racloir ?	~~X b~~
x	153	7	162	20	603	EO	0	3		grattoir	L2bj
x	154	12	163	23	606	WS	0	3		fragm. lame ret	}
x	155	12	169	23	606	EO	4	4		denticulé ?	
x	156	16	171	22	605	EO	4	3,5		denticulé	
x	157	27	152	23	606			3,5		denticulé	
x	158	25	161	32	605	NS	0	5		fragm. racloir double	
x	159	40	145	33	616	NS	15	4		grattoir caréné !	
x	160	35	159	30	613	EO	4	3		grattoir	
x	161	38	149	29	612			5,5		racloir conv ?	
x	162	32	169	23	606	EO	1.0	4,5		racloir dent.	
x	163	43	172	31	604	NS	0	5		éclat lamin	
x	164	21	120	55	638	EO	0	3,5		denticulé	L2b2
x	165	13	151	29	612					racloir abrupt	L2bJ
x	166	20	156	27	610					grattoir circulaire	}
x	167	20	162	27	610					fragm racloir double	

La Ferrassie 73

Le : 21 / 8 / 73

Page N° 8

CARRÉ : 1

Nom et adresse :

Visées faites sur : △ = 583

= m.

Observations : (Y a-t-il un relevé, commentaire, photo, ?)

M ou A	N°	X	Y	Z	△ ± Z	Orientation	Pendage	Dimensions	Croquis forme	Désignation et nature	Niveau ou sol
x	168	26	145	28	611	EO	0	3		base de lame	L2bj
x	169	24	162	26	609	EO	0	3		lame ret fine semi abrupte	
x	170	26	167	33	616	EO	2E	3		base lame retouch	
x	171	35	145	39	622	EO	0	4		Racloir encoche	
x	172	34	154	36	619	EO	0	2,5		fragm racloir convergent	
x	173	34	159	33	616	EO/NS	0	3		fragm lame ret. tronquée	
x	174	35	166	28	611	EO	1E	3,5		lame a semi pédoncule	
x	175	39	148	39	622	NS	0	4,5		os plat	
x	176	39	153	37	620	EO	1E	2		os plat	
x	177	39	162	31	614	EO	1E	2,5		petit racloir	
x	178	38	169	27	610	EO	0	4,5		racloir	
x	179	13	159	29	612	EO	0	5		lame retouchée	
x	180	38	152	33	616	NS	20	2		os plat	
x	181	37	155	28	611	NS	0	3,5		racloir concave	
x	182	36	161	29	612	EO	2E	4,5		fragm nucléus	
x	183	21	166	37	620	EO	0	2		grattoir	
x	184	10	171	26	609	NS	2E	4		éclat	
x	185	13	165	31	614	NS	1E	3,5		racloir	
x	186	24	172	32	615	NS	0	2		os plat	
x	187	23	167	33	616	EO	0	4		Denticulé ?	
x	188	32	150	39	622	EO	4	3		lame ret.	
x	189	40	158	37	620	NS	0	3,5		grattoir ?	
x	190	39	163	38	621	NS	0	2,5		?	
x	191	36	170	35	618	NS	0	2		lame ret.	

La Ferrassie 1973

Le : 22/ 8 /73

CARRÉ : 1

Nom et adresse :

Visées faites sur : △ = 583

= m.

Observations : (Y a-t-il un relevé, commentaire, photo, ?)

M ou A	N°	X	Y	Z	△ ± Z	Orien-tation	Pen-dage	Dimensions	Croquis forme	Désignation et nature	Niveau ou sol
+	192	-3	147	29	612	?	0			dent herbivore	L2bj
+	193	4	160	30	613	NS	0			fragm. nucléus	↓
+	194	11	158	29	612	EO	10			racloir	
+	195	15	145	33	616	NS	1N			base lame pédonculée	
+	196	30	135	35	618	NS	0			pièce retouchée	
+	197	30	151	32	615	EO	10			pièce retouchée	
+	198	37	149	41	624	EO	0			fragm. lame ret	
+	199	36	156	43	626	EO	20			denticulé	
+	200	16	144	33	616					fragm. lame tronquée	
+	201	16	148	34	617						
+	202	22	148	38	621					dent renne	
+	203	21	156	37	620						
+	204	31	134	42	625						
+	205	29	139	42	625						
+	206	29	145	40	623						
+	207	30	146	41	624						
+	208	-1	142	31	614					lame retouchée	
+	209	-9	151	37	620					nucléus prototype de caréné	
+	210	-8	155	37	620					grattoir s. lame	
+	211	5	152	32	615					jaspe brun retouché	
+	212	5	160	31	614					denticulé	
+	213	17	145	33	618					éclat retouche Dufour	
+	214	33	135	44	627					denticulé bec	
+	215	30	140	46	629					fragm. nucléus ?	

La Ferrassie 73	Le : 22/8/73	Page N° 10
Nom et adresse :		CARRÉ : 1
		Visées faites sur : △ =583
		= m.

Observations : (Y a-t-il un relevé, commentaire, photo, ?)

M ou A	N°	X	Y	Z	△ ± Z	Orientation	Pendage	Dimensions	Croquis forme	Désignation et nature	Niveau ou sol
x	216	29	148	34	617					fragment nucléus	[2b]
x	217	-3	151	30	613					eclat retouche type Dufour	
x	218	7	152	31	614					eclat denticulé retouché	
x	219	6	154	30	613					dent renne	
x	220	5	160	29	612					fragm lame retouche type Dufour	
x	221	16	149	38	621					racloir	
x	222	3	155	31	614					fragm Nucléus	
x	223	5	162	35	618					piece retouchée	
x	224	16	152	37	620					racl. concave sur denticulé	
x	225	12	153	36	619					phalangette	
x	226	-7	143	27	610		4			dent bovidé ?	
x	227	-10	144	30	613		4			grattoir circulaire s. nucléus	
x	228	-1	149	32	615	NS	15			fragm lame	
x	229	0	153	33	616	EO	0			denticulé ret.	
x	230	0	163	33	616	EO	15			grattoir	
x	231	0	144	37	620	/	25			racloir grattoir ?	
x	232	1	149	37	620	/	1E			grattoir denticulé ?	
x	233	-13	148	43	626	EO	10			racloir	M1
x	234	-6	151	40	623	NS	15			denticulé semi abrupt	
x	235	2	151	41	624	?	0			grattoir caréné ?	
x	236	0	145	43	626		25			grattoir denticulé	
x	237	-3	148	42	628		20			retouche plate	
x	238	-10	151	43	626		0			grattoir denticulé	
x	239	11	148	43	626		0			base de lame retouch	

La Ferrassie 73

Le : 23 / 8 / 73

Page N° 11

CARRÉ : 1

Nom et adresse :

Visées faites sur : △ = 583

= m.

Observations : (Y a-t-il un relevé, commentaire, photo, ?)

M ou A	N°	X	Y	Z	△ ± Z	Orien-tation	Pen-dage	Dimensions	Croquis forme	Désignation et nature	Niveau ou sol
x	240	-10	154	44	627					fragm. Chatelperron	M1
x	241	12	153	45	628					?	
x	242	17	141	47	630					fragm. Chatelperron	
x	243	37	144	70	653					lame	
x	244	30	151	73	656					os	
x	245	24	159	41	654					silex	
x	246	-2	160	79	662	NS		X		os (cranien?)	M2
x	247	2	156	74	657		30	x		os idem	
x	248	36	147	78	661		sur chant			silex retouché	
x	249	-10	115		600					Racloir	L2b
x	250	-25	115		600					id	"
x	251	-20	110		601					id	"
x	252	-30	105		600					id	"
x	253	-35	108		600					dent	"
x	254	-30	110		602					racloir	"
x	255	-70	105	20	603					grattoir.	"
x	256	-15	108		602					racloir	"
x	257	-35	120		600					"	"
x	258	-40	100		"					"	"
x	259	-25	125		600					fgt "	"
x	260	-5	115		605					racloir	"
x	261	-30	120		605					"	"
x	262	-5	108		605					"	"
x	263	?	?		603					"	"

F73

Le : / /

Page N° 12

CARRÉ : 1

Nom et adresse :

Visées faites sur : △ = 583

= m.

Observations : (Y a-t-il un relevé, commentaire, photo, ?)

M ou A	N°	X	Y	Z	△ ± Z	Orientation	Pendage	Dimensions	Croquis forme	Désignation et nature	Niveau ou sol
x	264	-80	130		608					Nucleus	L2b
x	265	-7	128		609					Grattoir	"
x	266	-3	115		609					Denticulé	"
x	267	-10	125		610					scalariforme	"
x	268	-27	112		612					racloir	"
x	269	-22	107		609					racloir	L2ba
x	270	-20	135		606					racloir	"
x	271	-15	130		614					éclat retouché	"
x	272	-2	127		606					Nucleus	L2ba
x	273	-35	129		615					racloir	"
x	274	-38	114		615					racloir	"
x	275	-22	125		616					racloir	L2ba
x	276	-10	125		616					grattoir	L2ba
x	277	-19	118		616					Coche (f[illegible])	L2ba
x	278	-27	100		610					nucleus	L2ba
x	279	-12	120	37	620					pte Chatp.	L2ba
x	280	-12	107	38	621					" fgt	L2ba
x	281	-8	120	38	621					fgt racloir	L2ba
x	282	-49	112	36	619					lame	"
x	283	-27	127	37	620					dent	M1
x	284	-29	128	37	620					frag pte Chatelperon	M1
x	285	-13	128	37	620					fragm lame retouchée	M1
x	286	-16	120	38	621					frag pte Chatelp	M1
x	287	-18	135	38	621					éclat retouché	M1

F . 73	Le 24 / 8 / 73	Page N° 13
		CARRÉ : 1

Nom et adresse :

Visées faites sur : △ = 583

= m.

Observations : (Y a-t-il un relevé, commentaire, photo, ?)

	M ou A	N°	X	Y	Z	△ ± Z	Orientation	Pendage	Dimensions	Croquis forme	Désignation et nature	Niveau ou sol
	x	288	-12	128	40	623					racloir	M1
	+	289	?	?		± 610					〃	L2b
	+	290	?	?		± 610					éclat	〃
	+	291	-15	128	41	623					fragm pte chatelp	surface M1
	+	292	-10	145	44	627					coche	M1
	+	293	-60	110	35	618					pièce à retouche abrupte	L2b
	√	294	-8	120		625					racloir	M1
x		295	30	141	78	661			x		côte	M2
x		296	28	140	78	661			x			M2
x		297	27	141	79	662			x			M2
x	x	298	15	139	78	661			x			
x	I	299	12	140	77	660			x			
x	F	300	13	148	81	664			x			
x H	I	301	10	151	80	663			x			
		302	0	152	81	664					silex	
x		303	28	141	78	661	E-O	4	x		os ✓	
x		304	28	141	79	662	E-O	4	x		os ✓	
x		305	33	140	76	659	E-O	4	x		os ✓	
x		306	36	137	77	660			x	couronne en bas M	molaire	x
x		307	29	139	80	663			x		os C	
x		308	29	139	80	663			x		os C	
x		309	29	139	80	663			x		os ✓	
x		310	29	139	80	663			x		os VERTEBRE	
x		311	26	140	81	664			x		os côte	

[illegible] identif. AGD

F. 73

Le : 27 / 8 / 73

Page N° 14

CARRÉ :

Nom et adresse :

Visées faites sur : △ = 583

= m.

Observations : (Y a-t-il un relevé, commentaire, photo, ?)

	M ou A	N°	X	Y	Z	△ ± Z	Orientation	Pendage	Dimensions	Croquis forme	Désignation et nature	Niveau ou sol
x		312	30	146	82	665	E O	O	x		os	M 2
x		313	34	138	77	660	E-O	O	x	racine vers est	prémolaire	
x		314	32	138	78	661	E-O	O	x	" "	canine	
x		315	32	137	79	662	E-O	O	x	racine vers ouest	molaire	
x		316	30	137	80	663	N-S	O	x	racine vers nord	molaire	
x		317	26	138	80	663		O	x		os V	
x		318	21	148	80	663			x		incisive	
x H F		319	20	150	81	664	E.O	O	x		os	
x H F		320	27	150	82	665	E O	O	x		os	
x T		321	31	156	82	665	N-S	O	x		os	
x		322	13	145	79	662	N-S	O	x		fragm côté	
x T		323	17	151	81	664	E-O	O	x		os	
x H F		324	26	150	82	665	E O	O	x		os	
x		325	6	164	82	665			x			
x		326	37	139	81	664	x		x		fragm crane	
x		327	39	149	80	663			x		" "	
x		328	40	144	80	663			x		" "	
x		329	40,5	144	80	663			x	sur champ	" "	
x		330	43,5	145	81	664			x		" "	
x		331	36,5	140	81	664			x		" "	
x		332	37	138	81	664			x		" "	
x		333	38	139	81	664			x		" "	
x		334	38	141	80,5	663,5			x		" "	
x		335	38	139	80	663			x		" "	

F-73	Le : 27/8/73	Page N° 15
Nom et adresse :		CARRÉ : 1
		Visées faites sur : △ = 583
		= m.

Observations : (Y a-t-il un relevé, commentaire, photo, ?)

	M ou A	N°	X	Y	Z	△ ± Z	Orientation	Pendage	Dimensions	Croquis forme	Désignation et nature	Niveau ou sol
x		336	39	142	80	663			X		fragm crane	M2
x		337	39	145	80	663			X		" "	
x		338	41	140	79,5	662,5			X		" "	
x		339	44	137	81	664			X		fragm crane	
x	I	340	37	139	81	664			X			
x	I	341	37	139	81	664			X			
x		342	32	135	80,5	663,5			X			
x		343	29	135	80,5	663,5						
x		344	29	135	80,5	663,5			X		V	
x		345	28	135	80,5	663,5			X			
x		346	26	135	80,5	663,5			X			
x		347	24	135	80,5	663,5			X		V	
x	I	348	28	153	83	666			X			
x	I	349	22	158	83	666			X			
x	I	350	19	158	83	666			X			
x		351	17	148	82	665			X			
H x	I	352	15	148	82	665			X			
x H	I	353	15	151	83	666			X			
		354	6	146	82	665					silex	
x		355	7	150	84	664	E-O		X		os FAUNE	F
x	F	356	8	157	82	665	N-S		X			
x	I	357	8	161	82	665	W-S		X			
x	I	358	10	161	82	665			X			
x	I	359	10	166	83	666			X			

F.73

Le : 28/8/73

Page N° 16

CARRÉ : 1

Nom et adresse :

Visées faites sur : △ = 583

= m.

Observations : (Y a-t-il un relevé, commentaire, photo, ?)

	M ou A	N°	X	Y	Z	△ ± Z	Orientation	Pendage	Dimensions	Croquis forme	Désignation et nature	Niveau ou sol
x	F	360	13	164	83,5	666,5		x			os	M2
x	T	361	14	166	83,5	666,5		x			os	
H x	F	362	22	152	83	666	E-O	x	12 cm		os	
H x	F	363	12	151	83	666	NE-SO	x			"	
H x	F	364	18	157	84	667	NO-SE	x			"	
H x	F	365	9	157	84	667	NO-SE	x			"	
x	T	366	15	153	82	665		x			amas de petits os	
x		367	15	142	82	665		x			os (3)	
x		368	22	141	82	665		x			os (3)	
x		369	30	143	81	664		x			os ✓	
x	T	370	18	146	84	667		x			"	
x	T	371	27	154	83	666		x			2 os	
x	F	372	24	154	83	666		x			os	
H x	T	373	19	159	85	668		x			2 os < horn small [illegible]	
H x	F	374	17	149	83	666	E-O	x			os	
H x	F	375	10	154	83	666	E-O	x				
H x	F	376	15	154	83	666	E-O	x				
H x	F	377	20	154	83	666	E-O	x				
x	T	378	7	162	82	665		x				
x		379	2	162	82	665		x				
x	T	380	6	171	82	665		x			fragment(s) os	
x	F	381	2	168	82	665		x			os	
H x	T	382	7	50	83	666	E O	x			2 frags of horn	
H x	F	383	15	48	84	667	E O	x				

Probably a horn. It has disappeared as it was sent to C. Howell.

FERRASSIE 73

Le : 28/ 8 /73

Page N° 17

CARRÉ : 1

Nom et adresse :

Visées faites sur : △ = 583

= m.

Observations : (Y a-t-il un relevé, commentaire, photo, ?)

strange coordinates

2 small fragments of bone

	M ou A	N°	X	Y	Z	△ ± Z	Orientation	Pendage	Dimensions	Croquis forme	Désignation et nature	Niveau ou sol
H x	F	384	9	52	84	667	E-O		x		os	M2
H x	F	385	13	52	84	667	E-O		x		"	
H x	F	386	19	50	84	667	E-O		x		"	
H x	I	387	19	50	84	667			x		~~amas~~ petits os<br>2 frag. bone	
x	I	388	27	140	83	666			x		amas petits os	
		389	31	139	83,5	666,5			x		3 frag os cranien	
x		390	33	137	mesure sur 82-	665			x		2 frag os "	
x		391	37	139	82	665			x		6 " " "	
x	I	392	5	146	82	665	E-O		x		os fragm cranien?	
x		393	9	149	83	666	E-O				os	
	I	394	9	149	83	666	E-O		x			
x	F	395	11	145	85	668	NO-SE		x			
H x	F	396	17	151	85	668	E-O		x			
x		397	20	153	85	668	E-O		x		coté	
x		398	21	150	86	669	~~E-O~~		x		silex	
x	I	399	17	159	85	668	E-O		x		os	
x	I	400	22	155	86	669	E-O		x			
x	F	401	-17	161	83	666			x			
x	I	402	36	152	87	670			x			
x	F	403	46	150	86,5	669,5			x			
x	I	404	43	149	86,5	669,5			x			
x		405	43	144	84	667			x		✓	
x		406	41	141	83	666			x			
x		407	37	141	85	668			x			

FERRASSIE 73

Le 29/8/73

Page N° 18

CARRÉ : 1

Nom et adresse :

Visées faites sur : △ = 583

= m.

Observations : (Y a-t-il un relevé, commentaire, photo, ?)

voir verseau de la feuille

	M ou A	N°	X	Y	Z	△ ± Z	Orien-tation	Pen-dage	Dimensions	Croquis forme	Désignation et nature	Niveau ou sol
x		408	31	143	85	668			x		os V	M2
x	I	409	25	139	84	667			x			
		410	20	136	78	661			x		silex	
x	F	411	19	142	86	669			x			
x		412	15	141	84	667			x		articulation Vol.	
x	I	413	5	144	85	668			x		os	
x	I	414	10	163	83,5	666,5		0	x			
x	I	415	13	163	83,5	666,5		0	x			
x		416	8,5	164	84	667		3	x			
x	I	417	6	163	84	667		0	x			
x	I	418	10	162	85	668		4	x			
x		419	35	141	85	668	NOSE	3-50	x		fragm crânien	
x	I	420	44	148	88	671			x		os	
x	F	421	41	148	86	669			x			
x	I	422	38	148	88	671			x			
x	I	423	28	143	85	668			x			
x		424	21	143	85	668			x			
x		425	18	142	85	668			x		articulation Vol	
x		426	16	149	87	670			x		quartz	
x	I	427	6	142	85	668			x		os	
x	F	428	34	147	87	670	EO	0	x		"	
x	I	429	34	143	90	673	—	4	x		"	
		430	32	147	88	671	NS	0	x		silex	
x		431	31	141	87	670	-	-	x		5 fragm os FAUNE	

FERRASSIE 73

Le : 29 8 / 73

Page N° 19

CARRÉ : 1

Nom et adresse :

Visées faites sur : △ = 583

= m.

Observations : (Y a-t-il un relevé, commentaire, photo, ?)

	M ou A	N°	X	Y	Z	△ ± Z	Orientation	Pendage	Dimensions	Croquis forme	Désignation et nature	Niveau ou sol
x		432	27	138	87	670	Eo	4	X	X	os cranis	M2
x		433	16	141	~~90~~86	~~673~~669	Eo	-	X		2 petits os.	"
x	F	434	24	153	~~88~~89	~~671~~672	Eo	"	4,5 cm	X	os	
x	I	435	23	151	~~87~~89	~~670~~672	"	"	X		bt os	
x	I	436	11	162	~~87~~89	~~670~~672	Eo NS	"		X	" cranis ?	
x		437	21	139	~~86~~86	669	"	"	"	X	serie os crane {	
x	I	438	-2	148	87	670	Eo	0		X	os [illegible]	
x	I	439	-7	148	87	670	NS	0	X		"	
x	I	440	-15	148	87	670	-	-	X		"	
x	I	441	-25	156	87	670	NS	0	X	s/chaur	"	
x	F	442	-20	164	87	670	NO/SE	0	X		"	
x	I	443	13	157	90	673		0	X	os collés	" fgt (crâne ?)	
x		444	35	140	89	672	-	-	X		crânes en bas 3 petits os FAUNE F	
x		445	20	147	90	673	-	-	X		os crane	
		446	20	146	"	673	Eo	0	X		silex	
x	F	447	17	142	87	670	-	-	X		os crâne	
x	I	448	7	159	92	675	-	-	X		3 os (+5 ??)	
		449	3	153	89	672	NS	0	X		silex croche	
x	F	450	-10	153	91	674	SE/NW	0	X		os long (ést ??)	
		451	-10	154	91½	674½	-	-	X		silex	
		452	16	160	90	673		0	X		silex	
x	I	453	17	162	90	673		2	X		fgt os	
		454	15	166	96	679		4	X		silex	
x		455	6	157	86	669			X		fgts os (4)	

		F73		
Le : 30 / 8 / 73	Page N° 20	CARRÉ : 1	Visées faites sur : △ = 583	= m.

Nom et adresse :

Observations : (Y a-t-il un relevé, commentaire, photo, ?)

M ou A	N°	X	Y	Z	△ ± Z	Orientation	Pendage	Dimensions	Croquis forme	Désignation et nature	Niveau ou sol
	456	5	152	90	673	EW		x		côte	N'2
I	457	3	161	88	671			x		fgt pt os ~~[illegible]~~	
I	458	-2	166	91	674		0	x		fgt crâne ?	
I	459	-5	182	92	675			x		fgt os	
I	460	-15	183	92	675			x		3 os	
I	461	-20	179	96	679	~~EW~~	~~[illegible]~~	x		os	
F	462	-28	173	95	678	EW	35	x		gd os	
F	463	-26	166	91	674			x		os (2)	
F	464	-20	161	90	673	NS	3N	x		gd os	
I	465	20	137	93	676			x		petit os	
	466	~~14~~	139	92	675			x		silex	
	467	12	141	89	672			x		petit os	
I	468	5	141	87	670			x		"	
I	469	8	147	90,5	673,5			x		petit os	
F	470	4	150	94	677	EW		x		côte ?	
I	471	~~3~~	144	91	674			x		silex	
	472	6	136	89,5	672,5	EW	4	x		os	
I	473	-4	148	95	678			x		2 os	
I	474	-9	150	95	678			x		os plat	
I	475	-16	147	94	677			x		os	
I	476	-20	146	93	676			x		os pt	
	477	-18	156	97	680			x		os	
I	478	11	141	92,5	675,5			x		pts os	
I	479	~~9~~	144	93	676			x		silex	

F73

Le : 30/ 8/ 73

Page N° 21

CARRÉ : 1

Nom et adresse :

Visées faites sur : △ = 583

= m.

Observations : (Y a-t-il un relevé, commentaire, photo, ?)

M ou A	N°	X	Y	Z	△ ± Z	Orientation	Pendage	Dimensions	Croquis forme	Désignation et nature	Niveau ou sol
I	480	10	148	96	679	EO		X		os	
I	481	5	146	96	679	NS		X		os	
I	482	10	147	95	678	NE/30	0	X		os	
I	483	8	145	94	677			X		articulation	
	484	9	137	92	675			X		idem V	
	485	7	138	92	675			X		cote ? fragm	
	486	-6	142	91	674	NS		X		cote ? fragm	
F	487	-14	150	94	677	EO		X		os	
	488	-13	140	92	675	NS		X		os FAUNE	F
I	489	9	144	95	678			X		os	
I	490	7	139	94	677	EO		X		os	
	491	5	141	93	676			X		cote	
	492	0	136	93	676			X		articul. V	
	493	0	139	93	676			X		cotes fragm	
	494	∅	144	96	679			X		silex	
I	495	10	150	96	679			X		os plat	
I	496	-16	154	96	679			X		articul.	
	497	0	134	94	677	Eo		X	X	fgts cote + articul. V	
I	498	-7	140	95	678			X		os	
	499	-9	132	95	678					articul V	
F	500	-11	142	95	678			X		2 fgts os + articul	
F	501	-12	151	97	680	NS		X		os long	
	502	-20	141	95	678			X		2 fgts phalange ?	
I	503	0	135	95,5	678,5			X		OS ([illegible])	

Page N° 22

F 73

Le : 30, 8, 73

CARRÉ : 1

Nom et adresse :

Visées faites sur : △ = 583

= m.

Observations : (Y a-t-il un relevé, commentaire, photo, ?)

	M ou A	N°	X	Y	Z	△ ± Z	Orientation	Pendage	Dimensions	Croquis forme	Désignation et nature	Niveau ou sol
x		584	0	132	95,5	678,5		–	x		rondelle V	M2
x		5	-5	144	98	681		0	x	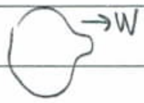	bassin	
x		6	-6	139	97	680		–	x		plt os V	
x		7	-6	148	98	681		θ	x		" V	
x	I	8	-10	146	97,5	680,5		–	x		os plat	
x	I	9	-19	159	98	681		–	x		os	
x	I	590	-6	136	95	678			x		os	
x		1	-5	137	95	678			x		rondelle V	
x		2	-3	140	97	680			x		" V	
x		3	0	141	98	681			x		petit os (épiphyse)	
x		4	-5	143	97	680			x		" "	
x		5	-5	144	99	682			x		frgt rondelle V	
x		6	-7	143	99	682			x		2 plts os V	
x		7	-11	141	99	681			x		1 plt os V	
x	F	8	-13	139	97,5	680,5	NE/SW		x		os	
x		9	-18	141	100	683	NW/SE		x		1e frgt os	
+		320	-28	153	98	681		0			silex	
x		1	-50	114	96	679			x		os FAUNE	
x	I	2	-14	140	96	679			x	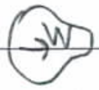	2e petit bassin	
x	±	3	0	143	97	680		0	x		rondelle V	
x	I	4	-9	139	97	680			x		esquille os	
x		5	-8	141	97	680			x		attache ? V	
x		6	-9	143	98	681			x		" ? V	
x		7	-9	145	98	681			x		os ? V	

F 73	Le : 30 / 8 / 73	Page N° 23
		CARRÉ : 1

Nom et adresse :

Visées faites sur : △ = 583

= m.

Observations : (Y a-t-il un relevé, commentaire, photo, ?)

	M ou A	N°	X	Y	Z	△ ± Z	Orien-tation	Pen-dage	Dimensions	Croquis forme	Désignation et nature	Niveau ou sol
x	I	528	-19	141	98	681			x		2 fgt n plats	
x	I	9	-24	141	97	680			x		1 " " "	
x	I	530	-27	151	97	680			x		3 fgt n	
x	I	1	-23	156	98	681			x		1 – –	
x		2	-27	146	99.5	682.5			x		3 – –	
x		3	-27	153	101	684			x		2 – —	
x	I	4	-30	153	101	684			x		1 — –	
x	I	5	-33	154	101	684			x		1 — —	
x		6	tamisage [illegible]								[illegible] ?	
	I	537	35	145	87	670				± 10	ensemble tamisé	
		8	35	140	88	671	EW	S↓			— crâne	
		9										
		540										
		1										
		2										
		3										
		4										
		5										
		6										
		7										
		8										
		9										
		550										
		1										
